# Fruit transpiration drives interspecific variability in fruit growth strategies

**DOI:** 10.1093/hr/uhac036

**Published:** 2022-02-19

**Authors:** Federica Rossi, Luigi Manfrini, Melissa Venturi, Luca Corelli Grappadelli, Brunella Morandi

**Affiliations:** Department of Agricultural and Food Sciences, University of Bologna, V.le Fanin 44, 40127, Bologna (Italy); Department of Agricultural and Food Sciences, University of Bologna, V.le Fanin 44, 40127, Bologna (Italy); Department of Agricultural and Food Sciences, University of Bologna, V.le Fanin 44, 40127, Bologna (Italy); Department of Agricultural and Food Sciences, University of Bologna, V.le Fanin 44, 40127, Bologna (Italy); Department of Agricultural and Food Sciences, University of Bologna, V.le Fanin 44, 40127, Bologna (Italy)

## Abstract

Fruit growth is a complex mechanism resulting from biochemical and biophysical events leading water and dry matter to accumulate in the fruit tissues. Understanding how fruits choose their growth strategies can help growers optimizing their resource management for a more sustainable production and a higher fruit quality. This paper compares the growth strategies adopted by different fruit crops, at different times during the season and relates their fruit surface conductance to key physiological parameters for fruit growth such as phloem and xylem inflows as well transpiration losses. Our results show how fruits capacity to transpire (determined by their surface conductance) is a key driver in determining the growth strategy adopted by a species and explains the inter-species variability existing among different crops. Indeed, fruits change their surface conductance depending on the species and the phenological stage. This has an impact on the fruit’s ability to lose water due to transpiration, affecting fruit pressure potential and increasing the force with which the fruit is able to attract xylem and phloem flows, with a considerable impact on fruit growth rate.

Perennial fruit crops are highly exposed to the negative effects of climate change due to several reasons, including increasing water scarcity and evapotranspiration requirements, leading to drought and heat stresses, with negative impacts on the whole fruit chain [1–3]. These conditions tend to reduce fruit growth potential, thus making it more difficult for growers to meet the quality standards required by the market in terms of fruit size and productivity. For these reasons, there is an increasing need to select fruit varieties more resistant to drought stress and heat waves, since these are not available on the market yet. Therefore, understanding the mechanisms underpinning fruit growth is fundamental for the development of adaptation strategies allowing the fruit sector to face current and future challenges.

Fruit growth depends on the balance between xylem and phloem inflows and/or water losses due to transpiration and xylem backflow [4]. In some fruits, assimilates move to the cell symplast through plasmodesmata, thanks to turgor pressure (mass flow) and/or concentration gradients (diffusion) [5–8]. However, when water potential gradients between phloem and sink cells is null or limited, an apoplasmic step is needed and phloem unloading is driven by an active mechanism, that relies on specific transmembrane carbohydrate transporters [9–12]. On the other hand, fruit xylem flow brings water and mineral elements to the fruit, based on both water potential gradients ($\varDelta \uppsi$) and the hydraulic conductance of the xylem-to-fruit pathway (K) [13]. In the event that leaves water requirements are so high to decrease the stem water potential to more negative values than those of the fruit, some species like apple [14], kiwifruit [7] and grapevine [15], are subjected to water losses due to xylem backflows. This phenomenon occurs mainly during the mid-part of the season, when the xylem is still functional but subsequently it is reduced to 0 close to harvest due to a loss in xylem functionality [13]. Such a loss of functionality can be due to stretches in the fruit xylem tissues, as it occurs in apple [16], or to other types of occlusions or embolisms, or simply to lack of water potential gradients [15]. Nonetheless, the main fruit water losses are due to transpiration, which depends both on the water vapour permeability of the fruit epidermis (fruit surface conductance – g_c_) and to the environmental conditions (Vapour Pressure Deficit – VPD) [17–20]. Despite transpiration water losses reduce the fruit water balance, they positively influence the fruit’s ability to attract xylem and phloem flows, since they reduce fruit pressure potential, thus potentially increasing stem-to-fruit $\varDelta \uppsi$ [13].

As reported in literature, several studies are available on the growth mechanisms of individual fruit species, showing how fruit growth strategies change not only depending on the species, but also on the basis of the phenological stage considered. For example, apple fruit maintain an apoplasmic phloem unloading during the whole season, while its transpiration and xylem functionality decrease along with fruit development [14]. On the contrary, peach fruit maintain very high water flows by xylem and transpiration up to harvest [6], while kiwifruit behave like peach, maintaining high water flows by xylem and transpiration during the youngest stages and more similarly to apple, with low or almost absent transpiration and xylem flows, closer to harvest. With this wide range of case studies, the question arising is why fruits grow according to different mechanisms? To date, studies on fruit growth strategies have been limited to only a few crops and have addressed one single species at time, while no studies are available on the anatomical traits determining their functional differences.

The aim of this work is to investigate on the existence of general relationships in the vascular flow physiology underpinning the fruit growth of different crops. To do so we have focused on the physiological traits of five of the mostly cultivated fleshy fruits (peach, apple, kiwifruit, pear and cherry). Results allow to explain why different species grow according to different biophysical mechanisms and provide hints to breeders and growers for the selection of new varieties adapted to climate change and for optimizing the orchard management inputs.

## Results and discussion

**Figure 1:**
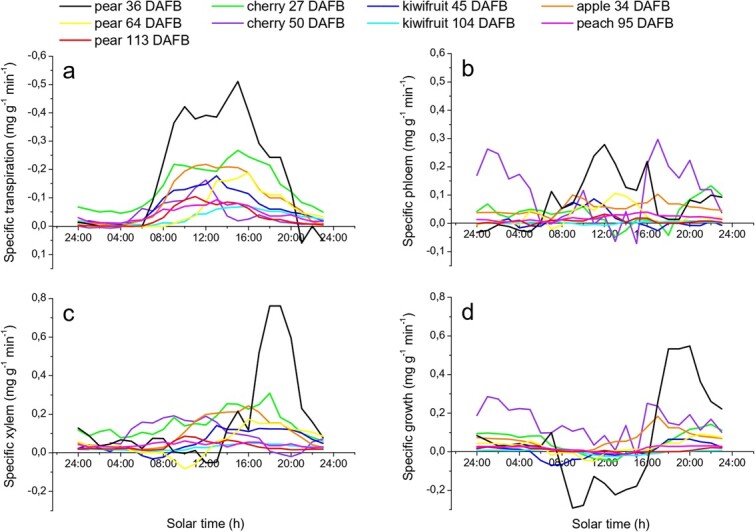
**Diurnal patterns of transpiration flow rates (a), phloem flow rates (b), xylem flow rates (c) and growth rates (d).** Each color line represents a different fruit species at different time of the season, expressed as days after full bloom (DAFB). Data were recorded at 15-min intervals during the trials and hourly averages are reported. All values are the means of at least 4 replicates and are expressed in mg g^−1^ min^−1^. See Supplementary Tab. 1 for maximum SEs.

According to literature, fleshy fruits have different growth strategies [6–8]. However, by comparing the daily patterns of several fruit species at different times during the season it is evident how within a single crop, fruit growth rates, vascular and transpiration flows vary also depending on the phenological phase considered (Fig. 1). In general, on a daily scale, transpiration water losses increase from dawn to mid-afternoon when daily peaks are reached. In particular, for the trials presented in this paper, young fruit showed the highest transpiration rates with values of −0,512 mg g^−1^ min^−1^ for pear at 36 DAFB, −0,268 mg g^−1^ min^−1^ for cherry at 27 DAFB, −0,218 mg g^−1^ min^−1^ for apple at 34 DAFB and − 0,178 mg g^−1^ min^−1^ for kiwifruit at 45 DAFB (Fig. 1a). Daily phloem peaks were mainly observed around noon, while lower flow rates were recorded from the evening to the early morning (Fig. 1b). In general, xylem flow maintains low values during the early part of the morning while it increases from midday on, reaching its maxima in the late afternoon, after transpiration had already reached its peak (Fig. 1c). This is due to the fact that in the morning, stem-to-fruit water potential gradients are usually lower than stem-to-leaf and most of the water is direct to transpiring leaves. In some species, at this time of the day, xylem inflow values are not even sufficient to balance transpiration water losses [14]. Therefore, xylem backflows (i.e. negative xylem flow values) appears, such in the case of kiwifruit at 45 DAFB, pear at 36 and 64 DAFB. (Fig. 1c). Occasional negative phloem flows also appeared for some crops (Fig 1b). In this case, it is difficult to state whether they are due to negative pressure potential gradients between the fruit and the phloem or to measuring artifacts. In fact, while xylem backflows are known as normal events in the daily water balance of some fruit (e.g. apple) [14], to our knowledge, phloem backflows have never been reported in literature.

The daily pattern of fruit growth is the result of the balance between total inflows (xylem and phloem) [6] and outflows (transpiration and sometimes xylem backflows) and, depending on the time of the day and the relative balance, it can alternate periods of swelling (positive growth values) which occurs mainly during the late afternoon and night, and shrinkage (negative growth values) during the morning until the early afternoon [6,14,21,22], as in the case of pear at 36 DAFB, kiwifruit at 45 DAFB and cherry at 27 DAFB (Fig. 1d).

**Figure 2:**
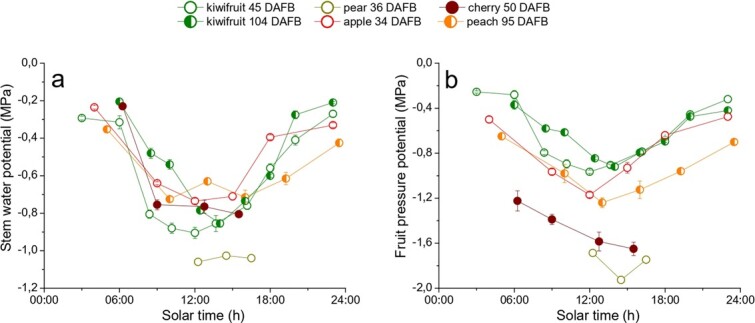
**Daily patterns of stem (a) and fruit water potential (b).** Each color represents a different fruit species at different time of the season. Each point represents the mean (± SE) of at least 4 replicates.

The transport of water and assimilates to growing fruit is affected by tree water relations [23]. For this reason, daily and seasonal changes in fruit development are influenced by variations in stem and fruit water potentials. In general, water potentials tend to decrease at midday with a recovery during the afternoon and night hours [7,8,24], but differences stand out among and within the fruit species (Fig. 2). The lowest stem water potentials values are reached around midday by pear at 36 DAFB (−1,0 MPa) and kiwifruit at 45 DAFB (−0,9 MPa), while peach at 95 DAFB is the fruit species that decreases less its stem water potential during the day. With regard to fruit pressure potential, minimum values are reached in the early afternoon by pear at 36 DAFB (−1,9 MPa) and cherry at 50 DAFB (−1,7 MPa). The exceptions are apple at 34 DAFB and kiwifruit at 45 DAFB, as their fruit pressure potential show the highest value at noon (−1,2 and − 0,9 MPa, respectively). However, these values might hardly be comparable since the experiments were conducted in different sites and environmental conditions.

## Seasonal fruit growth strategies and fruit anatomical features

**Figure 3:**
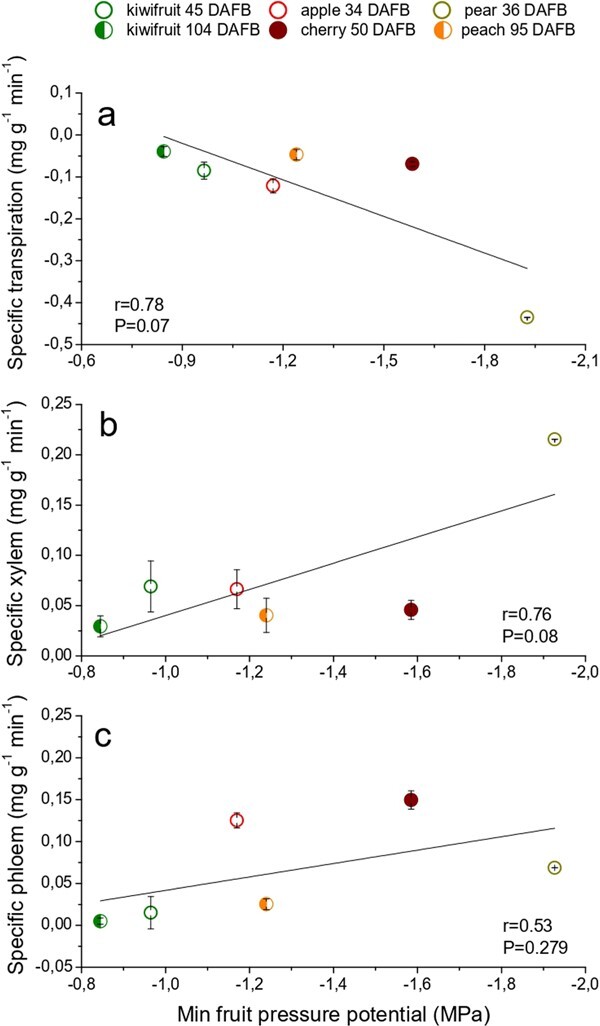
**Cross-species relationship between minimum daily fruit pressure potential and daily average specific transpiration rate (a), xylem (b) and phloem flow (c).** Each point represents a different fruit species at a different phenological stage. Fruit vascular and transpiration flows represent the average at the time of pressure potential measurements. Standard errors are reported as vertical bars. Where error bars are not visible, they are contained within the symbol. The list of the datasets used for this study is reported in Table 1 and Table 2 (see Methods).

Fruit transpiration showed an almost significant relationship with minimum daily fruit pressure potential (P = 0.07) (Fig. 3a) suggesting how fruit pressure potential can be negatively affected by transpiration rate [13]. As a consequence, the more the fruit lowers its water potential, the more it might be able to attract water through the xylem flow (Fig. 3b) due to a greater stem-to-fruit pressure potential gradient, which the fruit can exploit to attract xylem and phloem sap [7,23]. However, as suggested by the weak correlation between xylem inflows and fruit pressure potential (P = 0.08) other factors such as embolisms or structural dysfunctionalities might influence the xylem inflows to the fruit thus inducing some variability on this response. Weak correlations were also found between minimum daily fruit pressure potential and phloem flow (Fig 3c). Indeed, only the passive phloem unloading strategy should be influenced by low fruit pressure potential, as the active one is driven by a biochemical mechanism which involves specific transporters to unload carbohydrates [5,9,25]. These considerations support the independence of the apoplastic phloem unloading that characterize some fruit species from the fruit pressure potential. Unfortunately, for some database, fruit pressure potential was not available, so further investigations are needed to better understand how this parameter influences vascular flows as the season progresses.

### Water losses by transpiration

As seen, water losses due to fruit transpiration seem to induce a decrease in fruit pressure potential. Although this relationship is only marginally significant (Fig. 3a), it suggests how fruit transpiration might contribute to drive the path of vascular flows. This confirms what has been found also in peach where bagging significantly reduced fruit transpiration and thus fruit xylem inflow [26].

**Figure 4:**
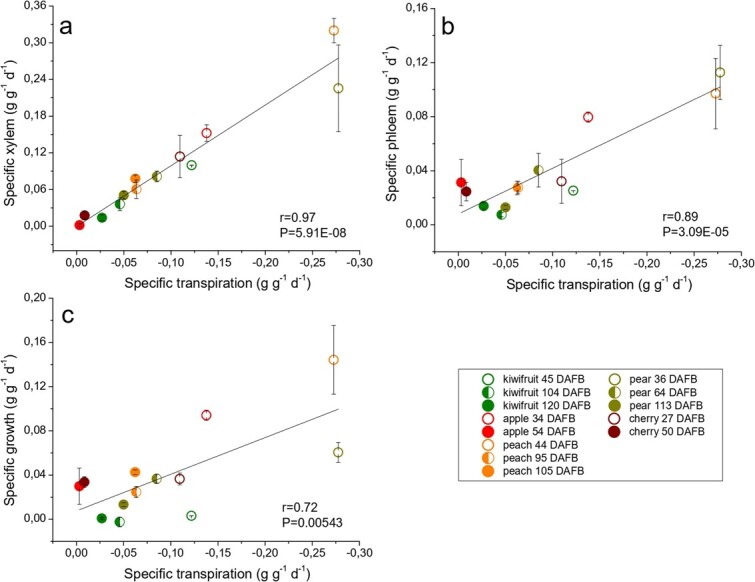
**Cross-species relationships between fruit transpiration and xylem flow (a), phloem flow (b) and specific growth (c).** Each point represents a different fruit species at a different time of fruit development. Standard errors are reported as vertical bars. Where error bars are not visible, they are contained within the symbol. The list of the datasets used for this study is reported in Table 1 and Table 2 (see Methods).

In order to understand how fruits can choose their own growth strategy, relationships concerning transpiration were deeply investigated. Considering all species and phenological stages, specific transpiration rate is positively related to xylem flow, with *r* value of 0.97 and *P* value of 5.91E-08 (Fig. 4a). This must be due to the fact that the transpiration water losses allow to lower the fruit pressure potential, favoring the translocation of xylem towards the fruit tissues, regardless of the species considered and the phenological stage [4,8]. Our data also show how transpiration rate is linearly related to phloem flow, with *r* value of 0.89 and *P* value of 3.09E-05 (Fig. 4b). Water potential gradients between the phloem and fruit tissue allow the phloem to be unloaded passively [5]. This condition is favored by a high transpiration, as it involves a greater fruit shrinkage and a reduction of pressure potential during the day.

In some fruit species, the phloem unloading pathway shifts from symplastic to apoplastic during the season, or the two mechanisms can coexist during fruit development [8,27]. For this reason, there is an increased probability for passive phloem unloading to occur in fruit with high transpiration rates_._ Indeed, low transpiring fruits such as apple at 54 DAFB [10], cherry at 50 DAFB, pear at 113 DAFB [12], and probably kiwifruit during the final stages [7] are characterized by an active phloem unloading, while symplastic phloem unloading is more likely to occur in high transpiring fruit, such as pear at 36 DAFB [8], peach at 44 DAFB [6] and presumably apple at 34 DAFB and kiwifruit at 45 DAFB [7] (Fig. 4b). Transpiration water losses are also well correlated with growth rate, with a *r* value of 0.72 and *P* value of 0.00543 (Fig. 4c), as the growth is the result of xylem and phloem flows [6]. Therefore, from the results obtained, it is possible to state that the anatomical capacity of the fruit to transpire drives the fruit growth strategies.

### Fruit surface conductance

**Figure 5:**
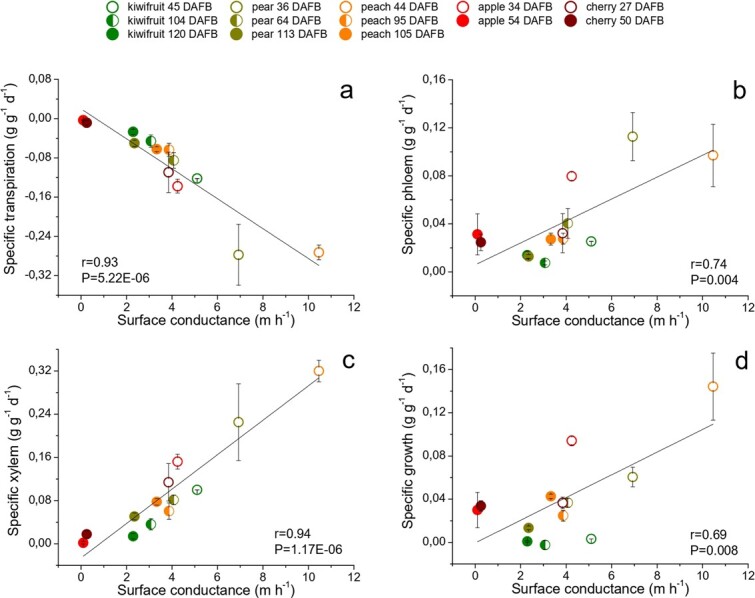
**Cross-species relationships between fruit surface conductance and transpiration (a), phloem (b), xylem (c) flows and daily fruit growth (d)**. Each point represents a different species at a different time of fruit development. Standard errors are reported as vertical bars. Where error bars are not visible, they are contained within the symbol. The list of the datasets used for this study is reported in Table 1 and Table 2 (see Methods).

The epidermis fruit surface conductance to water vapour, is an important variable both for fruit development and quality since it is an anatomical trait that controls transpiration water loss [17,20]. In fact, our data show how fruit surface conductance is strongly related to specific transpiration, with *r* value of 0.93 and *P* value of 5.22E-06 (Fig. 5a). In particular, a higher fruit surface conductance leads to a higher amount of water losses by transpiration. This is especially true for young fruits, which are characterized by a high specific transpiration rate and intense epidermis permeability to water vapour (i.e. high g_c_) [17,20,28,29], also increased because of their small size which determines a higher surface/volume ratio [13]. This is the case for peach at 44 DAFB, pear at 36 DAFB, kiwifruit at 45 DAFB, apple at 34 DAFB and cherry at 27 DAFB (Fig. 5a). Later in the season, fruit development leads to a progressive reduction in fruit surface conductance and thus their transpiration rates, for all species considered. This phenomenon is generally attributed to the accumulation of waxes and/or to the drop in fruit stomatal functionality [20] and has been already described in some fruit like kiwifruit [28], apple [17], peach [20], grape [29] and cherry [30]. In this study the same results emerge, especially in the case of apple, cherry, kiwifruit and pear at 54, 50, 120 and 113 DAFB, respectively (Fig. 5a). Fruit with a better transpiration, such as those in the early-stage, are affected by a lowering of fruit pressure potential and consequently an increase in fruit capacity to attract phloem and xylem flows [13]. Nevertheless, cross-specific changes in fruit surface conductance affect phloem flow to a lesser extent, with *r* value of 0.74 and *P* value of 0.004 (Fig. 5b). This weaker relationship is likely due to the complex process of phloem unloading, which is regulated both by biophysical and biochemical factors [5]. Indeed, for some species, like apple at 54 DAFB and pear at 113 DAFB, specific transmembrane carbohydrates transporters have been found to drive phloem unloading with the help of an apoplasmic step [10,12]. Therefore, in these crops carbohydrate unloading is not only dependent on the presence of phloem-fruit hydrostatic pressure gradients [5]. On the other hand, xylem sap has a greater linear relationship with surface conductance, with *r* value of 0.94 and *P* value of 1.17E-06, with peach at 44 DAFB, pear at 36 DAFB and kiwifruit at 45 DAFB showing the highest values (Fig. 5c). Moreover, xylem inflow is a key determinant for fruit calcium uptake, therefore fruits at the beginning of the season are able to increase the accumulation of this element, which is fundamental for fruit quality and storage potential [13,31,32]. As the fruit develops, xylem flows decrease and also the g_c_ values. According to literature, this is especially true for species like apple at 54 DAFB [16], cherry at 50 DAFB, kiwifruit at 120 DAFB [33,34] and pear at 113 DAFB [8] (Fig. 5c).

As seen, the higher fruit surface conductance and transpiration rate can lead to decreases in the fruit pressure potential, thus increasing the force with which the fruit is able to attract xylem and phloem flows, with also a considerable impact on fruit relative growth rate (RGR). This leads to a positive linear relationship between fruit surface conductance and fruit specific growth, with *r* value of 0.69 and *P* value of 0.008 (Fig. 5d).

**Figure 6:**
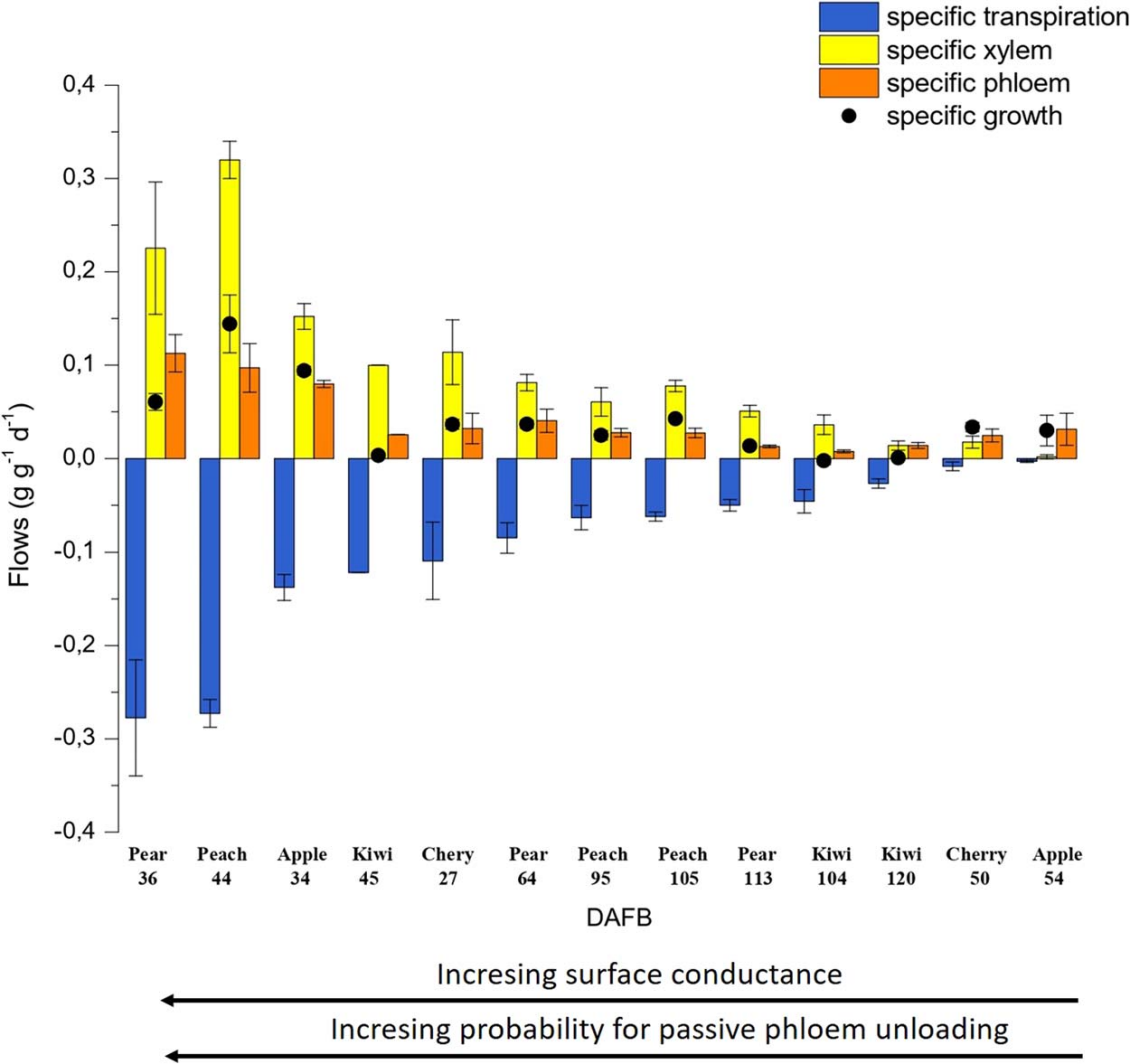
Specific daily growth (black circles), transpiration (blue bars), xylem (yellow bars) and phloem (orange bars) flows (±SE) to/from the fruit of different species, at different times during their development. The time of the season to which data are referred is indicated as days after full bloom (DAFB). The species/phenological stages considered in this paper are ranked based on their transpiration rate, from the case studies with the highest transpiration (right side) to the ones with the lowest transpiration (left side).

Considering the different case studies presented in this paper and ranking them according to their transpiration rate (Fig 6) we can classify fruits in different groups, according to their vascular growth strategy:

i) young fruits characterized by high surface conductance and consequently high specific transpiration and xylem flows to/from the fruit, such as pear at 36, peach at 44 and apple at the beginning of the season. The RGR of these fruits as well as their phloem inflow are generally high, with some exceptions (e.g. kiwifruit in this case, showing). These conditions might favour mechanisms of symplasmic (passive) phloem unloading, coexisting with apoaplsmic (active) phloem unloading;
ii) fruit characterized by middle surface conductance with medium-level transpiration and xylem flows as well as variable daily RGRs and phloem inflows. Some of these fruits can be in their middle or even mature stage as it is the case for peach and pear. Apparently, these species do not lose their xylem conductivity even at ripening stage, while their phloem unloading might be supported by both apoplasmic and symplasmic strategies. Indeed, the presence of active transporters has been demonstrated in peach [35] and in pear [12];
iii) fruit characterized by very low surface conductances and consequently low transpiration rates such as mature apples and kiwifruit. Xylem inflows to these fruits are in general very low, sometimes due to physical disruptions in the xylem vessels [16], while fruit RGR is totally sustained by phloem inflows. In this case, phloem inflows are very likely to occur only thanks to the presence of active transporters, through apoplasmic phloem unloading as demonstrated for apple [10].

However, while clear relationships were found between fruit surface conductance and fruit vascular flows, our data do not provide evidence on the type of phloem unloading strategies adopted by the different crops.

## Conclusions

Fruit are the economic target of the production and their growth is the result of many physiological processes going on at plant level [36]. To our knowledge, this is the first study detailing an overview of fruit growth strategies, highlighting how fruit development mechanisms during the season depend on their transpiration capacity. Our data show how fruit surface conductance is able to regulate tree water relations and the pathway of vascular flows. In particular, we found how fruit with higher surface conductance can attract more water (and likely mineral elements) from the xylem and create the physiological condition to sustain a passive phloem unloading. On the other hand, fruit with low surface conductance tend to lower water exchanges due to transpiration, leading to an apoplasmic phloem flow. Results also show how young fruits tend to lose a specifically higher amount of water by transpiration, unload more xylem sap and have more probabilities to sustain a symplasmic phloem unloading. Therefore, the results obtained stand out fruit surface conductance, a genetically determined anatomical trait, as a key driver of fruit growth strategies.

This study explains the existing inter-specific variability in the fruit growth strategies adopted by the different crops/varieties and has the potential to provide hints on the growing mechanisms (as well as their resource needs) of all crops, whose physiology has not been studied yet. Besides, this knowledge can provide hints on how to optimize resource inputs to the orchard (i.e. by optimizing irrigation and/or by applying calcium only to fruit with functional xylem etc.). Furthermore, we believe this novel approach has the potential to be applied also to other aspects of plant physiology, highlighting further inter-specific relationships between anatomical and physiological traits on a broader scale.

## Methods

### Plant material and experimental set up

The study was carried out by analysing datasets related to different trials conducted mainly in the Po Valley, at the experimental farm of the University of Bologna (Cadriano, Bologna, Italy), where the orchards were managed according to standard cultural practices, especially with regard to irrigation, fertilization, thinning and pruning. The datasets refer to different fruit species at several stages of development expressed as days after full bloom (DAFB). Most of the trials were conducted for at least two seasons with consistent results, but for simplification purposes only one year of data per each trial is reported in this study.

The species and times of the season covered by the study are listed below:

- Apple at 34 and 54 DAFB (*Malus* x *domestica* Borkh cv. Gala);
- Peach at 44, 95 and 105 DAFB (*Prunus persica* (L.) Batsch cv. Stark Red Gold);
- Kiwifruit at 45, 104 and 120 DAFB (*Actinidia deliciosa* cv. Summerkiwi);
- Cherry at 27 and 50 DAFB (*Prunus avium* cv. Black Star);
- Pear at 36, 64 and 113 DAFB (*Pyrus communis* cv. Abbé Fétel).

For each species, the developmental stage was identified as the days after full bloom (DAFB). Temperature, relative humidity and rainfall data were available from a weather station (A840 Base Station, Adcon Telemetry GmbH, Klosterneuburg, Austria) placed on the farm and from the Arpae Simc data extraction webapp “Dext3r”. On the basis of this data, vapour pressure deficit (VPD) was calculated (Tab. 1).

**Table 1:** **List of the trials whose datasets were considered in this study.** For each species, it is indicated the cultivar, the rootstook, the training system and the plant density of the orchards considered. The time of the season to which data are referred is identified as days after full bloom (DAFB). Mean daily vapour pressure deficit (VPD) recorded during the trial is also reported

Reference	Species	DAFB	Cultivar	Rootstock	Training system	Planting density	VPD
*Morandi et al. 2011* ^37^	Apple	34	Imperial Gala	M.9	Free spindle	2381 trees ha^−1^	0,81
*Venturi et al. 2021 (unpublished)*	Apple	54	Imperial Gala	M.9	Solaxe	3800 trees ha^−1^	1,33
*Morandi et al. 2010* *^7^*	Kiwifruit	45	Summerkiwi		T-bar	1112 vines ha^−1^	1,43
*Morandi et al. 2010* *^7^*	Kiwifruit	104	Summerkiwi		T-bar	1112 vines ha^−1^	1,23
*Morandi et al. 2010* *^7^*	Kiwifruit	120	Summerkiwi		T-bar	1112 vines ha^−1^	0,96
*Morandi et al. 2007* *^6^*	Peach	44	Stark Red Gold	A6 clonal seedlings	Y trellis	1274 trees ha^−1^	0,90
*Morandi & Corelli Grappadelli 2009* ^38^	Peach	95	Stark Red Gold		Open-vase	545 trees ha^−1^	1,73
*Morandi et al. 2007* *^6^*	Peach	105	Stark Red Gold	A6 clonal seedlings	Y trellis	1274 trees ha^−1^	1,42
*Morandi et al. 2014* *^8^*	Pear	36	Abbé Fétel	Sydo quince	Central leaders	3787 trees ha^−1^	1,15
*Morandi et al. 2014* *^8^*	Pear	64	Abbé Fétel	Sydo quince	Central leaders	3787 trees ha^−1^	1,07
*Morandi et al. 2014* *^8^*	Pear	113	Abbé Fétel	Sydo quince	Central leaders	3787 trees ha^−1^	1,48
*Morandi et al. 2019* *^38^*	Cherry	27	Black Star	Gisela ™ 6	V	2470 trees ha^−1^	0,76
*Morandi et al. 2019* *^38^*	Cherry	50	Black Star	Gisela ™ 6	V	2470 trees ha^−1^	1,48

Datasets include physiological parameters, such as (i) daily fruit growth, (ii) vascular and transpiration flows, (iii) fruit surface conductance, (iv) daily patterns of fruit (Ψ_fruit_), leaf (Ψ_leaf_) and stem (Ψ_stem_) water potential (Tab. 2).

**Table 2:** **List of the physiological parameters available from the dataset considered.** For each species and phenological stage it is indicated whether the different parameters were available from previous trials carried out at the experimental farm of the University of Bologna or, in the case of pear, at “F.lli Navarra” Experimental Farm, close to Ferrara, Italy (✓). The time of the season to which data are referred is indicated as days after full bloom (DAFB)

Species	DAFB	Vascular flows and transpiration	Fruit g_c_	Ψ_Fruit_	ΔΨ_Stem_
Apple	34	✓	✓	✓	✓
Apple	54	✓	✓		
Kiwifruit	45	✓	✓	✓	✓
Kiwifruit	104	✓	✓	✓	✓
Kiwifruit	120	✓	✓		
Peach	44	✓	✓		
Peach	95	✓	✓	✓	✓
Peach	105	✓	✓		
Pear	36	✓	✓	✓	✓
Pear	64	✓	✓		
Pear	113	✓	✓		
Cherry	27	✓	✓		
Cherry	50	✓	✓	✓	✓

### Fruit growth, vascular and transpiration flows

As described by Lang [14], the balance among phloem, xylem and transpiration in/out fluxes to/from the fruit determines the variation of the fruit diameter in a finite time interval. For this study, it was assumed that girdling interrupts the phloem without affecting the xylem flux [39] and that transpiration rate is not affected by detachment in a short time period. Consequently, for all species at different phenological stages, vascular and transpiration flows were calculated as the difference between the diameter changes of intact, girdled and detached fruits.

The diameter variations of at least 4 well exposed and representative fruit per species/stage were monitored during the 24 hours. Diameter variations were measured using custom-built gauges consisting of a light, stainless steel frame supporting a variable linear resistance transducer (Megatron Elektronik AG & Co., Munchen, Germany). The gauges were interfaced with a wireless data-logger system (Wi-Net s.r.l. Cesena, Italy) [41] [42]. Data were logged every 15 minutes with a resolution in the order of 1–2 μm. Temperature effects on the frame and the sensor were tested and showed negligible errors under normal field conditions [40].

Monitored fruits were progressively subjected to the following sequence of conditions: first, they were monitored in “intact” conditions, with all vascular connections normally functional; then fruit were girdled by removing a strip of bark on each side of the peduncle, thus severing the phloem connection; eventually fruit were detached, thus severing all vascular connections. In this case the peduncle surface was covered with glue to avoid further water losses. In all conditions, fruit were left in their original position with the help of thin wire and their diameter variations were continuously monitored by the fruit gauges.

Normal and girdled fruit were monitored for one to two days, while fruit in the “detached” condition were monitored for a maximum of 24 hours to avoid excessive tissue dehydration, which could lead to an underestimation of fruit transpiration. Only data collected during clear days were used for the analyses. Control fruit in intact conditions were also maintained to verify that the daily growth pattern did not change significantly during the measurement period.

Diameter data (D) from all fruit monitored were converted to fresh weight (FW) at each monitoring time (15 minutes) through the following conversion equation (1):

(1) \begin{equation*} \mathrm{FW}\left(\mathrm{g}\right)=\mathrm{a}\ast \mathrm{D}{\left(\mathrm{mm}\right)}^b \end{equation*}

where the a and b coefficients change in relation to the fruit species considered (Tab. 3). This equation was obtained by regressing diameter and weight data of a large number (above 300) of fruit, from the same or different orchards where the experiments were set. For all datasets the R^2^ of the relationship was always >0.99.

**Table 3:** **Species-specific “a” and “b” coefficients available from the literature and used in the FW equation.** In the case of apple, kiwifruit and peach, their error standards (± SE) have also been reported

Species	A	SE a	b	SE b
Apple	0,0006	0,00005	29 029	0,0194
Kiwifruit	0,0013	0,0002	2864	0,0529
Peach	0,0023	0,00025	27 734	0,032
Pear	0,0019		27 675	
Cherry	0,0021		25 431	

The fresh weight (g g^−1^ day^−1^) daily changes were then calculated for the fruit in each of the three conditions: normal, girdled and detached. For each tree, the phloem flow was obtained as the difference between the diameter changes of normal and girdled fruit. Instead, the xylem flow was obtained by subtracting the diameter changes of detached fruit from those of the girdled ones; whereas the transpiration flow was considered equal to the diameter variations of detached fruit.

Fruit growth rate, phloem, xylem and transpiration flows were expressed as weight changes per unit of fruit weight (g g^−1^).

### Water relations

Stem and leaf water potentials, as well as fruit pressure potential, were measured with a Scholander pressure chamber (Soilmoisture Equipment Corp. Santa Barbara, USA). Leaves and fruits were sampled at predawn, at 9:00, 12:00 and 15:00 hour or even more often. Following Turner and Long [43], measurements of leaf water potential were made on four leaves (one per tree), from the outer part of the canopy. For stem water potential, four leaves on the inner part of the canopy were sampled and covered with aluminum foil at least 90 minutes prior to measurement to allow equilibration with the stem [44] [45]. Similarly, the fruit pressure potential in the xylem was measured on 4 fruit.

For apple, kiwifruit, cherry and pear, fruit pressure potential was measured simply by inserting the fruit petiole on the Scholander pressure chamber. In peach, fruit pressure potential was measured as reported by Morandi et al.^26^: about 50 mm of lignified twig, together with the fruit. The cut surface on one side of the twig was immediately covered with glue, whereas the other side was inserted in the chamber lid for measurement.

Using the method of the Scholander chamber, the pressure potential recorded on leaf and stem can be assumed to be equal to the water potential as the concentration of the xylem sap is almost zero for these organs. As for fruit pressure potential, the situation is slightly different since it may not coincide with water potential due to the fact that the osmotic concentration in the fruit apoplast may be significantly high^46^. For these reason, in this paper we refer to “pressure potential” to indicate the fruit water status.

At all recording times and for all parameters, means (± S.E.) were then computed.

### Fruit surface conductance

For each fruit species, data of fruit surface conductance (g_c_) was determined on at least 8 fruit per species, in accordance with the following equation [5], as reported by Fishman and Génard^4^:

(5) \begin{equation*} gc=\frac{Tr\times RT}{A\times Mw\times VPD} \end{equation*}

where “Tr” is fruit transpiration (g fruit^−1^ h^−1^); “R” the gas constant; “T” the temperature (°K); A the fruit surface area (m^2^); Mw the molecular mass of water and “VPD” the vapour pressure deficit (kPa).

At each measurement time, “Tr” was determined as the average fruit transpiration during the 24 hours; “VPD” was determined from the average environmental conditions (T°C and RH%) recorded during the 24 hours. Fruit “A” was estimated from the two fruit transversal diameters and from the fruit height, by simplifying the fruit shape as a geometrical volume such as a sphere for apple, cherry and peach, an ellipsoid for kiwifruit and a half sphere surmounted by a cone for pear.

### Statistical analysis

The several physiological parameters were related to each other considering the different species and times during the season. For each relationship the correlation coefficient (r) was calculated as well as the significance of the relationship (P) using OriginPro 8.1 (OriginLab, Northampton, MA, USA).

## Supplementary Material

Web_Material_uhac036

## Data Availability

We hereby declare that all data are available upon request.
