# Supplementary material for: Fruit transpiration drives interspecific variability in fruit growth strategies
Source: Hortic Res. 2022 Feb 19;9:uhac036. doi: 10.1093/hr/uhac036 (PMC8987619; doi:10.1093/hr/uhac036)
Supplement: Web_Material_uhac036 [file web_material_uhac036.zip › Supplementary Figures.docx]

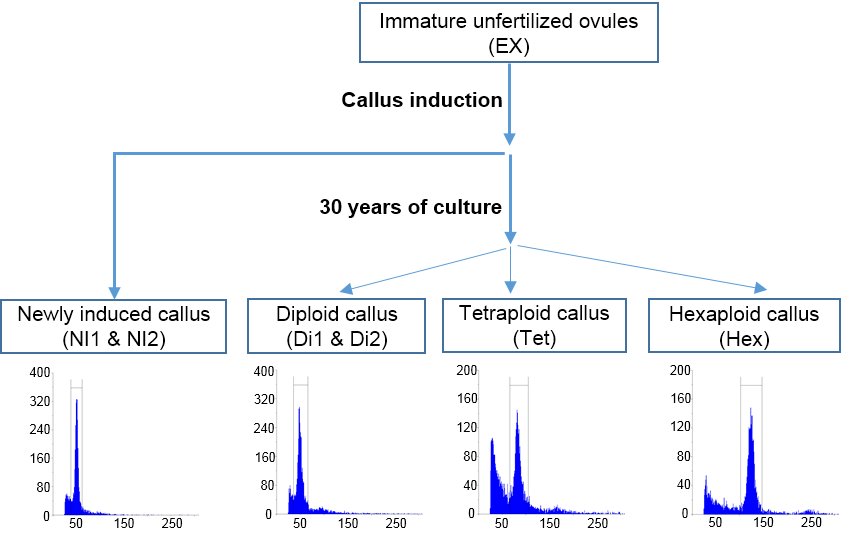


**Fig. S1 Ploidy level of materials in this study determined using flow cytometry.** EX, explant; NI1 and NI2, two independent newly induced callus lines; Di1 and Di2, two independent diploid callus lines after long-time culture; Tet, tetraploid callus after long-time culture; Hex, hexaploid callus after long-time culture.


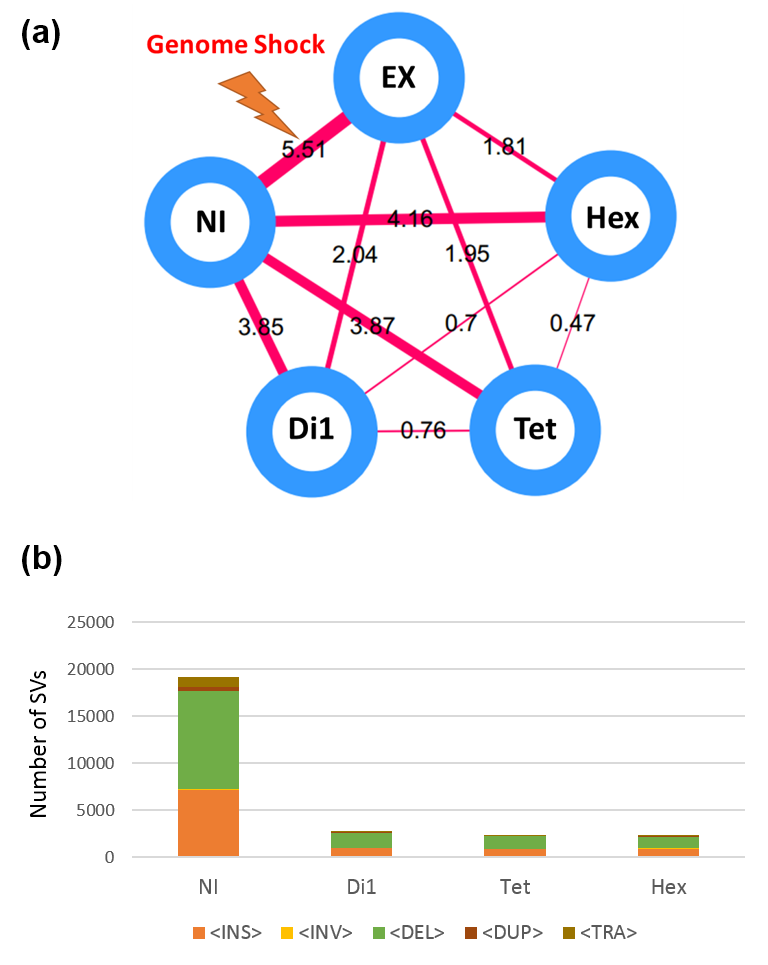


**Fig. S2 Overall SNP rates between each pair of material (a) and numbers of structure variations (SVs) in the *in vitro* cultured calli in the reference of sweet orange genome (b).** The values of SNP rate (‰) between each pair of materials are indicated on the line between the two materials, and are proportional to the line thicknesses. EX, explant; NI, newly induced callus; Di1, diploid callus from long-term culture; Tet, tetraploid callus from long-term culture; Hex, hexaploid callus from long-term culture. INS, insertion; INV, inversion; DEL, deletion; DUP, duplication; TRA, translocation.


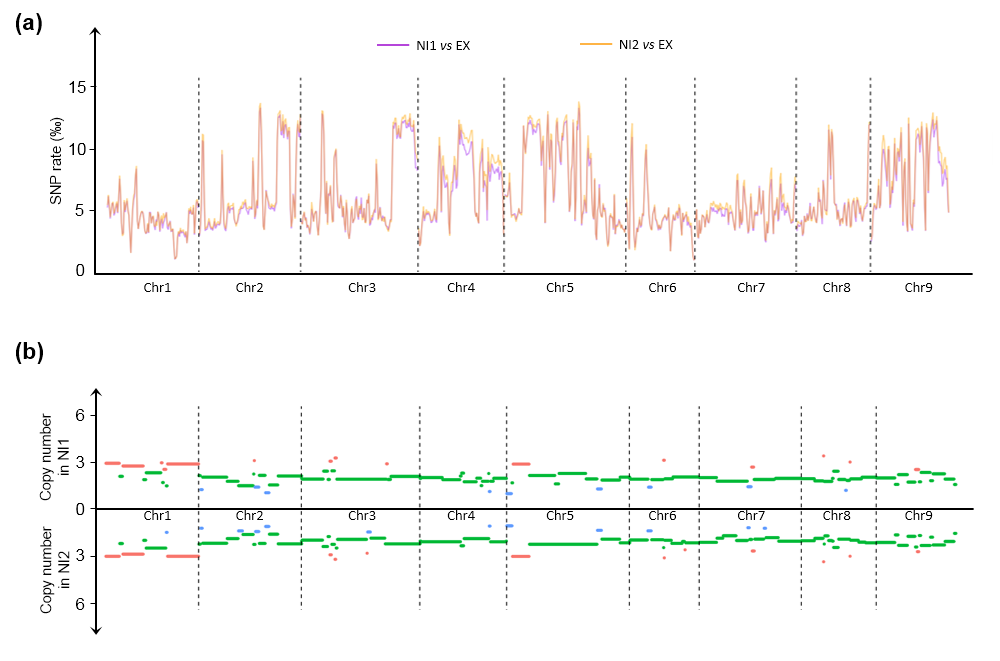


**Fig. S3 Comparison of distribution of SNP rates (a) and CNV (b) across the genome in the two replicates of newly-induced calli.** SNP rates were calculated in 500-kb sliding windows with a 300-kb step. DNA copy number were calculated in 200-kb windows. EX, explants; NI1 and NI2, two independent newly induced calli.


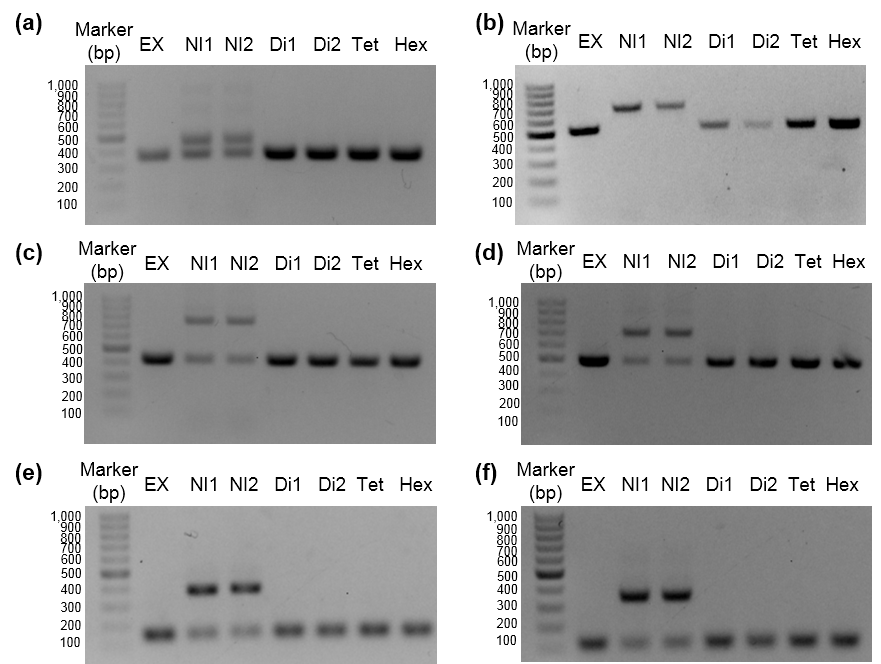


**Fig. S4 Experimental validations for TE insertions.** **a**-**f** is corresponding to TE_in_Cs2g_pb006570, TE_in_Cs2g_pb020210, TE_in_Cs4g_pb022990, TE_into_chr2:4879334, TE_into_chr8:3585305, and TE_into_chr1:23537730, respectively. The detailed information was listed in the Table S9. EX, explant; NI1 and NI2, two replicates of newly induced callus; Di1 and Di2, two independent lines of long-term cultured diploid callus; Tet, the long-term cultured tetraploid callus; Hex, the long-term cultured hexaploid callus.


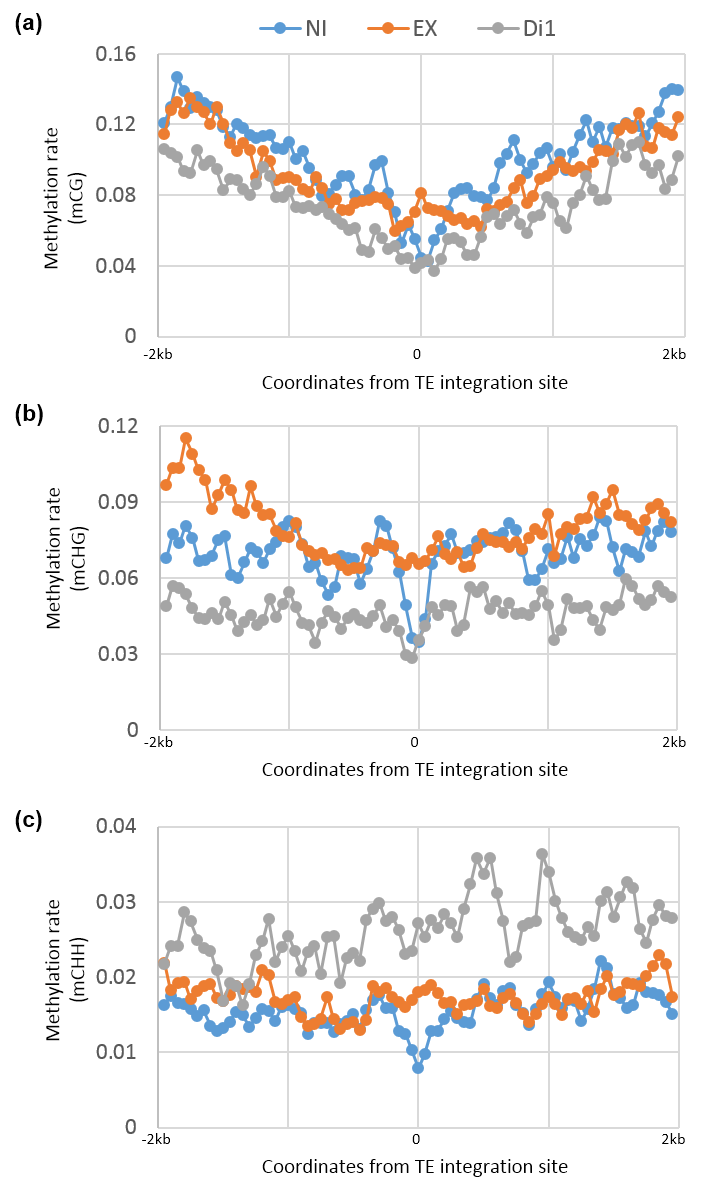


**Fig. S5 Methylation rates of regions surrounding TE integration sites in three materials.** **a**-**c** is for CG, CHG, and CHH context, respectively. EX, explant; NI, newly induced callus; Di1, diploid callus from long-term culture.


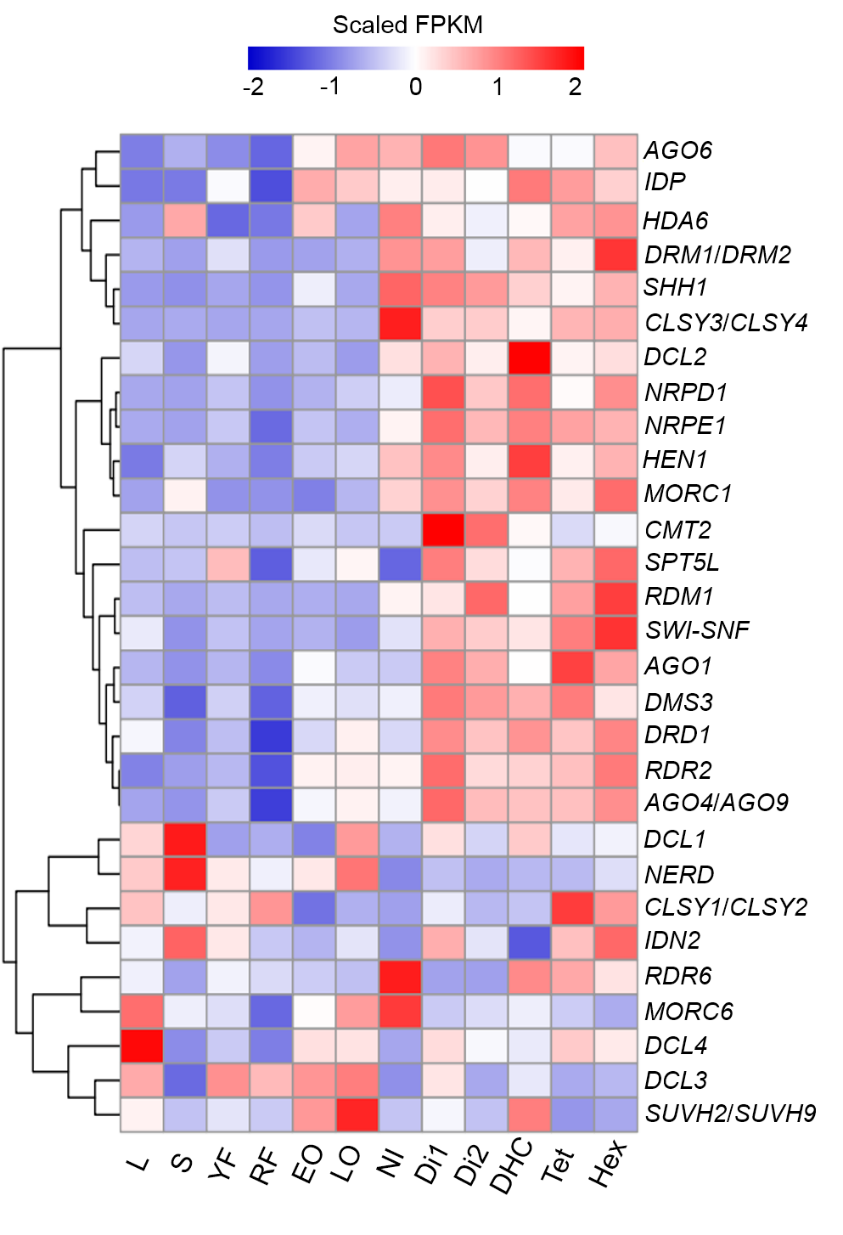


**Fig. S6 Heat map of expression levels of genes involved in RNA-directed DNA methylation (RdDM) and small RNA biogenesis in *in vitro* cultured calli and different tissues of sweet orange.** Scaled FPKM (fragments per kilobase of transcript per million mapped reads) values for each gene are shown. L, leaf; S, seed; YF, young fruit; RF, ripe fruit; EO, early-stage ovule; LO, late-stage ovule; NI, newly induced callus, Di1 and Di2, two independent diploid calli from long-time culture; DHC, Tet, and Hex, double-haploid callus, tetraploid callus, and hexaploid callus from long-time culture.


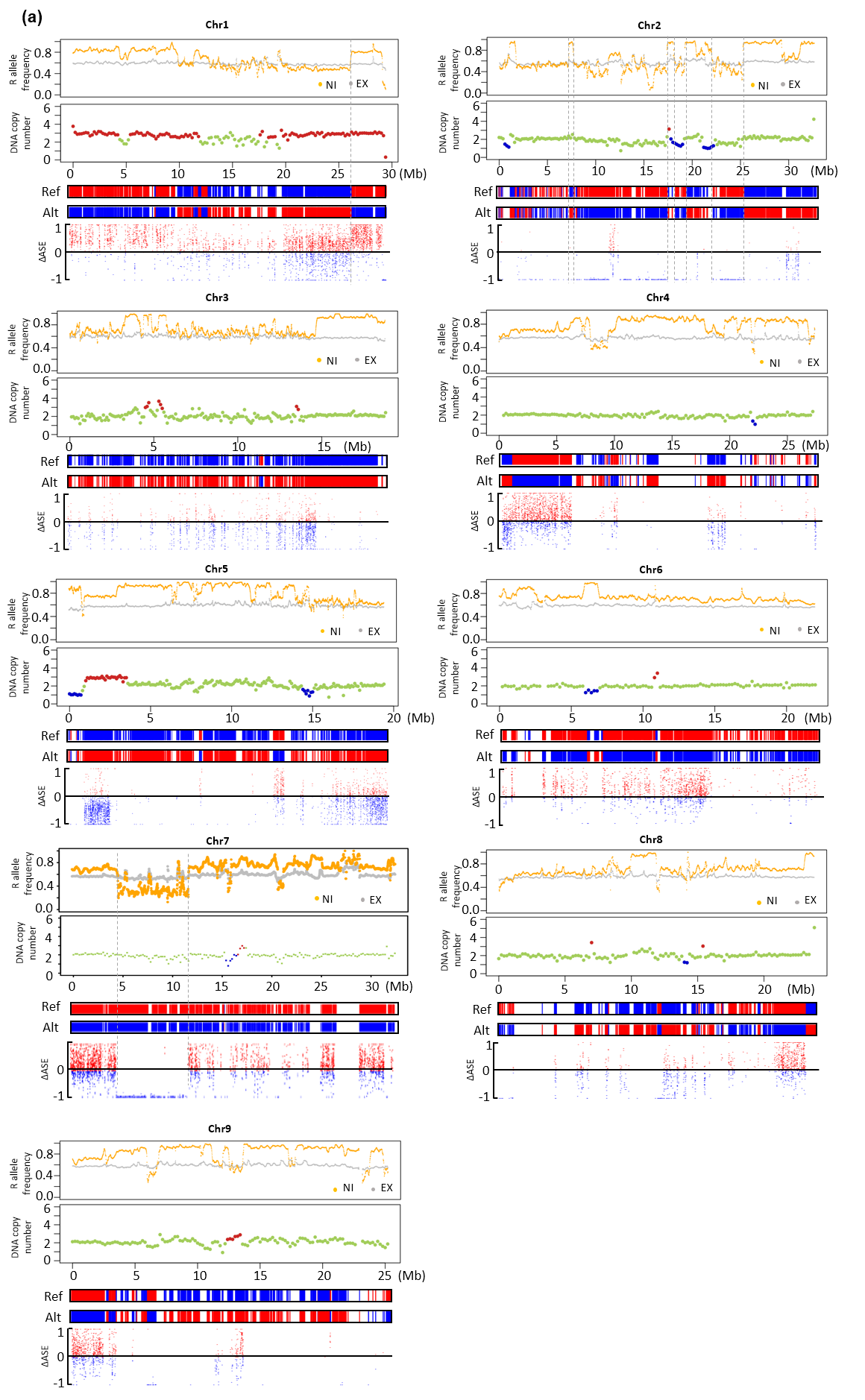


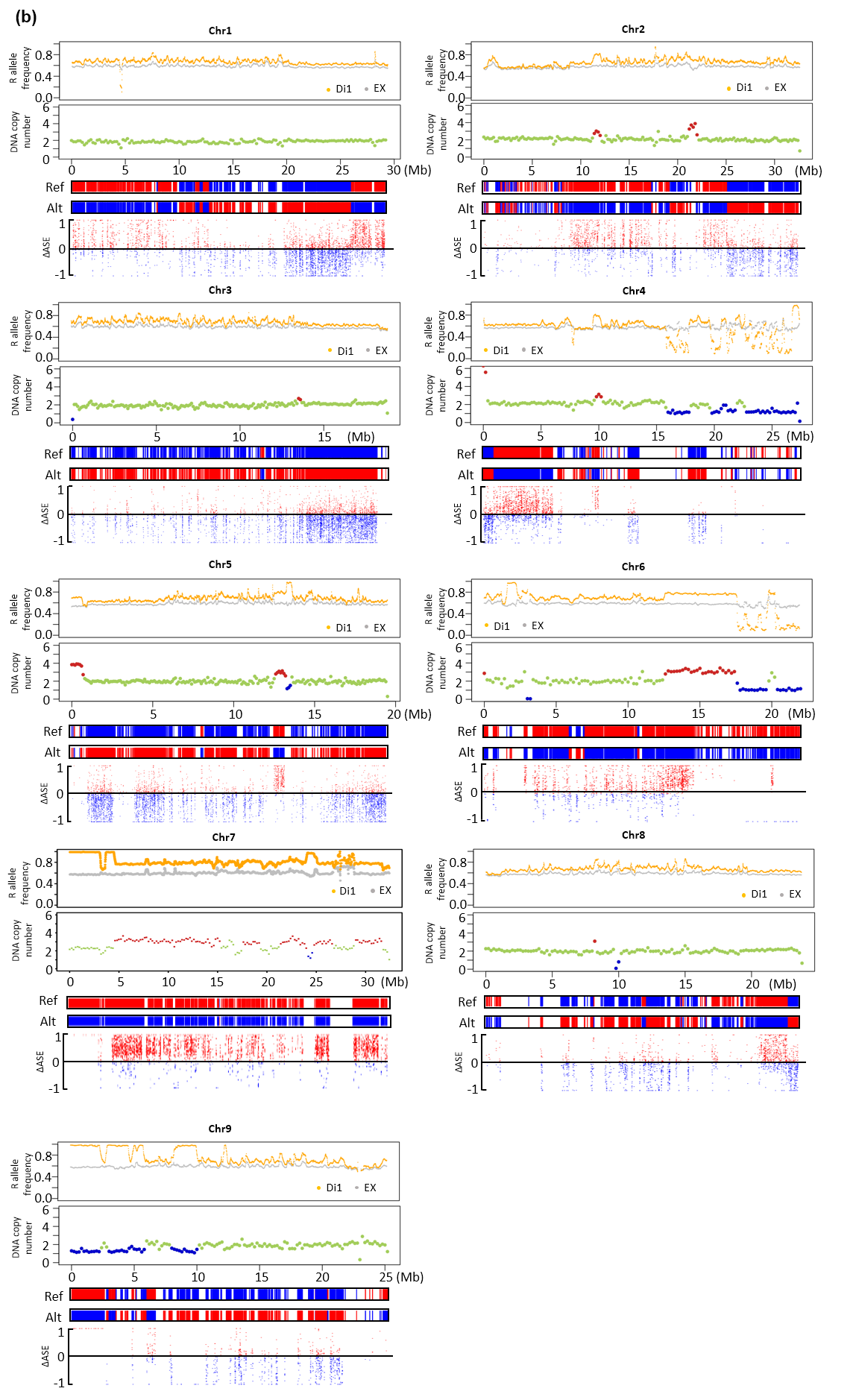


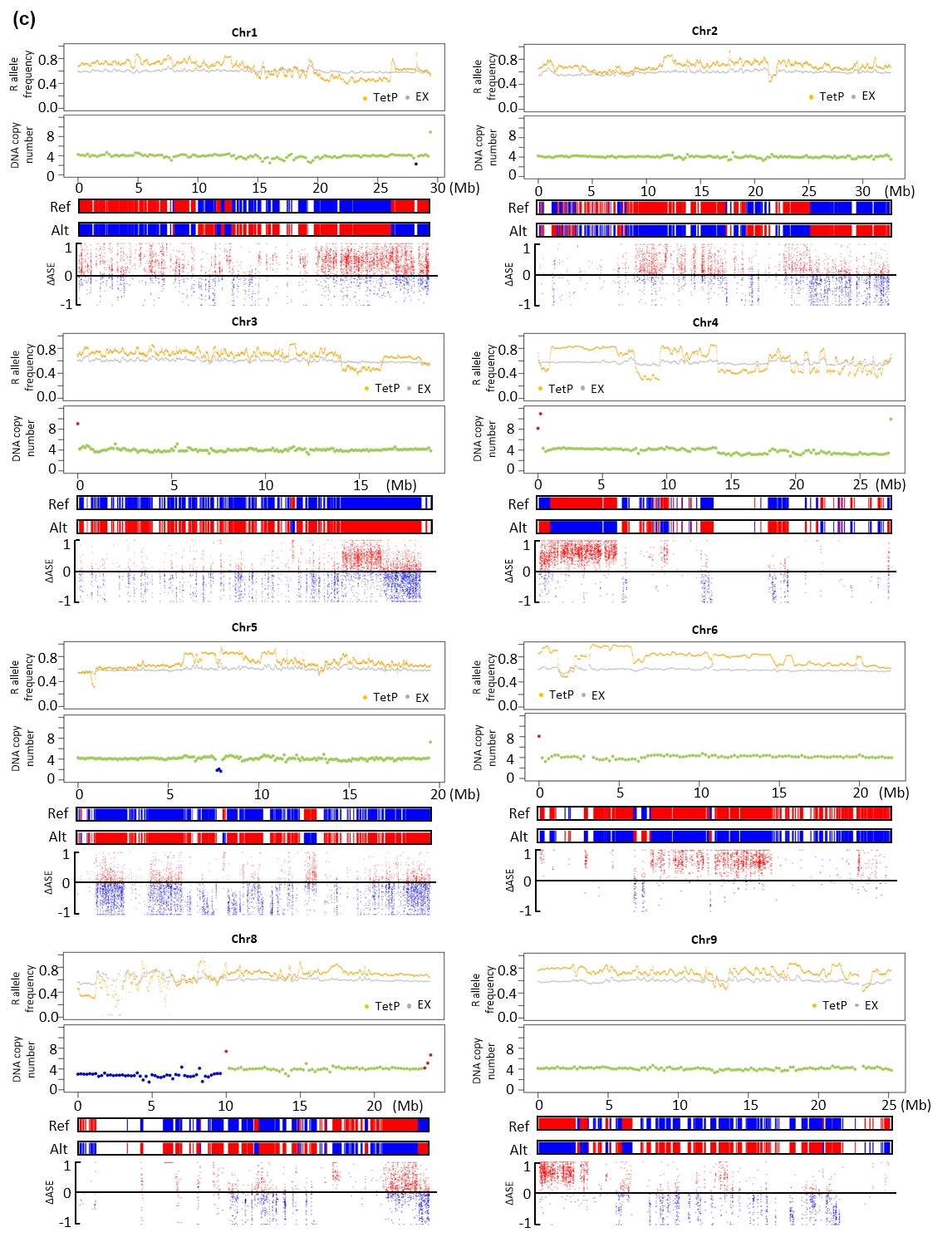

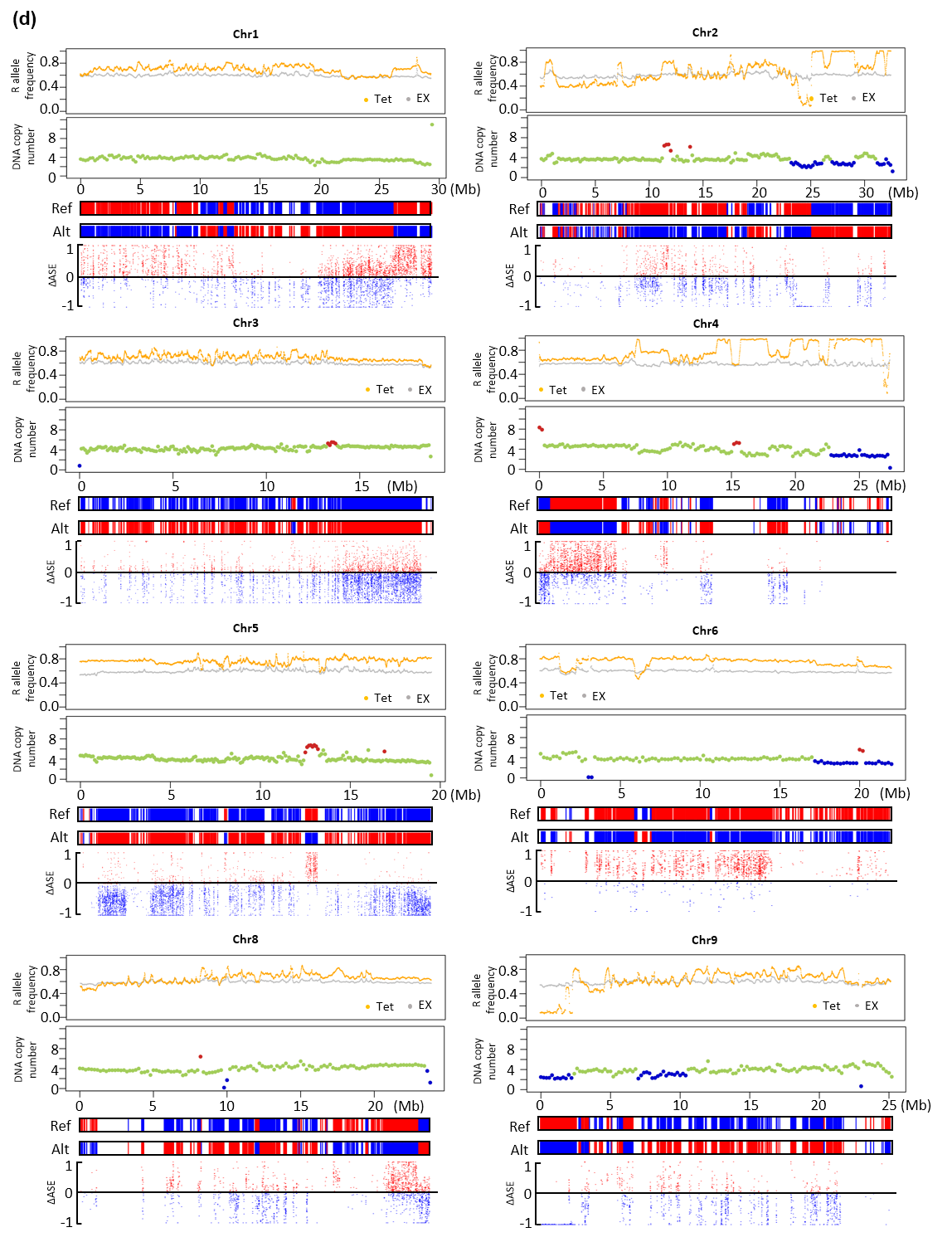


**Fig. S7 Allele-specific copy number variations and allele-specific expression patterns in newly-induced callus (a), diploid callus from long-term culture (b), tetraploid sweet orange plant (c), and tetraploid callus from long-term culture (d).** Top panels, R allele frequency in the test sample (orange) compared with the diploid plant (grey) and the total DNA copy number in the test sample, R allele is the allele on the reference haplotype of the haploid genome of sweet orange (Ref), the frequency of which is a measurement of the relative copy number of the ref haplotype and the alternate haplotype-genome (Alt); Middle panels, the constitution of mandarin (red) and pummelo (blue) in the Ref haplotype and Alt haplotype in sweet orange; Bottom panels, difference in expression levels of alleles of mandarin to pummelo origin (ΔASE) in the test sample. Red indicates mandarin-origin dominant expression, while blue indicates pummelo-origin dominant expression. The patterns on chromosome 7 in tetraploid callus and tetraploid plant are displayed in the Figure 5 in the maintext.


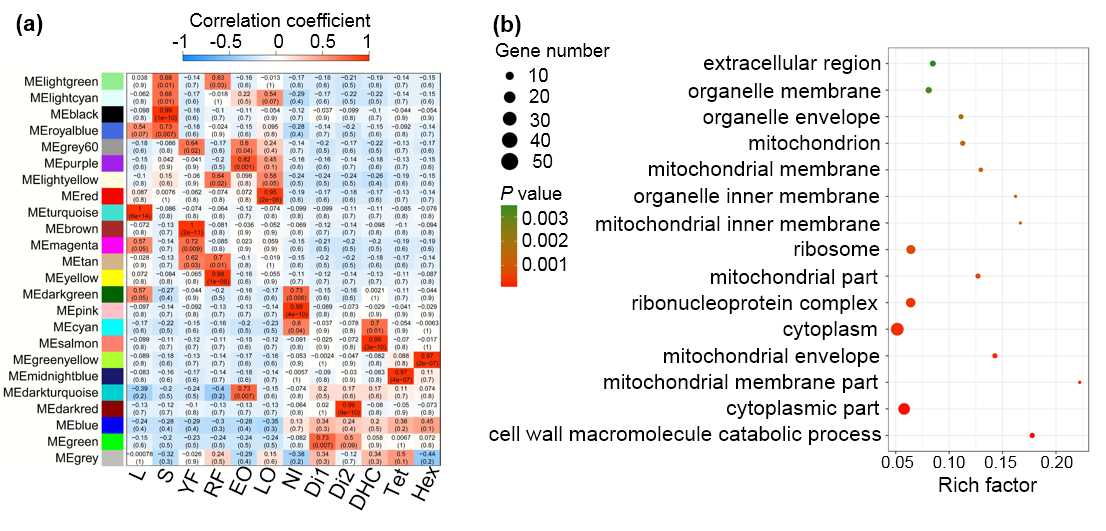


**Fig. S8 Gene co-expression networks for *in vitro* cultured calli and different tissues of sweet orange.** **a** Module-tissue association matrix which classifies the genes expressed in 12 tissues into 24 co-expression modules. The correlation coefficient (in the top row) and its significant *P* value (in parentheses) between each module and tissue were indicated in the matrix. L, leaf; S, seed; YF, young fruit; RF, ripe fruit; EO, early-stage ovule; LO, late-stage ovule; NI, newly induced callus, Di1 and Di2, two independent diploid calli from long-time culture; DHC, Tet, and Hex, double-haploid callus, tetraploid callus, and hexaploid callus from long-time culture. **b** GO enrichments for the genes specifically expressed in the newly induced calli (MEpink module).
