# Supplementary material for: Fruit transpiration drives interspecific variability in fruit growth strategies
Source: Hortic Res. 2022 Feb 19;9:uhac036. doi: 10.1093/hr/uhac036 (PMC8987619; doi:10.1093/hr/uhac036)
Supplement: Web_Material_uhac036 [file web_material_uhac036.zip › Supplementary Tables.docx]

| **Table S1. DNA-seq data from the Illumina platform used for genome analysis.** | | | | | | | | | |
| --- | --- | --- | --- | --- | --- | --- | --- | --- | --- |
| **Library** | **Ploidy** | **Number of read pairs** | **Total bases (bp)** | **Sequencing depth** | **Read length (bp)** | **GC content (%)** | **Data file submitted to NCBI** | | |
|  |  |  |  |  |  |  | **Bioproject** | **Biosample** | **SRR_id** |
| EX1 | 2 | 110146357 | 19826344260 | 49.57 | 90 | 37 | Previously published | | |
| EX2 | 2 | 67647101 | 20294130300 | 50.74 | 150 | 41 | PRJNA573566 | SAMN12817593 | SRR15685571 |
| NI1 | 2 | 67609648 | 20282894400 | 50.71 | 150 | 40 | PRJNA573566 | SAMN12817594 | SRR10236264 |
| NI2 | 2 | 66868225 | 20060467500 | 50.15 | 150 | 39 | PRJNA573566 | SAMN12817595 | SRR10236261 |
| Di1 | 2 | 67307324 | 20192197200 | 50.48 | 150 | 41.5 | PRJNA573566 | SAMN12817596 | SRR10236260 |
| Di2 | 2 | 58420219 | 17526065700 | 43.82 | 150 | 40 | PRJNA573566 | SAMN12817597 | SRR10236259 |
| Tet | 4 | 107606046 | 32281813800 | 40.35 | 150 | 41 | PRJNA573566 | SAMN12817598 | SRR10236258 |
| TetP | 4 | 79411213 | 23823363900 | 29.78 | 150 | 37 | PRJNA573566 | SAMN16427104 | SRR12823230 |
| Hex | 6 | 161061669 | 48318500700 | 40.27 | 150 | 39.5 | PRJNA573566 | SAMN12817599 | SRR10236257 |

| **Table S2. DNA-seq data from third-generation sequencing platform used for genome analysis.** | | | | | | | | | | | | | |
| --- | --- | --- | --- | --- | --- | --- | --- | --- | --- | --- | --- | --- | --- |
| **Library** | **Ploidy** | **Platform** | **Total bases (bp)** | **Sequencing depth** | **Number of subreads** | **Max length of subread (bp)** | **Mean length of subread (bp)** | **Median length of subread (bp)** | **N50 of subread length (bp)** | **GC content (%)** | **Data file submitted to NCBI** | | |
|  |  |  |  |  |  |  |  |  |  |  | **Bioproject** | **Biosample** | **SRR_id** |
| EX | 2 | PacBio | 12094366154 | 30.24 | 1755522 | 71944 | 6889.33 | 5736 | 10824 | 39.38 | PRJNA573566 | SAMN12817593 | SRR10236256 |
| NI | 2 | PacBio | 7573335365 | 18.93 | 879015 | 81920 | 8615.71 | 7326 | 13277 | 39.91 | PRJNA573566 | SAMN12817594 | SRR10236255 |
| Di1 | 2 | PacBio | 5248493402 | 13.12 | 797577 | 65075 | 6580.55 | 5689 | 9771 | 41.59 | PRJNA573566 | SAMN12817596 | SRR10236254 |
| Tet | 4 | PacBio | 10880710272 | 13.60 | 1805906 | 71009 | 6025.07 | 4835 | 10081 | 40.77 | PRJNA573566 | SAMN12817598 | SRR10236263 |
| Hex | 6 | PacBio | 14975118856 | 12.48 | 1944306 | 76168 | 7702.04 | 6627 | 11989 | 39.24 | PRJNA573566 | SAMN12817599 | SRR10236262 |
| TetP | 4 | Nanopore | 37450799004 | 46.81 | 1845085 | 216590 | 20297.60 | 21086 | 25626 | 37.14 | PRJNA573566 | SAMN16427104 | SRR12823229 |

| **Table S3. Whole genome bisulfite sequencing (WGBS) data used for DNA mathylation analysis.** | | | | | | | | |
| --- | --- | --- | --- | --- | --- | --- | --- | --- |
| **Library** | **Ploidy** | **Number of read pairs** | **Total bases (bp)** | **Sequencing depth** | **GC content (%)** | **Data file submitted to NCBI** | | |
|  |  |  |  |  |  | **Bioproject** | **Biosample** | **SRR_id** |
| L-1 | 2 | 65755891 | 19726767300 | 49.32 | 23 | PRJNA573590 | SAMN12817593 | SRR10187286 |
| L-2 | 2 | 55678830 | 16703649000 | 41.76 | 22 | PRJNA573590 | SAMN12817593 | SRR10187285 |
| L-3 | 2 | 60237844 | 18071353200 | 45.18 | 24 | PRJNA573590 | SAMN12817593 | SRR10187276 |
| EX-1 | 2 | 50055086 | 14986192679 | 37.47 | 28 | PRJNA573590 | SAMN21168115 | SRR15685570 |
| EX-2 | 2 | 76458539 | 22881307231 | 57.20 | 29 | PRJNA573590 | SAMN21168115 | SRR15685569 |
| NI-1 | 2 | 73139307 | 21941792100 | 54.85 | 23 | PRJNA573590 | SAMN12817594 | SRR10187275 |
| NI-2 | 2 | 59339824 | 17801947200 | 44.50 | 23 | PRJNA573590 | SAMN12817594 | SRR10187274 |
| NI-3 | 2 | 60754842 | 18226452600 | 45.57 | 23 | PRJNA573590 | SAMN12817594 | SRR10187273 |
| Di1-1 | 2 | 47355065 | 14206519500 | 35.52 | 24 | PRJNA573590 | SAMN12817596 | SRR10187272 |
| Di1-2 | 2 | 62220784 | 18666235200 | 46.67 | 23 | PRJNA573590 | SAMN12817596 | SRR10187271 |
| Di1-3 | 2 | 51066228 | 15319868400 | 38.30 | 23 | PRJNA573590 | SAMN12817596 | SRR10187270 |
| Di2-1 | 2 | 55823173 | 16746951900 | 41.87 | 23.5 | PRJNA573590 | SAMN12817597 | SRR10187269 |
| Di2-2 | 2 | 44786694 | 13436008200 | 33.59 | 23 | PRJNA573590 | SAMN12817597 | SRR10187284 |
| Di2-3 | 2 | 55367103 | 16610130900 | 41.53 | 23 | PRJNA573590 | SAMN12817597 | SRR10187283 |
| Tet-1 | 4 | 93911121 | 28173336300 | 35.22 | 23 | PRJNA573590 | SAMN12817598 | SRR10187282 |
| Tet-2 | 4 | 117738069 | 35321420700 | 44.15 | 23 | PRJNA573590 | SAMN12817598 | SRR10187281 |
| Tet-3 | 4 | 85659397 | 25697819100 | 32.12 | 23 | PRJNA573590 | SAMN12817598 | SRR10187280 |
| TetP-1 | 4 | 132511026 | 40018329852 | 50.02 | 21 | PRJNA573590 | SAMN16427104 | SRR12822514 |
| TetP-2 | 4 | 128915027 | 38932338154 | 48.67 | 21 | PRJNA573590 | SAMN16427104 | SRR12822513 |
| TetP-3 | 4 | 114590172 | 34606231944 | 43.26 | 21 | PRJNA573590 | SAMN16427104 | SRR12822512 |
| Hex-1 | 6 | 170390014 | 51117004200 | 42.60 | 23 | PRJNA573590 | SAMN12817599 | SRR10187279 |
| Hex-2 | 6 | 119002812 | 35700843600 | 29.75 | 25 | PRJNA573590 | SAMN12817599 | SRR10187278 |
| Hex-3 | 6 | 132533075 | 39759922500 | 33.13 | 24.5 | PRJNA573590 | SAMN12817599 | SRR10187277 |

| **Table S4. RNA-seq data used for transcriptome analysis.** | | | | | | | | | |
| --- | --- | --- | --- | --- | --- | --- | --- | --- | --- |
| **Library** | **Tissue** | **Number of read pairs** | **Total bases (bp)** | **Read length  (bp)** | **GC content (%)** | **Data file submitted to NCBI** | | | **Whether sequenced in this study** |
|  |  |  |  |  |  | **Bioproject** | **Biosample** | **SRR_id** |  |
| L-1 | Leaf of diploid plant | 37355488 | 11206646400 | 150 | 44 | PRJNA573594 | SAMN12817593 | SRR10193326 | Yes |
| L-2 | Leaf of diploid plant | 31677536 | 9503260800 | 150 | 44 | PRJNA573594 | SAMN12817593 | SRR10193325 | Yes |
| L-3 | Leaf of diploid plant | 29758497 | 8927549100 | 150 | 44 | PRJNA573594 | SAMN12817593 | SRR10193314 | Yes |
| NI-1 | Newly-induced callus | 26803773 | 8041131900 | 150 | 44 | PRJNA573594 | SAMN12817594 | SRR10193312 | Yes |
| NI-2 | Newly-induced callus | 32945810 | 9883743000 | 150 | 44 | PRJNA573594 | SAMN12817594 | SRR10193311 | Yes |
| NI-3 | Newly-induced callus | 34322579 | 10296773700 | 150 | 44 | PRJNA573594 | SAMN12817594 | SRR10193310 | Yes |
| Di1-1 | Diploid callus | 38055968 | 11416790400 | 150 | 44 | PRJNA573594 | SAMN12817596 | SRR10193309 | Yes |
| Di1-2 | Diploid callus | 33654677 | 10096403100 | 150 | 43.5 | PRJNA573594 | SAMN12817596 | SRR10193308 | Yes |
| Di1-3 | Diploid callus | 34604206 | 10381261800 | 150 | 44 | PRJNA573594 | SAMN12817596 | SRR10193307 | Yes |
| Di2-1 | Diploid callus | 31526288 | 9457886400 | 150 | 44 | PRJNA573594 | SAMN12817597 | SRR10193306 | Yes |
| Di2-2 | Diploid callus | 41080425 | 12324127500 | 150 | 44 | PRJNA573594 | SAMN12817597 | SRR10193324 | Yes |
| Di2-3 | Diploid callus | 45807106 | 13742131800 | 150 | 44 | PRJNA573594 | SAMN12817597 | SRR10193323 | Yes |
| Tet-1 | Tetraploid callus | 34902228 | 10470668400 | 150 | 44 | PRJNA573594 | SAMN12817598 | SRR10193322 | Yes |
| Tet-2 | Tetraploid callus | 38873415 | 11662024500 | 150 | 44 | PRJNA573594 | SAMN12817598 | SRR10193321 | Yes |
| Tet-3 | Tetraploid callus | 38534475 | 11560342500 | 150 | 44 | PRJNA573594 | SAMN12817598 | SRR10193320 | Yes |
| TetP-1 | Leaf of tetraploid plant | 33079465 | 9923839500 | 150 | 44 | PRJNA573594 | SAMN16427104 | SRR12822753 | Yes |
| TetP-2 | Leaf of tetraploid plant | 41501471 | 12450441300 | 150 | 44 | PRJNA573594 | SAMN16427104 | SRR12822752 | Yes |
| TetP-3 | Leaf of tetraploid plant | 37579095 | 11273728500 | 150 | 44 | PRJNA573594 | SAMN16427104 | SRR12822751 | Yes |
| Hex-1 | Hexaploid callus | 28225581 | 8467674300 | 150 | 44 | PRJNA573594 | SAMN12817599 | SRR10193319 | Yes |
| Hex-2 | Hexaploid callus | 36517870 | 10955361000 | 150 | 44 | PRJNA573594 | SAMN12817599 | SRR10193318 | Yes |
| Hex-3 | Hexaploid callus | 31363997 | 9409199100 | 150 | 44 | PRJNA573594 | SAMN12817599 | SRR10193317 | Yes |
| DHC-1 | DH callus | 26764949 | 8029484700 | 150 | 44 | PRJNA573594 | SAMN12837791 | SRR10193316 | Yes |
| DHC-2 | DH callus | 34661647 | 10398494100 | 150 | 44 | PRJNA573594 | SAMN12837791 | SRR10193315 | Yes |
| DHC-3 | DH callus | 37774138 | 11332241400 | 150 | 44 | PRJNA573594 | SAMN12837791 | SRR10193313 | Yes |
| S_1 | Seed | 21581453 | 6474435900 | 125 | 45 | - | - | - | No |
| S_2 | Seed | 20748477 | 6224543100 | 125 | 45 | - | - | - | No |
| YF_1 | Young fruit | 23419011 | 7025703300 | 125 | 45 | - | - | - | No |
| YF_2 | Young fruit | 22017288 | 6605186400 | 125 | 45 | - | - | - | No |
| RF_1 | Ripe fruit | 21002569 | 6300770700 | 125 | 43 | - | - | - | No |
| RF_2 | Ripe fruit | 20386679 | 6116003700 | 125 | 44 | - | - | - | No |
| EO_1 | Early-stage ovule | 21485610 | 6445683000 | 125 | 44 | - | - | - | No |
| EO_2 | Early-stage ovule | 24958839 | 7487651700 | 125 | 44 | - | - | - | No |
| LO_1 | Late-stage ovule | 21996655 | 6598996500 | 125 | 43 | - | - | - | No |
| LO_2 | Late-stage ovule | 26192297 | 7857689100 | 125 | 43 | - | - | - | No |

| **Table S5. The detected TE insertions in the nascent calli.** | | | | | |
| --- | --- | --- | --- | --- | --- |
| **TE integration loci** | **Length of inserted sequence (bp)** | **Type of the pre-TE** | **Genomic coordinate of the pre-TE** | **Identity between the inserted sequence and the pre-TE sequences (%)** | **Specific in nascent calli or shared by nascent and old calli** |
| chr7:13406186 | 200 | DNA | chr3:1749720-1749910 | 89.41 | Specific in nascent calli |
| chr4:13764103 | 200 | DNA | chr5:3270307-3270496 | 97.37 | Specific in nascent calli |
| chr2:21525727 | 183 | DNA | chr5:38265607-38265799 | 95.63 | Specific in nascent calli |
| chr9:3713984 | 97 | DNA | chr9:4496717-4496843 | 93.48 | Specific in nascent calli |
| chr2:13098519 | 201 | DNA | chrUn:6353054-6353246 | 89.94 | Specific in nascent calli |
| chr3:36187670 | 205 | DNA | chrUn:6353054-6353246 | 91.58 | Specific in nascent calli |
| chr4:18142772 | 201 | DNA | chrUn:6353054-6353246 | 91.58 | Shared by nascent and old calli |
| chr5:5473736 | 201 | DNA | chrUn:6353054-6353246 | 91.02 | Specific in nascent calli |
| chr8:14607603 | 198 | DNA | chrUn:10889246-10889436 | 93.37 | Shared by nascent and old calli |
| chr3:28622367 | 198 | DNA | chrUn:12669230-12669422 | 91.58 | Specific in nascent calli |
| chr4:6624143 | 82 | DNA | chrUn:12669230-12669422 | 95.89 | Specific in nascent calli |
| chr6:17218980 | 363 | DNA/hat | chr1:14635556-14635901 | 96.54 | Shared by nascent and old calli |
| chr2:30571595 | 319 | DNA/hat | chr1:25558503-25558816 | 95.86 | Specific in nascent calli |
| chr3:36566533 | 320 | DNA/hat | chr1:25558503-25558816 | 95.30 | Specific in nascent calli |
| chr4:17380309 | 322 | DNA/hat | chr1:25558503-25558816 | 95.82 | Specific in nascent calli |
| chr4:26130004 | 323 | DNA/hat | chr1:25558503-25558816 | 94.90 | Shared by nascent and old calli |
| chr4:26635732 | 312 | DNA/hat | chr1:25558503-25558816 | 95.83 | Specific in nascent calli |
| chr5:2479884 | 330 | DNA/hat | chr1:25558503-25558816 | 95.54 | Specific in nascent calli |
| chr5:10219103 | 328 | DNA/hat | chr1:25558503-25558816 | 96.17 | Specific in nascent calli |
| chr5:36571425 | 325 | DNA/hat | chr1:25558503-25558816 | 95.51 | Specific in nascent calli |
| chr7:5303427 | 320 | DNA/hat | chr1:25558503-25558816 | 96.33 | Specific in nascent calli |
| chr8:18432144 | 327 | DNA/hat | chr1:25558503-25558816 | 94.59 | Specific in nascent calli |
| chr9:1256643 | 322 | DNA/hat | chr1:25558503-25558816 | 95.85 | Specific in nascent calli |
| chr9:13073836 | 326 | DNA/hat | chr1:25558503-25558816 | 94.59 | Specific in nascent calli |
| chr9:22662492 | 319 | DNA/hat | chr1:25558503-25558816 | 95.19 | Specific in nascent calli |
| chr3:16053615 | 345 | DNA/hat | chr1:28630136-28630470 | 98.20 | Specific in nascent calli |
| chr5:5871671 | 354 | DNA/hat | chr1:28630136-28630470 | 98.80 | Specific in nascent calli |
| chr5:35364136 | 335 | DNA/hat | chr1:28630136-28630470 | 98.48 | Specific in nascent calli |
| chr2:26658963 | 354 | DNA/hat | chr2:3113926-3114264 | 99.10 | Specific in nascent calli |
| chr7:30928896 | 213 | DNA/hat | chr2:3113926-3114264 | 100.00 | Specific in nascent calli |
| chr1:4012959 | 346 | DNA/hat | chr2:3259240-3259574 | 98.81 | Specific in nascent calli |
| chr1:4417584 | 350 | DNA/hat | chr2:3259240-3259574 | 98.79 | Specific in nascent calli |
| chr2:5558774 | 351 | DNA/hat | chr2:3259240-3259574 | 98.80 | Specific in nascent calli |
| chr3:4274065 | 349 | DNA/hat | chr2:3259240-3259574 | 97.90 | Specific in nascent calli |
| chr4:2179989 | 358 | DNA/hat | chr2:3259240-3259574 | 99.10 | Shared by nascent and old calli |
| chr4:10469016 | 350 | DNA/hat | chr2:3259240-3259574 | 98.66 | Specific in nascent calli |
| chr5:7375164 | 354 | DNA/hat | chr2:3259240-3259574 | 99.10 | Specific in nascent calli |
| chr9:3560601 | 335 | DNA/hat | chr2:3259240-3259574 | 93.25 | Specific in nascent calli |
| chr1:4260277 | 358 | DNA/hat | chr2:31454573-31454912 | 99.41 | Specific in nascent calli |
| chr1:18724580 | 349 | DNA/hat | chr2:31454573-31454912 | 97.61 | Specific in nascent calli |
| chr3:16256153 | 349 | DNA/hat | chr2:31454573-31454912 | 99.41 | Specific in nascent calli |
| chr5:5360718 | 352 | DNA/hat | chr2:31454573-31454912 | 99.41 | Specific in nascent calli |
| chr7:28824859 | 356 | DNA/hat | chr2:31454573-31454912 | 99.10 | Specific in nascent calli |
| chr8:21204035 | 346 | DNA/hat | chr2:31454573-31454912 | 99.11 | Specific in nascent calli |
| chr3:13676109 | 336 | DNA/hat | chr2:32319598-32319917 | 97.46 | Specific in nascent calli |
| chr7:27379064 | 311 | DNA/hat | chr2:32319598-32319917 | 96.73 | Specific in nascent calli |
| chr7:2596106 | 355 | DNA/hat | chr2:32483531-32483868 | 98.53 | Specific in nascent calli |
| chr7:3864441 | 281 | DNA/hat | chr2:32483531-32483868 | 94.40 | Specific in nascent calli |
| chr4:1724043 | 428 | DNA/hat | chr3:18915636-18915955 | 93.44 | Specific in nascent calli |
| chr7:6261119 | 409 | DNA/hat | chr3:20206379-20206686 | 94.79 | Specific in nascent calli |
| chr6:4650504 | 334 | DNA/hat | chr3:24211511-24211837 | 93.50 | Specific in nascent calli |
| chr2:29694348 | 311 | DNA/hat | chr3:27812516-27812815 | 95.33 | Shared by nascent and old calli |
| chr8:6217198 | 314 | DNA/hat | chr3:29467697-29468005 | 96.89 | Specific in nascent calli |
| chr9:1872 | 681 | DNA/hat | chr3:33264167-33264831 | 95.23 | Specific in nascent calli |
| chr3:29604172 | 332 | DNA/hat | chr3:33707429-33707749 | 93.31 | Specific in nascent calli |
| chr2:25274221 | 374 | DNA/hat | chr3:36508274-36508625 | 97.73 | Specific in nascent calli |
| chr3:36268486 | 114 | DNA/hat | chr3:36508274-36508625 | 99.09 | Specific in nascent calli |
| chr7:8272084 | 377 | DNA/hat | chr3:36508274-36508625 | 95.77 | Specific in nascent calli |
| chr9:1072875 | 359 | DNA/hat | chr3:36508274-36508625 | 98.25 | Specific in nascent calli |
| chr8:5041034 | 67 | DNA/hat | chr4:9626661-9626838 | 96.83 | Specific in nascent calli |
| chr3:30657897 | 255 | DNA/hat | chr4:26860182-26860450 | 99.18 | Specific in nascent calli |
| chr6:2842008 | 217 | DNA/hat | chr4:26860182-26860450 | 100.00 | Specific in nascent calli |
| chr9:19954392 | 289 | DNA/hat | chr4:26860182-26860450 | 99.25 | Specific in nascent calli |
| chr4:11598644 | 233 | DNA/hat | chr5:11202740-11203039 | 95.26 | Specific in nascent calli |
| chr2:371715 | 353 | DNA/hat | chr5:14809407-14809738 | 98.48 | Specific in nascent calli |
| chr5:4890491 | 345 | DNA/hat | chr5:14809407-14809738 | 98.78 | Specific in nascent calli |
| chr7:2360054 | 342 | DNA/hat | chr5:14809407-14809738 | 93.66 | Specific in nascent calli |
| chr9:13398589 | 365 | DNA/hat | chr5:14809407-14809738 | 98.80 | Specific in nascent calli |
| chr5:34028467 | 324 | DNA/hat | chr5:23281144-23281454 | 95.51 | Specific in nascent calli |
| chr3:12351041 | 258 | DNA/hat | chr5:28087626-28087939 | 98.30 | Specific in nascent calli |
| chr4:1215879 | 323 | DNA/hat | chr5:28087626-28087939 | 98.38 | Specific in nascent calli |
| chr4:3999521 | 329 | DNA/hat | chr5:28087626-28087939 | 98.41 | Specific in nascent calli |
| chr4:10411335 | 317 | DNA/hat | chr5:28087626-28087939 | 99.21 | Specific in nascent calli |
| chr4:16649500 | 370 | DNA/hat | chr5:31053504-31053851 | 97.70 | Specific in nascent calli |
| chr8:21767285 | 53 | DNA/hat | chr5:32331875-32332055 | 96.15 | Shared by nascent and old calli |
| chr7:17761300 | 349 | DNA/hat | chr5:34252471-34252807 | 94.63 | Specific in nascent calli |
| chr9:22045325 | 342 | DNA/hat | chr5:34252471-34252807 | 95.21 | Specific in nascent calli |
| chr8:20086860 | 293 | DNA/hat | chr6:15987975-15988242 | 99.62 | Specific in nascent calli |
| chr7:6235161 | 321 | DNA/hat | chr7:30668584-30668899 | 83.44 | Shared by nascent and old calli |
| chr8:593833 | 148 | DNA/hat | chr8:593666-593974 | 99.55 | Shared by nascent and old calli |
| chr1:28857723 | 326 | DNA/hat | chr8:2126956-2127275 | 94.27 | Specific in nascent calli |
| chr2:27616836 | 325 | DNA/hat | chr8:2126956-2127275 | 94.23 | Specific in nascent calli |
| chr4:21183340 | 328 | DNA/hat | chr8:2126956-2127275 | 94.67 | Shared by nascent and old calli |
| chr4:22757681 | 327 | DNA/hat | chr8:2126956-2127275 | 93.65 | Specific in nascent calli |
| chr8:7507197 | 336 | DNA/hat | chr8:2126956-2127275 | 93.77 | Specific in nascent calli |
| chr8:12804420 | 312 | DNA/hat | chr8:2126956-2127275 | 87.70 | Specific in nascent calli |
| chr9:1671007 | 337 | DNA/hat | chr8:2152024-2152356 | 96.34 | Specific in nascent calli |
| chr1:2083401 | 284 | DNA/hat | chr8:5520677-5520943 | 93.51 | Specific in nascent calli |
| chr4:8507641 | 257 | DNA/hat | chr8:15353806-15354070 | 98.73 | Specific in nascent calli |
| chr4:8686796 | 318 | DNA/hat | chr8:23598317-23598633 | 92.01 | Specific in nascent calli |
| chr3:30466655 | 325 | DNA/hat | chr9:14061748-14062086 | 96.62 | Specific in nascent calli |
| chr4:24863314 | 353 | DNA/hat | chr9:14061748-14062086 | 99.41 | Specific in nascent calli |
| chr5:6578049 | 351 | DNA/hat | chr9:14061748-14062086 | 95.27 | Specific in nascent calli |
| chr5:37215893 | 361 | DNA/hat | chr9:14061748-14062086 | 98.82 | Specific in nascent calli |
| chr2:6594988 | 75 | DNA/hat | chrUn:5232478-5232730 | 100.00 | Specific in nascent calli |
| chr8:14575363 | 53 | DNA/hat | chrUn:5456314-5456575 | 100.00 | Specific in nascent calli |
| chr8:5272911 | 321 | DNA/hat | chrUn:12139035-12139353 | 95.28 | Specific in nascent calli |
| chr1:3873221 | 321 | DNA/hat | chrUn:12471944-12472254 | 99.52 | Shared by nascent and old calli |
| chr2:30410156 | 317 | DNA/hat | chrUn:12471944-12472254 | 99.36 | Specific in nascent calli |
| chr4:19960864 | 326 | DNA/hat | chrUn:12471944-12472254 | 99.35 | Shared by nascent and old calli |
| chr5:3625264 | 295 | DNA/hat | chrUn:12471944-12472254 | 99.32 | Specific in nascent calli |
| chr5:12345347 | 316 | DNA/hat | chrUn:12471944-12472254 | 99.36 | Specific in nascent calli |
| chr7:5223462 | 390 | DNA/hat | chrUn:12471944-12472254 | 98.91 | Specific in nascent calli |
| chr8:1260847 | 328 | DNA/hat | chrUn:12471944-12472254 | 99.36 | Specific in nascent calli |
| chr8:12701830 | 319 | DNA/hat | chrUn:12471944-12472254 | 99.02 | Specific in nascent calli |
| chr4:1213197 | 368 | DNA/hat | chrUn:51458372-51458713 | 92.75 | Specific in nascent calli |
| chr2:8696985 | 191 | DNA/hat | chrUn:51634863-51635176 | 100.00 | Specific in nascent calli |
| chr1:23449971 | 188 | DNA/hat | chrUn:57677243-57677582 | 100.00 | Specific in nascent calli |
| chr4:5894137 | 337 | DNA/hat | chrUn:57677243-57677582 | 99.39 | Specific in nascent calli |
| chr2:19775627 | 350 | DNA/hat | chrUn:60793494-60793826 | 99.10 | Specific in nascent calli |
| chr7:3303521 | 347 | DNA/hat | chrUn:60793494-60793826 | 97.90 | Shared by nascent and old calli |
| chr1:13113200 | 293 | DNA/hat | chrUn:65257601-65257912 | 96.14 | Specific in nascent calli |
| chr6:8717057 | 585 | DNA/hAT-Ac | chr1:20172584-20173136 | 94.97 | Shared by nascent and old calli |
| chr2:25329595 | 977 | DNA/hAT-Ac | chr1:23493616-23494704 | 97.81 | Specific in nascent calli |
| chr9:2739855 | 883 | DNA/hAT-Ac | chr2:14602318-14603191 | 99.31 | Shared by nascent and old calli |
| chr9:11364561 | 884 | DNA/hAT-Ac | chr2:14602318-14603191 | 99.31 | Specific in nascent calli |
| chr2:6212883 | 868 | DNA/hAT-Ac | chr2:26845269-26846126 | 98.02 | Specific in nascent calli |
| chr5:9372496 | 870 | DNA/hAT-Ac | chr3:12119816-12120757 | 94.33 | Specific in nascent calli |
| chr8:20869951 | 858 | DNA/hAT-Ac | chr3:12409635-12410481 | 97.63 | Specific in nascent calli |
| chr4:8245238 | 966 | DNA/hAT-Ac | chr3:12690818-12691814 | 93.44 | Shared by nascent and old calli |
| chr7:19689963 | 994 | DNA/hAT-Ac | chr3:12690818-12691814 | 99.69 | Specific in nascent calli |
| chr3:34893408 | 61 | DNA/hAT-Ac | chr3:34893316-34893872 | 98.39 | Specific in nascent calli |
| chr3:28691303 | 961 | DNA/hAT-Ac | chr4:12315438-12316399 | 90.15 | Shared by nascent and old calli |
| chr3:28873473 | 997 | DNA/hAT-Ac | chr4:13052991-13053981 | 97.18 | Specific in nascent calli |
| chr3:30773623 | 993 | DNA/hAT-Ac | chr4:19510264-19511240 | 95.27 | Specific in nascent calli |
| chr3:33426144 | 992 | DNA/hAT-Ac | chr4:19510264-19511240 | 96.25 | Specific in nascent calli |
| chr1:6697225 | 875 | DNA/hAT-Ac | chr4:20023078-20023943 | 98.50 | Shared by nascent and old calli |
| chr2:27677226 | 972 | DNA/hAT-Ac | chr5:213083-214046 | 90.25 | Specific in nascent calli |
| chr8:15805868 | 877 | DNA/hAT-Ac | chr5:8615164-8616030 | 98.85 | Specific in nascent calli |
| chr5:9795623 | 65 | DNA/hAT-Ac | chr5:9795516-9795637 | 95.24 | Specific in nascent calli |
| chr4:26322429 | 562 | DNA/hAT-Ac | chr5:12100238-12100798 | 94.25 | Specific in nascent calli |
| chr3:8229714 | 868 | DNA/hAT-Ac | chr5:14968710-14969583 | 98.58 | Shared by nascent and old calli |
| chr8:3447182 | 883 | DNA/hAT-Ac | chr5:14968710-14969583 | 96.80 | Specific in nascent calli |
| chr8:11060070 | 885 | DNA/hAT-Ac | chr5:14968710-14969583 | 97.83 | Specific in nascent calli |
| chr1:5678166 | 161 | DNA/hAT-Ac | chr5:23584857-23588361 | 94.08 | Specific in nascent calli |
| chr1:24245998 | 161 | DNA/hAT-Ac | chr5:23584857-23588361 | 94.08 | Specific in nascent calli |
| chr1:24619573 | 154 | DNA/hAT-Ac | chr5:23584857-23588361 | 94.74 | Specific in nascent calli |
| chr1:27941149 | 161 | DNA/hAT-Ac | chr5:23584857-23588361 | 94.74 | Specific in nascent calli |
| chr1:28985171 | 160 | DNA/hAT-Ac | chr5:23584857-23588361 | 94.81 | Specific in nascent calli |
| chr2:3011736 | 162 | DNA/hAT-Ac | chr5:23584857-23588361 | 94.74 | Specific in nascent calli |
| chr2:8569576 | 160 | DNA/hAT-Ac | chr5:23584857-23588361 | 94.74 | Specific in nascent calli |
| chr2:11635026 | 162 | DNA/hAT-Ac | chr5:23584857-23588361 | 94.08 | Specific in nascent calli |
| chr3:13644968 | 162 | DNA/hAT-Ac | chr5:23584857-23588361 | 94.19 | Specific in nascent calli |
| chr3:21246665 | 161 | DNA/hAT-Ac | chr5:23584857-23588361 | 95.39 | Specific in nascent calli |
| chr3:32430534 | 132 | DNA/hAT-Ac | chr5:23584857-23588361 | 93.94 | Specific in nascent calli |
| chr3:34689220 | 161 | DNA/hAT-Ac | chr5:23584857-23588361 | 94.74 | Specific in nascent calli |
| chr4:2757926 | 148 | DNA/hAT-Ac | chr5:23584857-23588361 | 94.56 | Specific in nascent calli |
| chr4:4702356 | 163 | DNA/hAT-Ac | chr5:23584857-23588361 | 94.12 | Shared by nascent and old calli |
| chr4:7342794 | 161 | DNA/hAT-Ac | chr5:23584857-23588361 | 94.81 | Specific in nascent calli |
| chr4:7932431 | 160 | DNA/hAT-Ac | chr5:23584857-23588361 | 96.43 | Specific in nascent calli |
| chr4:8162240 | 161 | DNA/hAT-Ac | chr5:23584857-23588361 | 94.30 | Specific in nascent calli |
| chr4:8502798 | 161 | DNA/hAT-Ac | chr5:23584857-23588361 | 94.38 | Specific in nascent calli |
| chr4:17136163 | 162 | DNA/hAT-Ac | chr5:23584857-23588361 | 94.74 | Specific in nascent calli |
| chr4:19417052 | 162 | DNA/hAT-Ac | chr5:23584857-23588361 | 95.39 | Specific in nascent calli |
| chr4:23181730 | 161 | DNA/hAT-Ac | chr5:23584857-23588361 | 94.12 | Specific in nascent calli |
| chr4:24361050 | 161 | DNA/hAT-Ac | chr5:23584857-23588361 | 94.12 | Specific in nascent calli |
| chr5:1227771 | 160 | DNA/hAT-Ac | chr5:23584857-23588361 | 94.70 | Specific in nascent calli |
| chr5:1495528 | 162 | DNA/hAT-Ac | chr5:23584857-23588361 | 94.74 | Specific in nascent calli |
| chr5:3278101 | 161 | DNA/hAT-Ac | chr5:23584857-23588361 | 93.42 | Specific in nascent calli |
| chr5:4510952 | 142 | DNA/hAT-Ac | chr5:23584857-23588361 | 96.12 | Specific in nascent calli |
| chr5:6228711 | 161 | DNA/hAT-Ac | chr5:23584857-23588361 | 94.27 | Specific in nascent calli |
| chr5:7880528 | 159 | DNA/hAT-Ac | chr5:23584857-23588361 | 94.74 | Specific in nascent calli |
| chr5:20158848 | 135 | DNA/hAT-Ac | chr5:23584857-23588361 | 94.57 | Specific in nascent calli |
| chr5:31574966 | 161 | DNA/hAT-Ac | chr5:23584857-23588361 | 93.75 | Specific in nascent calli |
| chr5:34522314 | 158 | DNA/hAT-Ac | chr5:23584857-23588361 | 94.74 | Specific in nascent calli |
| chr6:16511326 | 161 | DNA/hAT-Ac | chr5:23584857-23588361 | 94.12 | Specific in nascent calli |
| chr7:3916739 | 155 | DNA/hAT-Ac | chr5:23584857-23588361 | 95.42 | Specific in nascent calli |
| chr7:6478138 | 161 | DNA/hAT-Ac | chr5:23584857-23588361 | 94.74 | Specific in nascent calli |
| chr7:24650665 | 161 | DNA/hAT-Ac | chr5:23584857-23588361 | 94.74 | Specific in nascent calli |
| chr7:25202783 | 161 | DNA/hAT-Ac | chr5:23584857-23588361 | 94.08 | Specific in nascent calli |
| chr7:26333952 | 155 | DNA/hAT-Ac | chr5:23584857-23588361 | 94.08 | Specific in nascent calli |
| chr8:4251530 | 157 | DNA/hAT-Ac | chr5:23584857-23588361 | 94.74 | Specific in nascent calli |
| chr8:6715437 | 162 | DNA/hAT-Ac | chr5:23584857-23588361 | 94.74 | Specific in nascent calli |
| chr8:10799589 | 149 | DNA/hAT-Ac | chr5:23584857-23588361 | 93.92 | Specific in nascent calli |
| chr8:18877796 | 153 | DNA/hAT-Ac | chr5:23584857-23588361 | 94.74 | Specific in nascent calli |
| chr8:22435156 | 161 | DNA/hAT-Ac | chr5:23584857-23588361 | 93.42 | Specific in nascent calli |
| chr9:3825924 | 161 | DNA/hAT-Ac | chr5:23584857-23588361 | 94.74 | Specific in nascent calli |
| chr9:5740253 | 160 | DNA/hAT-Ac | chr5:23584857-23588361 | 93.46 | Specific in nascent calli |
| chr9:17044853 | 139 | DNA/hAT-Ac | chr5:23584857-23588361 | 94.49 | Specific in nascent calli |
| chr2:10327717 | 993 | DNA/hAT-Ac | chr5:35783887-35784882 | 92.61 | Specific in nascent calli |
| chr5:36341120 | 997 | DNA/hAT-Ac | chr5:35783887-35784882 | 94.03 | Specific in nascent calli |
| chr5:546931 | 951 | DNA/hAT-Ac | chr5:37934017-37934977 | 93.21 | Specific in nascent calli |
| chr6:3753315 | 104 | DNA/hAT-Ac | chr6:3753219-3753373 | 85.96 | Specific in nascent calli |
| chr7:29085935 | 67 | DNA/hAT-Ac | chr6:15586283-15586846 | 89.39 | Specific in nascent calli |
| chr9:22115965 | 976 | DNA/hAT-Ac | chr6:16607948-16608922 | 95.90 | Specific in nascent calli |
| chr5:4983199 | 994 | DNA/hAT-Ac | chr7:12215654-12216661 | 96.99 | Specific in nascent calli |
| chr7:21064359 | 149 | DNA/hAT-Ac | chr7:21063606-21065117 | 93.96 | Specific in nascent calli |
| chr4:19913793 | 75 | DNA/hAT-Ac | chr7:22125187-22127508 | 100.00 | Specific in nascent calli |
| chr6:1993707 | 93 | DNA/hAT-Ac | chr7:29894575-29894866 | 82.76 | Specific in nascent calli |
| chr1:4669354 | 842 | DNA/hAT-Ac | chr7:32419517-32420348 | 93.90 | Specific in nascent calli |
| chr3:11399269 | 110 | DNA/hAT-Ac | chr8:687436-687613 | 93.01 | Shared by nascent and old calli |
| chr3:25272313 | 84 | DNA/hAT-Ac | chr8:4112990-4113571 | 89.89 | Specific in nascent calli |
| chr1:4261480 | 89 | DNA/hAT-Ac | chr8:11070940-11074525 | 95.45 | Shared by nascent and old calli |
| chr1:8166946 | 151 | DNA/hAT-Ac | chr8:11070940-11074525 | 95.07 | Shared by nascent and old calli |
| chr5:23920 | 152 | DNA/hAT-Ac | chr8:11070940-11074525 | 95.07 | Specific in nascent calli |
| chr6:10237922 | 150 | DNA/hAT-Ac | chr8:11070940-11074525 | 94.33 | Specific in nascent calli |
| chr9:19463156 | 141 | DNA/hAT-Ac | chr8:11070940-11074525 | 95.04 | Specific in nascent calli |
| chr9:3810554 | 864 | DNA/hAT-Ac | chr8:16141344-16142185 | 99.41 | Specific in nascent calli |
| chr8:17671098 | 66 | DNA/hAT-Ac | chr8:17670903-17671241 | 100.00 | Specific in nascent calli |
| chr2:23237337 | 423 | DNA/hAT-Ac | chr9:9223495-9223893 | 93.13 | Specific in nascent calli |
| chr2:23902253 | 421 | DNA/hAT-Ac | chr9:9223495-9223893 | 92.00 | Shared by nascent and old calli |
| chr5:5757594 | 862 | DNA/hAT-Ac | chr9:11234853-11235727 | 97.35 | Specific in nascent calli |
| chr9:7371431 | 877 | DNA/hAT-Ac | chr9:11234853-11235727 | 97.83 | Specific in nascent calli |
| chr4:19884260 | 995 | DNA/hAT-Ac | chr9:19076698-19077691 | 97.34 | Specific in nascent calli |
| chr9:23805968 | 991 | DNA/hAT-Ac | chr9:19076698-19077691 | 94.86 | Specific in nascent calli |
| chr7:10972758 | 77 | DNA/hAT-Ac | chr9:19577651-19578574 | 100.00 | Specific in nascent calli |
| chr9:12637921 | 100 | DNA/hAT-Ac | chrUn:12176988-12177169 | 92.27 | Specific in nascent calli |
| chr7:12299306 | 958 | DNA/hAT-Ac | chrUn:41732936-41733902 | 97.08 | Specific in nascent calli |
| chr3:20892680 | 78 | DNA/hAT-Ac | chrUn:51471995-51472285 | 97.44 | Shared by nascent and old calli |
| chr2:6264359 | 993 | DNA/hAT-Ac | chrUn:55617636-55618629 | 91.27 | Specific in nascent calli |
| chr3:28408117 | 995 | DNA/hAT-Ac | chrUn:55617636-55618629 | 91.94 | Specific in nascent calli |
| chr4:18817894 | 980 | DNA/hAT-Ac | chrUn:55617636-55618629 | 92.83 | Specific in nascent calli |
| chr5:38191578 | 990 | DNA/hAT-Ac | chrUn:55617636-55618629 | 91.99 | Shared by nascent and old calli |
| chr9:11511432 | 969 | DNA/hAT-Ac | chrUn:55617636-55618629 | 92.29 | Shared by nascent and old calli |
| chr1:684084 | 885 | DNA/hAT-Ac | chrUn:58751226-58752100 | 99.31 | Specific in nascent calli |
| chr4:21573637 | 784 | DNA/hAT-Ac | chrUn:58751226-58752100 | 98.64 | Specific in nascent calli |
| chr4:23665376 | 883 | DNA/hAT-Ac | chrUn:67079526-67080399 | 95.19 | Specific in nascent calli |
| chr8:4763181 | 72 | DNA/hAT-Ac | chrUn:67304400-67307412 | 97.83 | Specific in nascent calli |
| chr2:24508160 | 67 | DNA/hAT-Tip100 | chr9:11832452-11834116 | 98.51 | Shared by nascent and old calli |
| chr3:36913454 | 608 | DNA/MULE-MuDR | chr1:20939612-20940290 | 85.61 | Specific in nascent calli |
| chr9:16855698 | 54 | DNA/MuLE-MuDR | chr2:18785604-18786094 | 94.55 | Shared by nascent and old calli |
| chr1:4317948 | 689 | DNA/MULE-MuDR | chr4:4710605-4711335 | 90.33 | Shared by nascent and old calli |
| chr1:14959788 | 706 | DNA/MULE-MuDR | chr4:4710605-4711335 | 89.56 | Shared by nascent and old calli |
| chr7:8092176 | 479 | DNA/MULE-MuDR | chr4:14286702-14287270 | 95.17 | Shared by nascent and old calli |
| chr4:9543933 | 629 | DNA/MULE-MuDR | chr4:18358875-18359517 | 90.19 | Specific in nascent calli |
| chr1:18701768 | 652 | DNA/MULE-MuDR | chr5:11257267-11257812 | 88.90 | Shared by nascent and old calli |
| chr2:7044987 | 547 | DNA/MULE-MuDR | chr6:613256-613797 | 88.44 | Specific in nascent calli |
| chr7:6937864 | 552 | DNA/MULE-MuDR | chr6:613256-613797 | 89.22 | Specific in nascent calli |
| chr6:5921223 | 609 | DNA/MULE-MuDR | chr6:18628834-18629445 | 97.32 | Specific in nascent calli |
| chr9:21916480 | 98 | DNA/MULE-MuDR | chr7:18298395-18299032 | 94.06 | Shared by nascent and old calli |
| chr7:29895150 | 815 | DNA/MULE-MuDR | chr7:22007797-22008551 | 97.72 | Specific in nascent calli |
| chr5:2209210 | 652 | DNA/MULE-MuDR | chr8:18327216-18327870 | 97.48 | Shared by nascent and old calli |
| chr2:21662998 | 529 | DNA/MULE-MuDR | chr9:13932900-13933435 | 91.39 | Specific in nascent calli |
| chr9:20245734 | 540 | DNA/MULE-MuDR | chr9:13932900-13933435 | 91.41 | Specific in nascent calli |
| chr4:23498111 | 549 | DNA/MULE-MuDR | chrUn:7045520-7046063 | 93.76 | Specific in nascent calli |
| chr1:6530866 | 184 | DNA/PIF-Harbinger | chr1:17851264-17851680 | 100.00 | Specific in nascent calli |
| chr6:4719105 | 709 | DNA/PIF-Harbinger | chr1:19003846-19004564 | 99.58 | Specific in nascent calli |
| chr6:4758327 | 709 | DNA/PIF-Harbinger | chr1:19003846-19004564 | 99.44 | Specific in nascent calli |
| chr8:19943175 | 709 | DNA/PIF-Harbinger | chr1:19003846-19004564 | 99.72 | Specific in nascent calli |
| chr9:18186823 | 708 | DNA/PIF-Harbinger | chr4:17614963-17615679 | 99.15 | Specific in nascent calli |
| chr2:6293012 | 696 | DNA/PIF-Harbinger | chr7:10250532-10251237 | 98.15 | Shared by nascent and old calli |
| chr4:27190555 | 704 | DNA/PIF-Harbinger | chr7:10250532-10251237 | 98.72 | Specific in nascent calli |
| chr3:24413122 | 57 | DNA/PIF-Harbinger | chr9:19089941-19090002 | 100.00 | Specific in nascent calli |
| chr8:21555983 | 711 | DNA/PIF-Harbinger | chr9:19969876-19970594 | 99.01 | Specific in nascent calli |
| chr1:15818233 | 662 | DNA/TcMar-Pogo | chr9:4809134-4809791 | 89.56 | Specific in nascent calli |
| chr5:14127245 | 93 | LINE/L1 | chr1:2673070-2674026 | 95.70 | Specific in nascent calli |
| chr9:23654438 | 231 | LINE/L1 | chr1:16775575-16776664 | 86.45 | Specific in nascent calli |
| chr5:6303994 | 229 | LINE/L1 | chr1:29026823-29032404 | 98.10 | Specific in nascent calli |
| chr5:25606895 | 982 | LINE/L1 | chr2:23853823-23855223 | 97.54 | Specific in nascent calli |
| chr1:29071007 | 369 | LINE/L1 | chr2:28277808-28278369 | 99.18 | Specific in nascent calli |
| chr4:27147835 | 150 | LINE/L1 | chr3:5445655-5448161 | 100.00 | Specific in nascent calli |
| chr3:6624139 | 99 | LINE/L1 | chr3:6487881-6492394 | 92.38 | Shared by nascent and old calli |
| chr7:1948154 | 287 | LINE/L1 | chr3:23579634-23580032 | 99.24 | Specific in nascent calli |
| chr4:25472912 | 331 | LINE/L1 | chr4:5337663-5338217 | 95.83 | Specific in nascent calli |
| chr5:7263367 | 104 | LINE/L1 | chr4:10656535-10657679 | 85.71 | Specific in nascent calli |
| chr7:27436011 | 197 | LINE/L1 | chr5:20027964-20028362 | 96.09 | Specific in nascent calli |
| chr9:1023575 | 160 | LINE/L1 | chr6:13788861-13789416 | 92.47 | Specific in nascent calli |
| chr5:38909817 | 352 | LINE/L1 | chr6:15570347-15570769 | 96.58 | Specific in nascent calli |
| chr8:14810688 | 384 | LINE/L1 | chr7:4595743-4599948 | 92.99 | Specific in nascent calli |
| chr4:26516160 | 387 | LINE/L1 | chr7:14289642-14290187 | 98.03 | Specific in nascent calli |
| chr3:27232040 | 164 | LINE/L1 | chrUn:5984997-5985158 | 95.60 | Shared by nascent and old calli |
| chr5:26755429 | 161 | LINE/L1 | chrUn:30063419-30063604 | 90.20 | Specific in nascent calli |
| chr8:4622903 | 58 | LINE/L1 | chrUn:45825133-45827539 | 100.00 | Specific in nascent calli |
| chr6:18821249 | 414 | LINE/L1 | chrUn:58189872-58195717 | 96.80 | Shared by nascent and old calli |
| chr4:3906248 | 251 | LINE/L1 | chrUn:64423210-64424635 | 99.20 | Specific in nascent calli |
| chr3:30930520 | 729 | LINE/L2 | chr1:28736381-28737104 | 95.96 | Specific in nascent calli |
| chr5:10176734 | 740 | LINE/L2 | chr1:28736381-28737104 | 94.82 | Specific in nascent calli |
| chr7:13782790 | 736 | LINE/L2 | chr1:28736381-28737104 | 95.36 | Specific in nascent calli |
| chr7:30276177 | 737 | LINE/L2 | chr1:28736381-28737104 | 96.03 | Specific in nascent calli |
| chr4:6808556 | 702 | LINE/L2 | chr2:5037384-5038103 | 96.88 | Specific in nascent calli |
| chr8:675160 | 739 | LINE/L2 | chr2:16368317-16369042 | 94.67 | Specific in nascent calli |
| chr2:16900317 | 660 | LINE/L2 | chr2:17287972-17288646 | 95.38 | Shared by nascent and old calli |
| chr2:26774250 | 758 | LINE/L2 | chr3:30428668-30429423 | 95.00 | Specific in nascent calli |
| chr5:10388300 | 754 | LINE/L2 | chr3:30428668-30429423 | 92.68 | Specific in nascent calli |
| chr4:1738390 | 753 | LINE/L2 | chr5:29919457-29920181 | 94.88 | Specific in nascent calli |
| chr4:9023094 | 741 | LINE/L2 | chr5:29919457-29920181 | 96.72 | Specific in nascent calli |
| chr7:7595937 | 729 | LINE/L2 | chr5:29919457-29920181 | 97.67 | Shared by nascent and old calli |
| chr1:8216710 | 712 | LINE/L2 | chr5:36791354-36792062 | 95.93 | Specific in nascent calli |
| chr4:6910725 | 707 | LINE/L2 | chr5:36791354-36792062 | 98.31 | Specific in nascent calli |
| chr8:16629439 | 713 | LINE/L2 | chr5:36791354-36792062 | 98.59 | Specific in nascent calli |
| chr4:6042850 | 736 | LINE/L2 | chr6:4723748-4724466 | 96.39 | Specific in nascent calli |
| chr4:7345795 | 740 | LINE/L2 | chr6:4723748-4724466 | 95.39 | Specific in nascent calli |
| chr6:8464207 | 731 | LINE/L2 | chr6:4723748-4724466 | 96.94 | Specific in nascent calli |
| chr1:21011707 | 726 | LINE/L2 | chr9:8834429-8835144 | 98.60 | Shared by nascent and old calli |
| chr4:26784842 | 702 | LINE/L2 | chr9:8834429-8835144 | 98.95 | Specific in nascent calli |
| chr5:26706914 | 751 | LINE/L2 | chrUn:29926545-29927248 | 89.56 | Specific in nascent calli |
| chr8:21682529 | 741 | LINE/L2 | chrUn:29926545-29927248 | 96.08 | Shared by nascent and old calli |
| chr2:10228725 | 703 | LINE/L2 | chrUn:63988949-63989664 | 98.35 | Shared by nascent and old calli |
| chr5:4192511 | 724 | LINE/L2 | chrUn:63988949-63989664 | 98.18 | Shared by nascent and old calli |
| chr8:19494392 | 724 | LINE/L2 | chrUn:63988949-63989664 | 97.50 | Specific in nascent calli |
| chr2:3166595 | 675 | LTR | chr7:22183482-22184497 | 98.48 | Shared by nascent and old calli |
| chr3:4458258 | 372 | LTR | chr8:7605378-7605745 | 98.87 | Specific in nascent calli |
| chr4:26322125 | 136 | LTR | chrUn:51706715-51707082 | 96.97 | Specific in nascent calli |
| chr3:11218679 | 54 | LTR/Caulimovirus | chr1:7917418-7922561 | 100.00 | Specific in nascent calli |
| chr5:2886982 | 225 | LTR/Caulimovirus | chr3:9174660-9178620 | 98.22 | Specific in nascent calli |
| chr3:2269813 | 129 | LTR/Caulimovirus | chr3:11064140-11069009 | 100.00 | Specific in nascent calli |
| chr3:15506985 | 93 | LTR/Caulimovirus | chr3:15503045-15510208 | 100.00 | Specific in nascent calli |
| chr5:23691261 | 148 | LTR/Caulimovirus | chr5:23691041-23691846 | 97.28 | Specific in nascent calli |
| chr9:23869542 | 217 | LTR/Caulimovirus | chr5:24987255-24994362 | 98.47 | Specific in nascent calli |
| chr8:11842945 | 99 | LTR/Caulimovirus | chr7:20015830-20016181 | 100.00 | Specific in nascent calli |
| chr7:16048618 | 90 | LTR/Caulimovirus | chrUn:53377719-53381631 | 100.00 | Specific in nascent calli |
| chr7:15767267 | 69 | LTR/Caulimovirus | chrUn:57378204-57385431 | 98.55 | Specific in nascent calli |
| chr5:4276564 | 194 | LTR/Copia | chr1:1884814-1885000 | 99.47 | Specific in nascent calli |
| chr3:4401713 | 206 | LTR/Copia | chr1:2908614-2908814 | 90.45 | Shared by nascent and old calli |
| chr4:940101 | 387 | LTR/Copia | chr1:11156442-11157335 | 100.00 | Specific in nascent calli |
| chr6:12421550 | 890 | LTR/Copia | chr1:11156442-11157335 | 99.55 | Specific in nascent calli |
| chr2:21584603 | 575 | LTR/Copia | chr1:11220318-11221096 | 94.77 | Specific in nascent calli |
| chr1:7273671 | 53 | LTR/Copia | chr1:13796526-13798205 | 96.23 | Shared by nascent and old calli |
| chr7:30784994 | 359 | LTR/Copia | chr1:19292174-19292540 | 98.89 | Specific in nascent calli |
| chr3:22326999 | 210 | LTR/Copia | chr1:20065207-20065408 | 90.83 | Specific in nascent calli |
| chr6:13280894 | 576 | LTR/Copia | chr1:21853240-21858210 | 99.47 | Shared by nascent and old calli |
| chr6:8924979 | 208 | LTR/Copia | chr1:23619971-23620172 | 86.67 | Specific in nascent calli |
| chr7:13271214 | 190 | LTR/Copia | chr1:24345137-24345323 | 97.24 | Specific in nascent calli |
| chr4:8459348 | 349 | LTR/Copia | chr1:25695620-25695957 | 97.34 | Specific in nascent calli |
| chr5:4162235 | 372 | LTR/Copia | chr1:25695620-25695957 | 95.34 | Specific in nascent calli |
| chr1:26940864 | 198 | LTR/Copia | chr1:26940590-26940914 | 98.94 | Specific in nascent calli |
| chr9:11685578 | 661 | LTR/Copia | chr1:28504118-28504777 | 98.18 | Specific in nascent calli |
| chr4:4286004 | 876 | LTR/Copia | chr2:2433641-2434525 | 98.86 | Specific in nascent calli |
| chr9:2437465 | 196 | LTR/Copia | chr2:3789754-3789940 | 97.30 | Specific in nascent calli |
| chr5:61918 | 209 | LTR/Copia | chr2:4091426-4091627 | 96.04 | Specific in nascent calli |
| chr5:3886540 | 211 | LTR/Copia | chr2:4091426-4091627 | 91.38 | Specific in nascent calli |
| chr9:2346693 | 210 | LTR/Copia | chr2:4091426-4091627 | 92.33 | Specific in nascent calli |
| chr1:20515221 | 502 | LTR/Copia | chr2:4110056-4110417 | 99.13 | Specific in nascent calli |
| chr5:34201220 | 363 | LTR/Copia | chr2:4110056-4110417 | 99.72 | Specific in nascent calli |
| chr8:3146519 | 363 | LTR/Copia | chr2:4110056-4110417 | 98.89 | Specific in nascent calli |
| chr8:8051803 | 363 | LTR/Copia | chr2:4110056-4110417 | 98.60 | Specific in nascent calli |
| chr9:24064199 | 363 | LTR/Copia | chr2:4110056-4110417 | 98.61 | Specific in nascent calli |
| chr8:19143387 | 211 | LTR/Copia | chr2:6564567-6564771 | 90.45 | Specific in nascent calli |
| chr2:32443340 | 364 | LTR/Copia | chr2:6844615-6844981 | 98.90 | Specific in nascent calli |
| chr1:13066899 | 718 | LTR/Copia | chr2:8028759-8029489 | 89.97 | Specific in nascent calli |
| chr2:26906653 | 707 | LTR/Copia | chr2:9305349-9306046 | 95.11 | Specific in nascent calli |
| chr5:10664391 | 650 | LTR/Copia | chr2:9305349-9306046 | 97.15 | Specific in nascent calli |
| chr8:19173320 | 680 | LTR/Copia | chr2:10190525-10191139 | 99.19 | Specific in nascent calli |
| chr4:15996463 | 879 | LTR/Copia | chr2:10810012-10810917 | 94.18 | Specific in nascent calli |
| chr9:12555816 | 154 | LTR/Copia | chr2:12918030-12918215 | 85.16 | Shared by nascent and old calli |
| chr2:13649187 | 203 | LTR/Copia | chr2:13648695-13649485 | 98.12 | Specific in nascent calli |
| chr9:4077325 | 210 | LTR/Copia | chr2:17730617-17730820 | 90.10 | Specific in nascent calli |
| chr4:16742732 | 98 | LTR/Copia | chr2:17834750-17844717 | 96.43 | Specific in nascent calli |
| chr2:17884428 | 147 | LTR/Copia | chr2:17887845-17890164 | 98.45 | Specific in nascent calli |
| chr7:6963041 | 846 | LTR/Copia | chr2:19182090-19182935 | 98.68 | Specific in nascent calli |
| chr2:20424373 | 71 | LTR/Copia | chr2:20415558-20416008 | 100.00 | Specific in nascent calli |
| chr2:22521018 | 126 | LTR/Copia | chr2:22519700-22521227 | 92.37 | Specific in nascent calli |
| chr9:13400018 | 424 | LTR/Copia | chr2:22809695-22810113 | 86.71 | Shared by nascent and old calli |
| chr5:23562037 | 426 | LTR/Copia | chr2:26223432-26223849 | 94.93 | Specific in nascent calli |
| chr8:4103062 | 425 | LTR/Copia | chr2:26223432-26223849 | 98.06 | Specific in nascent calli |
| chr4:20689057 | 439 | LTR/Copia | chr2:27704475-27704930 | 91.14 | Specific in nascent calli |
| chr3:12215921 | 822 | LTR/Copia | chr2:27905942-27906774 | 99.04 | Specific in nascent calli |
| chr5:2280919 | 213 | LTR/Copia | chr3:7429252-7429453 | 91.09 | Specific in nascent calli |
| chr9:2534067 | 58 | LTR/Copia | chr3:10470161-10473215 | 98.28 | Shared by nascent and old calli |
| chr5:10378519 | 210 | LTR/Copia | chr3:11626016-11626220 | 99.51 | Specific in nascent calli |
| chr4:14207420 | 225 | LTR/Copia | chr3:13576035-13576251 | 94.04 | Specific in nascent calli |
| chr1:11460574 | 211 | LTR/Copia | chr3:13899647-13899848 | 99.50 | Specific in nascent calli |
| chr2:22907169 | 211 | LTR/Copia | chr3:13899647-13899848 | 99.01 | Shared by nascent and old calli |
| chr2:18763622 | 418 | LTR/Copia | chr3:17810150-17810568 | 93.17 | Shared by nascent and old calli |
| chr3:35021806 | 426 | LTR/Copia | chr3:17810150-17810568 | 93.35 | Specific in nascent calli |
| chr6:1840329 | 336 | LTR/Copia | chr3:18043415-18044706 | 82.69 | Shared by nascent and old calli |
| chr1:4526676 | 819 | LTR/Copia | chr3:22109696-22110511 | 98.29 | Specific in nascent calli |
| chr2:24862147 | 788 | LTR/Copia | chr3:22109696-22110511 | 98.28 | Specific in nascent calli |
| chr7:9381825 | 863 | LTR/Copia | chr3:22109696-22110511 | 98.90 | Shared by nascent and old calli |
| chr2:21576191 | 211 | LTR/Copia | chr3:23680705-23680909 | 89.51 | Specific in nascent calli |
| chr3:33202205 | 692 | LTR/Copia | chr3:24064163-24064855 | 98.95 | Specific in nascent calli |
| chr5:28255156 | 692 | LTR/Copia | chr3:24064163-24064855 | 98.99 | Specific in nascent calli |
| chr2:17056750 | 862 | LTR/Copia | chr3:24622399-24623269 | 99.30 | Specific in nascent calli |
| chr6:18165273 | 878 | LTR/Copia | chr3:24749743-24750610 | 97.16 | Specific in nascent calli |
| chr2:27764224 | 363 | LTR/Copia | chr3:24940433-24940798 | 99.72 | Shared by nascent and old calli |
| chr4:4833124 | 364 | LTR/Copia | chr3:24940433-24940798 | 99.44 | Specific in nascent calli |
| chr5:26192517 | 700 | LTR/Copia | chr3:25620886-25621580 | 98.71 | Specific in nascent calli |
| chr6:9845800 | 705 | LTR/Copia | chr3:25620886-25621580 | 98.85 | Specific in nascent calli |
| chr9:20381591 | 702 | LTR/Copia | chr3:25620886-25621580 | 98.85 | Shared by nascent and old calli |
| chr4:1396260 | 168 | LTR/Copia | chr3:25783558-25783759 | 90.76 | Specific in nascent calli |
| chr6:17408308 | 220 | LTR/Copia | chr3:26455927-26456128 | 90.40 | Shared by nascent and old calli |
| chr4:7995698 | 62 | LTR/Copia | chr3:29048579-29049250 | 92.31 | Specific in nascent calli |
| chr8:2846474 | 842 | LTR/Copia | chr3:30492871-30493743 | 96.13 | Specific in nascent calli |
| chr4:3156864 | 364 | LTR/Copia | chr3:31242167-31242533 | 98.90 | Specific in nascent calli |
| chr3:34724112 | 95 | LTR/Copia | chr3:34724336-34729145 | 100.00 | Specific in nascent calli |
| chr9:12938722 | 348 | LTR/Copia | chr4:6950743-6955556 | 95.71 | Specific in nascent calli |
| chr1:4201086 | 661 | LTR/Copia | chr4:8523852-8524446 | 97.32 | Specific in nascent calli |
| chr8:16449430 | 873 | LTR/Copia | chr4:10141483-10142362 | 96.70 | Specific in nascent calli |
| chr1:3319727 | 213 | LTR/Copia | chr4:12449481-12449684 | 89.03 | Specific in nascent calli |
| chr6:3780113 | 210 | LTR/Copia | chr4:12449481-12449684 | 88.92 | Specific in nascent calli |
| chr5:27745369 | 207 | LTR/Copia | chr4:15713071-15713272 | 97.99 | Specific in nascent calli |
| chr2:19152632 | 805 | LTR/Copia | chr4:19152800-19153612 | 98.00 | Specific in nascent calli |
| chr4:25418929 | 141 | LTR/Copia | chr4:25433884-25434232 | 91.24 | Specific in nascent calli |
| chr8:2344310 | 471 | LTR/Copia | chr4:25893470-25894118 | 94.20 | Shared by nascent and old calli |
| chr2:6640571 | 226 | LTR/Copia | chr5:2480771-2480987 | 92.57 | Specific in nascent calli |
| chr5:12670431 | 51 | LTR/Copia | chr5:11314025-11317336 | 100.00 | Specific in nascent calli |
| chr5:12334358 | 184 | LTR/Copia | chr5:12332782-12334333 | 93.41 | Specific in nascent calli |
| chr7:11982324 | 447 | LTR/Copia | chr5:12566696-12567151 | 95.16 | Specific in nascent calli |
| chr4:24040604 | 186 | LTR/Copia | chr5:15338024-15338491 | 91.67 | Specific in nascent calli |
| chr9:1693874 | 362 | LTR/Copia | chr5:17136054-17136414 | 98.88 | Specific in nascent calli |
| chr6:11811383 | 81 | LTR/Copia | chr5:17247222-17249376 | 100.00 | Specific in nascent calli |
| chr2:11736195 | 425 | LTR/Copia | chr5:19347692-19348108 | 92.39 | Specific in nascent calli |
| chr4:26081653 | 414 | LTR/Copia | chr5:19347692-19348108 | 92.33 | Specific in nascent calli |
| chr7:16476038 | 424 | LTR/Copia | chr5:19347692-19348108 | 92.47 | Specific in nascent calli |
| chr6:18125250 | 385 | LTR/Copia | chr5:21658030-21658231 | 90.08 | Specific in nascent calli |
| chr4:17895627 | 424 | LTR/Copia | chr5:24285192-24285609 | 92.50 | Specific in nascent calli |
| chr7:27298154 | 530 | LTR/Copia | chr5:24336685-24341774 | 96.57 | Specific in nascent calli |
| chr3:35044856 | 698 | LTR/Copia | chr5:24446391-24447083 | 98.70 | Specific in nascent calli |
| chr4:17049096 | 701 | LTR/Copia | chr5:24446391-24447083 | 98.99 | Specific in nascent calli |
| chr5:2477684 | 697 | LTR/Copia | chr5:24446391-24447083 | 98.99 | Specific in nascent calli |
| chr8:5042450 | 692 | LTR/Copia | chr5:24446391-24447083 | 99.24 | Specific in nascent calli |
| chr8:22182718 | 699 | LTR/Copia | chr5:24446391-24447083 | 99.42 | Specific in nascent calli |
| chr4:11389567 | 372 | LTR/Copia | chr5:25031792-25036345 | 99.18 | Specific in nascent calli |
| chr2:18211727 | 361 | LTR/Copia | chr5:28467881-28468245 | 98.07 | Specific in nascent calli |
| chr2:13603684 | 872 | LTR/Copia | chr5:29570614-29571483 | 98.96 | Specific in nascent calli |
| chr4:21262200 | 871 | LTR/Copia | chr5:29570614-29571483 | 99.31 | Specific in nascent calli |
| chr4:23740640 | 709 | LTR/Copia | chr5:33424284-33424998 | 92.86 | Specific in nascent calli |
| chr3:16346632 | 211 | LTR/Copia | chr5:36469832-36470036 | 89.60 | Specific in nascent calli |
| chr4:16945730 | 211 | LTR/Copia | chr5:36469832-36470036 | 96.98 | Specific in nascent calli |
| chr2:26371337 | 210 | LTR/Copia | chr5:38049175-38049376 | 93.35 | Specific in nascent calli |
| chr3:31306186 | 211 | LTR/Copia | chr5:38049175-38049376 | 93.35 | Shared by nascent and old calli |
| chr3:33771919 | 386 | LTR/Copia | chr5:38049175-38049376 | 89.85 | Specific in nascent calli |
| chr8:8752778 | 211 | LTR/Copia | chr5:38049175-38049376 | 93.35 | Specific in nascent calli |
| chr8:15258013 | 193 | LTR/Copia | chr5:38049175-38049376 | 93.04 | Specific in nascent calli |
| chr6:4819115 | 97 | LTR/Copia | chr6:2676600-2678595 | 98.95 | Specific in nascent calli |
| chr9:4208951 | 350 | LTR/Copia | chr6:6552574-6552919 | 95.60 | Specific in nascent calli |
| chr3:34939277 | 903 | LTR/Copia | chr6:9689944-9690854 | 98.13 | Specific in nascent calli |
| chr9:3623940 | 906 | LTR/Copia | chr6:9689944-9690854 | 98.46 | Specific in nascent calli |
| chr5:29200076 | 458 | LTR/Copia | chr6:10277515-10280251 | 89.01 | Specific in nascent calli |
| chr3:607822 | 211 | LTR/Copia | chr6:11781947-11782150 | 88.74 | Shared by nascent and old calli |
| chr2:23724735 | 333 | LTR/Copia | chr7:2504831-2505181 | 97.43 | Specific in nascent calli |
| chr5:26766060 | 348 | LTR/Copia | chr7:2504831-2505181 | 96.84 | Specific in nascent calli |
| chr4:21174112 | 69 | LTR/Copia | chr7:3320607-3321247 | 93.94 | Specific in nascent calli |
| chr3:1571816 | 364 | LTR/Copia | chr7:13897504-13897912 | 99.16 | Specific in nascent calli |
| chr6:10206990 | 103 | LTR/Copia | chr7:17297731-17299060 | 100.00 | Shared by nascent and old calli |
| chr8:11855814 | 236 | LTR/Copia | chr7:19059154-19060754 | 87.55 | Specific in nascent calli |
| chr5:27590756 | 379 | LTR/Copia | chr7:19998577-19998952 | 96.53 | Specific in nascent calli |
| chr8:10279666 | 374 | LTR/Copia | chr7:19998577-19998952 | 97.86 | Specific in nascent calli |
| chr9:6657241 | 424 | LTR/Copia | chr7:23799405-23799823 | 95.95 | Specific in nascent calli |
| chr9:22035983 | 425 | LTR/Copia | chr7:23799405-23799823 | 95.73 | Specific in nascent calli |
| chr9:15390688 | 361 | LTR/Copia | chr7:26224543-26224908 | 98.61 | Specific in nascent calli |
| chr7:29483443 | 136 | LTR/Copia | chr7:29482361-29483489 | 96.26 | Specific in nascent calli |
| chr1:726876 | 212 | LTR/Copia | chr7:31966209-31966410 | 90.78 | Specific in nascent calli |
| chr2:6595408 | 426 | LTR/Copia | chr8:427180-427597 | 95.64 | Specific in nascent calli |
| chr3:32291060 | 426 | LTR/Copia | chr8:427180-427597 | 94.75 | Specific in nascent calli |
| chr4:22903582 | 422 | LTR/Copia | chr8:427180-427597 | 94.47 | Specific in nascent calli |
| chr5:17953965 | 426 | LTR/Copia | chr8:427180-427597 | 94.44 | Specific in nascent calli |
| chr5:26567110 | 406 | LTR/Copia | chr8:427180-427597 | 94.23 | Specific in nascent calli |
| chr9:15335121 | 423 | LTR/Copia | chr8:427180-427597 | 93.04 | Specific in nascent calli |
| chr7:10181599 | 358 | LTR/Copia | chr8:1108117-1108477 | 97.32 | Specific in nascent calli |
| chr8:16398062 | 364 | LTR/Copia | chr8:4927273-4927635 | 99.17 | Specific in nascent calli |
| chr4:4744855 | 605 | LTR/Copia | chr8:6056307-6058047 | 96.22 | Specific in nascent calli |
| chr7:31861494 | 833 | LTR/Copia | chr8:6637743-6638590 | 96.46 | Specific in nascent calli |
| chr6:7906534 | 456 | LTR/Copia | chr8:7166020-7166467 | 93.32 | Shared by nascent and old calli |
| chr6:12711459 | 449 | LTR/Copia | chr8:7166020-7166467 | 93.42 | Specific in nascent calli |
| chr2:9354528 | 359 | LTR/Copia | chr8:12403144-12403505 | 98.31 | Specific in nascent calli |
| chr4:16401954 | 71 | LTR/Copia | chr8:14094378-14095678 | 98.59 | Specific in nascent calli |
| chr4:16365984 | 207 | LTR/Copia | chr8:17484707-17484902 | 95.90 | Specific in nascent calli |
| chr2:8876038 | 874 | LTR/Copia | chr8:19770096-19770992 | 96.79 | Shared by nascent and old calli |
| chr4:15383140 | 418 | LTR/Copia | chr8:20003498-20003916 | 95.50 | Specific in nascent calli |
| chr9:16014528 | 99 | LTR/Copia | chr9:7353070-7357971 | 94.95 | Specific in nascent calli |
| chr4:972820 | 363 | LTR/Copia | chr9:7987743-7988104 | 99.45 | Specific in nascent calli |
| chr4:13352232 | 363 | LTR/Copia | chr9:7987743-7988104 | 99.45 | Specific in nascent calli |
| chr7:27541255 | 363 | LTR/Copia | chr9:7987743-7988104 | 99.45 | Specific in nascent calli |
| chr7:30277031 | 363 | LTR/Copia | chr9:7987743-7988104 | 99.72 | Specific in nascent calli |
| chr9:20816207 | 362 | LTR/Copia | chr9:7987743-7988104 | 99.72 | Specific in nascent calli |
| chr7:29396472 | 647 | LTR/Copia | chr9:9135351-9140564 | 96.76 | Specific in nascent calli |
| chr9:15189098 | 75 | LTR/Copia | chr9:9517902-9519940 | 100.00 | Specific in nascent calli |
| chr9:5991981 | 685 | LTR/Copia | chr9:11928341-11929033 | 95.38 | Specific in nascent calli |
| chr9:12864785 | 173 | LTR/Copia | chr9:12811690-12811980 | 87.43 | Shared by nascent and old calli |
| chr2:17885121 | 84 | LTR/Copia | chr9:13840381-13842132 | 100.00 | Specific in nascent calli |
| chr5:8756249 | 201 | LTR/Copia | chr9:16383330-16383534 | 88.33 | Specific in nascent calli |
| chr9:14312400 | 209 | LTR/Copia | chr9:16383330-16383534 | 89.80 | Specific in nascent calli |
| chr3:3035812 | 195 | LTR/Copia | chr9:16759314-16759500 | 98.40 | Specific in nascent calli |
| chr1:25235401 | 209 | LTR/Copia | chr9:19347923-19348124 | 90.89 | Shared by nascent and old calli |
| chr2:1855747 | 211 | LTR/Copia | chr9:19347923-19348124 | 90.89 | Specific in nascent calli |
| chr4:23052527 | 211 | LTR/Copia | chr9:19347923-19348124 | 90.89 | Specific in nascent calli |
| chr4:23633349 | 196 | LTR/Copia | chr9:19347923-19348124 | 90.42 | Specific in nascent calli |
| chr4:24163653 | 209 | LTR/Copia | chr9:19347923-19348124 | 90.66 | Specific in nascent calli |
| chr4:24358836 | 209 | LTR/Copia | chr9:19347923-19348124 | 90.39 | Specific in nascent calli |
| chr6:16199972 | 201 | LTR/Copia | chr9:19347923-19348124 | 90.80 | Specific in nascent calli |
| chr9:6512189 | 206 | LTR/Copia | chr9:19347923-19348124 | 90.80 | Specific in nascent calli |
| chr9:12524887 | 211 | LTR/Copia | chr9:19347923-19348124 | 90.89 | Specific in nascent calli |
| chr3:33168801 | 205 | LTR/Copia | chr9:19592808-19593009 | 94.74 | Specific in nascent calli |
| chr3:17077537 | 211 | LTR/Copia | chr9:20559197-20559398 | 89.90 | Specific in nascent calli |
| chr4:8489452 | 208 | LTR/Copia | chr9:20559197-20559398 | 86.94 | Specific in nascent calli |
| chr5:30191290 | 193 | LTR/Copia | chr9:23735550-23735751 | 99.43 | Specific in nascent calli |
| chr7:12641543 | 211 | LTR/Copia | chr9:24508786-24508987 | 86.94 | Specific in nascent calli |
| chr8:21724438 | 212 | LTR/Copia | chr9:24508786-24508987 | 87.06 | Specific in nascent calli |
| chr9:9817509 | 80 | LTR/Copia | chrUn:4756188-4757524 | 95.00 | Specific in nascent calli |
| chr6:20430763 | 330 | LTR/Copia | chrUn:5775158-5775476 | 93.71 | Specific in nascent calli |
| chr5:26759303 | 707 | LTR/Copia | chrUn:6475682-6476396 | 93.81 | Specific in nascent calli |
| chr3:18195995 | 121 | LTR/Copia | chrUn:7232759-7233649 | 99.17 | Shared by nascent and old calli |
| chr4:25975777 | 211 | LTR/Copia | chrUn:10975834-10976035 | 99.01 | Specific in nascent calli |
| chr2:26636806 | 86 | LTR/Copia | chrUn:12802906-12807243 | 100.00 | Specific in nascent calli |
| chr9:9764344 | 211 | LTR/Copia | chrUn:14052933-14053134 | 96.53 | Specific in nascent calli |
| chr9:4305944 | 418 | LTR/Copia | chrUn:30172772-30173188 | 94.74 | Specific in nascent calli |
| chr4:23737435 | 880 | LTR/Copia | chrUn:40101485-40102367 | 96.49 | Specific in nascent calli |
| chr5:15976962 | 57 | LTR/Copia | chrUn:45837779-45839032 | 100.00 | Specific in nascent calli |
| chr7:28352314 | 200 | LTR/Copia | chrUn:55370953-55371153 | 92.31 | Specific in nascent calli |
| chr9:20156648 | 81 | LTR/Copia | chrUn:55528310-55529147 | 97.59 | Specific in nascent calli |
| chr5:19191278 | 72 | LTR/Copia | chrUn:55903171-55903867 | 100.00 | Specific in nascent calli |
| chr9:4672991 | 71 | LTR/Copia | chrUn:55903171-55903867 | 100.00 | Specific in nascent calli |
| chr8:12595063 | 222 | LTR/Copia | chrUn:57918358-57925015 | 100.00 | Specific in nascent calli |
| chr9:21644611 | 98 | LTR/Copia | chrUn:57925256-57927117 | 100.00 | Shared by nascent and old calli |
| chr1:14623599 | 81 | LTR/Copia | chrUn:62641908-62642105 | 100.00 | Specific in nascent calli |
| chr5:4927620 | 364 | LTR/Copia | chrUn:66495119-66495479 | 99.44 | Specific in nascent calli |
| chr8:4169324 | 362 | LTR/Copia | chrUn:66495119-66495479 | 99.72 | Specific in nascent calli |
| chr8:13915161 | 363 | LTR/Copia | chrUn:66495119-66495479 | 99.72 | Specific in nascent calli |
| chr3:16435352 | 81 | LTR/Copia | chrUn:66527480-66527675 | 100.00 | Specific in nascent calli |
| chr4:22603354 | 144 | LTR/Gypsy | chr1:3451745-3452402 | 93.23 | Specific in nascent calli |
| chr2:28182288 | 683 | LTR/Gypsy | chr1:4108929-4109603 | 99.55 | Specific in nascent calli |
| chr1:24855945 | 620 | LTR/Gypsy | chr1:11475268-11475894 | 99.19 | Specific in nascent calli |
| chr3:5337273 | 625 | LTR/Gypsy | chr1:11475268-11475894 | 98.56 | Shared by nascent and old calli |
| chr5:1564215 | 622 | LTR/Gypsy | chr1:11475268-11475894 | 99.84 | Specific in nascent calli |
| chr5:12603105 | 618 | LTR/Gypsy | chr1:11475268-11475894 | 98.87 | Specific in nascent calli |
| chr6:20401447 | 623 | LTR/Gypsy | chr1:11475268-11475894 | 99.84 | Specific in nascent calli |
| chr4:26687363 | 230 | LTR/Gypsy | chr1:13568036-13568259 | 83.70 | Specific in nascent calli |
| chr5:11577219 | 622 | LTR/Gypsy | chr1:20360574-20361197 | 99.35 | Specific in nascent calli |
| chr7:32187866 | 621 | LTR/Gypsy | chr1:20360574-20361197 | 99.03 | Specific in nascent calli |
| chr5:9384711 | 231 | LTR/Gypsy | chr2:1315340-1315563 | 88.43 | Specific in nascent calli |
| chr7:27834289 | 236 | LTR/Gypsy | chr2:4990610-4990830 | 99.55 | Specific in nascent calli |
| chr9:4160874 | 227 | LTR/Gypsy | chr2:7995292-7995510 | 88.07 | Specific in nascent calli |
| chr2:22591456 | 499 | LTR/Gypsy | chr2:19464499-19465565 | 91.60 | Shared by nascent and old calli |
| chr6:7465971 | 619 | LTR/Gypsy | chr2:19754661-19755285 | 97.90 | Specific in nascent calli |
| chr3:37493604 | 676 | LTR/Gypsy | chr2:26516157-26516830 | 97.03 | Specific in nascent calli |
| chr8:23023182 | 680 | LTR/Gypsy | chr2:26516157-26516830 | 97.78 | Specific in nascent calli |
| chr2:26531905 | 239 | LTR/Gypsy | chr2:26531409-26532022 | 97.53 | Specific in nascent calli |
| chr2:21501552 | 660 | LTR/Gypsy | chr2:28377388-28378374 | 98.85 | Specific in nascent calli |
| chr2:3180939 | 234 | LTR/Gypsy | chr2:28749686-28749909 | 100.00 | Specific in nascent calli |
| chr3:24251161 | 238 | LTR/Gypsy | chr2:28749686-28749909 | 98.64 | Specific in nascent calli |
| chr7:29642694 | 996 | LTR/Gypsy | chr3:345421-346408 | 97.67 | Specific in nascent calli |
| chr8:14851427 | 990 | LTR/Gypsy | chr3:345421-346408 | 96.75 | Specific in nascent calli |
| chr2:29442508 | 612 | LTR/Gypsy | chr3:572348-572972 | 97.57 | Shared by nascent and old calli |
| chr4:11384756 | 620 | LTR/Gypsy | chr3:572348-572972 | 98.71 | Specific in nascent calli |
| chr9:18656702 | 620 | LTR/Gypsy | chr3:572348-572972 | 98.67 | Specific in nascent calli |
| chr2:9960042 | 237 | LTR/Gypsy | chr3:1635512-1635735 | 94.09 | Specific in nascent calli |
| chr4:12921829 | 229 | LTR/Gypsy | chr3:1635512-1635735 | 98.66 | Specific in nascent calli |
| chr5:30491702 | 244 | LTR/Gypsy | chr3:1635512-1635735 | 98.65 | Specific in nascent calli |
| chr6:7541269 | 229 | LTR/Gypsy | chr3:1635512-1635735 | 96.23 | Specific in nascent calli |
| chr9:15998673 | 239 | LTR/Gypsy | chr3:1635512-1635735 | 98.66 | Specific in nascent calli |
| chr2:3631663 | 992 | LTR/Gypsy | chr3:2481773-2482761 | 98.99 | Specific in nascent calli |
| chr4:9648429 | 998 | LTR/Gypsy | chr3:2481773-2482761 | 99.80 | Specific in nascent calli |
| chr5:29872643 | 994 | LTR/Gypsy | chr3:2481773-2482761 | 98.58 | Specific in nascent calli |
| chr7:9506975 | 997 | LTR/Gypsy | chr3:2481773-2482761 | 99.70 | Specific in nascent calli |
| chr9:4087911 | 1003 | LTR/Gypsy | chr3:2481773-2482761 | 98.98 | Specific in nascent calli |
| chr5:8794601 | 995 | LTR/Gypsy | chr3:5114099-5115084 | 98.78 | Shared by nascent and old calli |
| chr7:17434655 | 112 | LTR/Gypsy | chr3:9067531-9067778 | 95.41 | Specific in nascent calli |
| chr9:16051971 | 196 | LTR/Gypsy | chr3:10723842-10726769 | 97.34 | Specific in nascent calli |
| chr9:19714371 | 931 | LTR/Gypsy | chr3:11408214-11409521 | 94.76 | Specific in nascent calli |
| chr9:22168968 | 623 | LTR/Gypsy | chr3:13876508-13877132 | 99.36 | Specific in nascent calli |
| chr4:15950280 | 237 | LTR/Gypsy | chr3:17729934-17730157 | 88.94 | Shared by nascent and old calli |
| chr7:14319970 | 494 | LTR/Gypsy | chr3:18549176-18549918 | 92.70 | Shared by nascent and old calli |
| chr4:12874232 | 622 | LTR/Gypsy | chr3:19515965-19516593 | 99.68 | Specific in nascent calli |
| chr6:13424112 | 623 | LTR/Gypsy | chr3:19515965-19516593 | 99.03 | Specific in nascent calli |
| chr5:13049815 | 92 | LTR/Gypsy | chr3:21078386-21080397 | 90.70 | Specific in nascent calli |
| chr5:27629913 | 234 | LTR/Gypsy | chr3:24226909-24227130 | 98.19 | Specific in nascent calli |
| chr9:3678606 | 223 | LTR/Gypsy | chr3:24226909-24227130 | 99.09 | Specific in nascent calli |
| chr1:10624190 | 622 | LTR/Gypsy | chr3:25408230-25408862 | 99.36 | Specific in nascent calli |
| chr6:2040679 | 620 | LTR/Gypsy | chr3:25442134-25442764 | 97.43 | Specific in nascent calli |
| chr5:2020951 | 688 | LTR/Gypsy | chr3:26571019-26574178 | 98.98 | Specific in nascent calli |
| chr9:33078 | 688 | LTR/Gypsy | chr3:26571019-26574178 | 98.68 | Specific in nascent calli |
| chr7:29487746 | 281 | LTR/Gypsy | chr3:27541617-27543925 | 93.57 | Specific in nascent calli |
| chr1:10577862 | 998 | LTR/Gypsy | chr3:32153298-32154285 | 99.19 | Specific in nascent calli |
| chr2:17034868 | 998 | LTR/Gypsy | chr3:32153298-32154285 | 98.08 | Specific in nascent calli |
| chr4:11940734 | 998 | LTR/Gypsy | chr3:32153298-32154285 | 99.39 | Specific in nascent calli |
| chr6:8869636 | 995 | LTR/Gypsy | chr3:32153298-32154285 | 98.58 | Specific in nascent calli |
| chr9:8451576 | 592 | LTR/Gypsy | chr3:35348533-35349152 | 99.15 | Specific in nascent calli |
| chr9:20884302 | 622 | LTR/Gypsy | chr3:35348533-35349152 | 97.41 | Shared by nascent and old calli |
| chr4:8218243 | 729 | LTR/Gypsy | chr4:4409587-4410323 | 77.35 | Specific in nascent calli |
| chr2:4879334 | 225 | LTR/Gypsy | chr4:7022365-7022588 | 96.36 | Specific in nascent calli |
| chr2:10856809 | 208 | LTR/Gypsy | chr4:7022365-7022588 | 100.00 | Specific in nascent calli |
| chr3:29601701 | 234 | LTR/Gypsy | chr4:7022365-7022588 | 98.14 | Specific in nascent calli |
| chr3:31231322 | 146 | LTR/Gypsy | chr4:7022365-7022588 | 100.00 | Specific in nascent calli |
| chr4:9499529 | 219 | LTR/Gypsy | chr4:7022365-7022588 | 99.55 | Specific in nascent calli |
| chr4:18896190 | 236 | LTR/Gypsy | chr4:7022365-7022588 | 99.54 | Specific in nascent calli |
| chr5:2931494 | 222 | LTR/Gypsy | chr4:7022365-7022588 | 98.62 | Specific in nascent calli |
| chr5:7326821 | 232 | LTR/Gypsy | chr4:7022365-7022588 | 97.72 | Specific in nascent calli |
| chr5:8258815 | 238 | LTR/Gypsy | chr4:7022365-7022588 | 100.00 | Specific in nascent calli |
| chr5:9917086 | 231 | LTR/Gypsy | chr4:7022365-7022588 | 97.26 | Specific in nascent calli |
| chr6:17219950 | 234 | LTR/Gypsy | chr4:7022365-7022588 | 99.10 | Specific in nascent calli |
| chr6:19913336 | 236 | LTR/Gypsy | chr4:7022365-7022588 | 97.26 | Shared by nascent and old calli |
| chr7:6044522 | 231 | LTR/Gypsy | chr4:7022365-7022588 | 98.20 | Specific in nascent calli |
| chr7:8944829 | 231 | LTR/Gypsy | chr4:7022365-7022588 | 95.09 | Specific in nascent calli |
| chr7:11276689 | 234 | LTR/Gypsy | chr4:7022365-7022588 | 100.00 | Specific in nascent calli |
| chr7:25482645 | 235 | LTR/Gypsy | chr4:7022365-7022588 | 98.20 | Specific in nascent calli |
| chr8:3585305 | 237 | LTR/Gypsy | chr4:7022365-7022588 | 100.00 | Specific in nascent calli |
| chr8:16508970 | 243 | LTR/Gypsy | chr4:7022365-7022588 | 100.00 | Specific in nascent calli |
| chr8:20742693 | 168 | LTR/Gypsy | chr4:7022365-7022588 | 100.00 | Specific in nascent calli |
| chr9:16522713 | 227 | LTR/Gypsy | chr4:7022365-7022588 | 100.00 | Specific in nascent calli |
| chr9:20244108 | 232 | LTR/Gypsy | chr4:7022365-7022588 | 100.00 | Shared by nascent and old calli |
| chr9:22109754 | 233 | LTR/Gypsy | chr4:7022365-7022588 | 98.17 | Specific in nascent calli |
| chr1:23537730 | 227 | LTR/Gypsy | chr4:7519944-7520163 | 98.62 | Specific in nascent calli |
| chr2:8787628 | 220 | LTR/Gypsy | chr4:7519944-7520163 | 99.53 | Shared by nascent and old calli |
| chr5:12661700 | 232 | LTR/Gypsy | chr4:7519944-7520163 | 98.17 | Specific in nascent calli |
| chr4:8040142 | 124 | LTR/Gypsy | chr4:8039749-8040325 | 92.51 | Specific in nascent calli |
| chr4:14277410 | 121 | LTR/Gypsy | chr4:14489633-14490934 | 96.40 | Specific in nascent calli |
| chr9:9008819 | 360 | LTR/Gypsy | chr4:15081460-15090245 | 93.46 | Specific in nascent calli |
| chr2:7799919 | 693 | LTR/Gypsy | chr4:17100085-17100777 | 99.13 | Specific in nascent calli |
| chr9:1414605 | 681 | LTR/Gypsy | chr4:18670391-18671065 | 98.07 | Specific in nascent calli |
| chr5:3343525 | 763 | LTR/Gypsy | chr4:20864036-20864797 | 98.68 | Specific in nascent calli |
| chr9:2750561 | 840 | LTR/Gypsy | chr4:20864036-20864797 | 97.75 | Specific in nascent calli |
| chr3:31481502 | 611 | LTR/Gypsy | chr5:4575620-4576241 | 96.10 | Specific in nascent calli |
| chr1:28714355 | 129 | LTR/Gypsy | chr5:7613675-7613897 | 99.22 | Specific in nascent calli |
| chr4:5688055 | 239 | LTR/Gypsy | chr5:7613675-7613897 | 100.00 | Specific in nascent calli |
| chr3:33150546 | 228 | LTR/Gypsy | chr5:8064263-8064483 | 100.00 | Specific in nascent calli |
| chr3:33669928 | 235 | LTR/Gypsy | chr5:8064263-8064483 | 99.55 | Specific in nascent calli |
| chr4:9043721 | 226 | LTR/Gypsy | chr5:8064263-8064483 | 100.00 | Specific in nascent calli |
| chr4:24206392 | 207 | LTR/Gypsy | chr5:8064263-8064483 | 100.00 | Specific in nascent calli |
| chr5:20157631 | 140 | LTR/Gypsy | chr5:8064263-8064483 | 99.22 | Specific in nascent calli |
| chr7:32373937 | 218 | LTR/Gypsy | chr5:8064263-8064483 | 99.08 | Specific in nascent calli |
| chr8:11905298 | 236 | LTR/Gypsy | chr5:8064263-8064483 | 100.00 | Specific in nascent calli |
| chr4:19973012 | 242 | LTR/Gypsy | chr5:10189487-10189707 | 87.39 | Specific in nascent calli |
| chr4:25945288 | 619 | LTR/Gypsy | chr5:11043559-11044183 | 97.90 | Shared by nascent and old calli |
| chr4:1541892 | 229 | LTR/Gypsy | chr5:11225145-11225368 | 99.09 | Specific in nascent calli |
| chr5:36124637 | 616 | LTR/Gypsy | chr5:11845211-11845840 | 97.72 | Shared by nascent and old calli |
| chr7:12553388 | 614 | LTR/Gypsy | chr5:11845211-11845840 | 99.35 | Specific in nascent calli |
| chr8:12507551 | 622 | LTR/Gypsy | chr5:11845211-11845840 | 99.35 | Specific in nascent calli |
| chr4:24304034 | 977 | LTR/Gypsy | chr5:12191333-12192319 | 96.52 | Specific in nascent calli |
| chr4:25900800 | 998 | LTR/Gypsy | chr5:12191333-12192319 | 97.67 | Specific in nascent calli |
| chr4:6361494 | 225 | LTR/Gypsy | chr5:12376211-12376435 | 84.79 | Specific in nascent calli |
| chr7:28845581 | 204 | LTR/Gypsy | chr5:13687385-13687608 | 91.41 | Specific in nascent calli |
| chr3:26350808 | 151 | LTR/Gypsy | chr5:14211225-14215461 | 87.50 | Specific in nascent calli |
| chr6:4839224 | 301 | LTR/Gypsy | chr5:16087545-16088166 | 98.01 | Shared by nascent and old calli |
| chr4:21404495 | 223 | LTR/Gypsy | chr5:17279390-17279611 | 90.13 | Specific in nascent calli |
| chr5:18436622 | 82 | LTR/Gypsy | chr5:18435667-18443532 | 100.00 | Specific in nascent calli |
| chr3:36264270 | 236 | LTR/Gypsy | chr5:21354727-21354950 | 97.77 | Specific in nascent calli |
| chr9:558385 | 208 | LTR/Gypsy | chr5:21354727-21354950 | 97.60 | Specific in nascent calli |
| chr5:10990823 | 975 | LTR/Gypsy | chr5:22495517-22496478 | 97.40 | Specific in nascent calli |
| chr7:1420123 | 184 | LTR/Gypsy | chr5:24146968-24147187 | 96.20 | Specific in nascent calli |
| chr4:10523148 | 470 | LTR/Gypsy | chr5:25486359-25486829 | 96.60 | Specific in nascent calli |
| chr2:3812501 | 230 | LTR/Gypsy | chr5:25573943-25574166 | 99.55 | Specific in nascent calli |
| chr2:6348407 | 227 | LTR/Gypsy | chr5:25573943-25574166 | 100.00 | Specific in nascent calli |
| chr3:8227733 | 224 | LTR/Gypsy | chr5:25573943-25574166 | 100.00 | Specific in nascent calli |
| chr7:29414453 | 238 | LTR/Gypsy | chr5:25573943-25574166 | 98.21 | Specific in nascent calli |
| chr8:15727972 | 237 | LTR/Gypsy | chr5:25573943-25574166 | 94.64 | Specific in nascent calli |
| chr9:4026381 | 238 | LTR/Gypsy | chr5:25573943-25574166 | 97.21 | Specific in nascent calli |
| chr9:19777616 | 238 | LTR/Gypsy | chr5:25573943-25574166 | 99.55 | Specific in nascent calli |
| chr2:16479814 | 625 | LTR/Gypsy | chr5:27316134-27316758 | 99.00 | Specific in nascent calli |
| chr3:23948813 | 623 | LTR/Gypsy | chr5:27316134-27316758 | 97.43 | Specific in nascent calli |
| chr7:18396566 | 622 | LTR/Gypsy | chr5:27316134-27316758 | 99.20 | Specific in nascent calli |
| chr6:3871477 | 688 | LTR/Gypsy | chr5:28485655-28486340 | 97.96 | Specific in nascent calli |
| chr3:36982924 | 236 | LTR/Gypsy | chr5:29151754-29151976 | 95.95 | Specific in nascent calli |
| chr5:19241814 | 238 | LTR/Gypsy | chr5:29479261-29479477 | 99.54 | Specific in nascent calli |
| chr1:13057629 | 57 | LTR/Gypsy | chr5:30408587-30408855 | 91.23 | Specific in nascent calli |
| chr3:16400707 | 86 | LTR/Gypsy | chr5:30663269-30663670 | 100.00 | Specific in nascent calli |
| chr5:5565433 | 225 | LTR/Gypsy | chr5:37121890-37122113 | 93.95 | Specific in nascent calli |
| chr5:37845254 | 68 | LTR/Gypsy | chr5:37839165-37846012 | 98.15 | Specific in nascent calli |
| chr4:8093907 | 234 | LTR/Gypsy | chr5:38012466-38012688 | 85.92 | Specific in nascent calli |
| chr1:24824003 | 236 | LTR/Gypsy | chr5:38849199-38849423 | 97.75 | Shared by nascent and old calli |
| chr6:4875263 | 114 | LTR/Gypsy | chr6:4875152-4875434 | 87.61 | Specific in nascent calli |
| chr3:29992042 | 239 | LTR/Gypsy | chr6:5594291-5594513 | 98.18 | Shared by nascent and old calli |
| chr9:22606473 | 240 | LTR/Gypsy | chr6:5594291-5594513 | 97.30 | Specific in nascent calli |
| chr3:37534794 | 523 | LTR/Gypsy | chr6:9209692-9210225 | 95.62 | Specific in nascent calli |
| chr9:11220343 | 528 | LTR/Gypsy | chr6:9209692-9210225 | 95.46 | Shared by nascent and old calli |
| chr2:25258008 | 221 | LTR/Gypsy | chr6:16501742-16501965 | 99.08 | Shared by nascent and old calli |
| chr9:4591485 | 231 | LTR/Gypsy | chr6:16501742-16501965 | 95.39 | Specific in nascent calli |
| chr6:16542803 | 234 | LTR/Gypsy | chr7:887358-887581 | 94.55 | Specific in nascent calli |
| chr3:19430933 | 450 | LTR/Gypsy | chr7:7962773-7968151 | 99.44 | Specific in nascent calli |
| chr1:27216078 | 238 | LTR/Gypsy | chr7:12876855-12877078 | 96.86 | Shared by nascent and old calli |
| chr4:6091936 | 231 | LTR/Gypsy | chr7:12876855-12877078 | 97.73 | Specific in nascent calli |
| chr5:37151857 | 621 | LTR/Gypsy | chr7:19879771-19880396 | 97.88 | Specific in nascent calli |
| chr7:21506342 | 78 | LTR/Gypsy | chr7:21506256-21506778 | 97.59 | Shared by nascent and old calli |
| chr9:9091897 | 581 | LTR/Gypsy | chr7:22354490-22357969 | 96.39 | Specific in nascent calli |
| chr2:3884857 | 213 | LTR/Gypsy | chr8:586780-586997 | 84.16 | Specific in nascent calli |
| chr1:22609467 | 995 | LTR/Gypsy | chr8:3933315-3934300 | 98.37 | Specific in nascent calli |
| chr1:26870928 | 993 | LTR/Gypsy | chr8:3933315-3934300 | 98.88 | Specific in nascent calli |
| chr4:8776939 | 996 | LTR/Gypsy | chr8:3933315-3934300 | 98.58 | Specific in nascent calli |
| chr8:22602770 | 989 | LTR/Gypsy | chr8:3933315-3934300 | 99.07 | Specific in nascent calli |
| chr1:13155895 | 825 | LTR/Gypsy | chr8:5517496-5518100 | 93.36 | Shared by nascent and old calli |
| chr2:3888195 | 825 | LTR/Gypsy | chr8:5517496-5518100 | 93.72 | Specific in nascent calli |
| chr2:6568990 | 825 | LTR/Gypsy | chr8:5517496-5518100 | 93.72 | Shared by nascent and old calli |
| chr2:25968626 | 232 | LTR/Gypsy | chr8:7665878-7666099 | 100.00 | Specific in nascent calli |
| chr3:23900788 | 90 | LTR/Gypsy | chr8:7665878-7666099 | 100.00 | Specific in nascent calli |
| chr3:32859337 | 236 | LTR/Gypsy | chr8:7665878-7666099 | 99.55 | Specific in nascent calli |
| chr4:11804491 | 622 | LTR/Gypsy | chr8:7709624-7710250 | 97.58 | Specific in nascent calli |
| chr5:5935100 | 615 | LTR/Gypsy | chr8:7709624-7710250 | 96.14 | Specific in nascent calli |
| chr9:21867383 | 229 | LTR/Gypsy | chr8:7996816-7997039 | 90.95 | Specific in nascent calli |
| chr5:19137087 | 100 | LTR/Gypsy | chr8:8684027-8692355 | 99.00 | Specific in nascent calli |
| chr9:4272419 | 728 | LTR/Gypsy | chr8:9164058-9170547 | 95.72 | Specific in nascent calli |
| chr5:19335925 | 97 | LTR/Gypsy | chr8:9386432-9387931 | 100.00 | Specific in nascent calli |
| chr4:11859068 | 249 | LTR/Gypsy | chr8:10693019-10698587 | 96.37 | Shared by nascent and old calli |
| chr7:2186709 | 235 | LTR/Gypsy | chr8:12271168-12271387 | 91.36 | Specific in nascent calli |
| chr2:11288305 | 999 | LTR/Gypsy | chr8:13243666-13244654 | 98.89 | Shared by nascent and old calli |
| chr8:10728790 | 950 | LTR/Gypsy | chr8:17760485-17765905 | 96.64 | Specific in nascent calli |
| chr4:8672725 | 231 | LTR/Gypsy | chr8:20566533-20566753 | 90.95 | Specific in nascent calli |
| chr5:1243068 | 543 | LTR/Gypsy | chr8:21365353-21365894 | 92.78 | Specific in nascent calli |
| chr5:19369221 | 136 | LTR/Gypsy | chr9:2919498-2919721 | 99.22 | Shared by nascent and old calli |
| chr4:5684279 | 66 | LTR/Gypsy | chr9:6277166-6282743 | 95.24 | Shared by nascent and old calli |
| chr6:12091556 | 229 | LTR/Gypsy | chr9:9262240-9262461 | 96.85 | Specific in nascent calli |
| chr2:3171525 | 97 | LTR/Gypsy | chr9:13222254-13222478 | 97.85 | Specific in nascent calli |
| chr7:13897516 | 264 | LTR/Gypsy | chr9:14875954-14876577 | 99.24 | Specific in nascent calli |
| chr6:15573670 | 231 | LTR/Gypsy | chr9:16763348-16763566 | 95.43 | Specific in nascent calli |
| chr9:2179041 | 229 | LTR/Gypsy | chr9:16763348-16763566 | 93.49 | Specific in nascent calli |
| chr2:5499262 | 210 | LTR/Gypsy | chr9:18007128-18007349 | 99.05 | Specific in nascent calli |
| chr9:1555659 | 149 | LTR/Gypsy | chr9:18007128-18007349 | 95.62 | Specific in nascent calli |
| chr9:1560255 | 223 | LTR/Gypsy | chr9:18007128-18007349 | 97.12 | Shared by nascent and old calli |
| chr3:27373639 | 545 | LTR/Gypsy | chr9:19578980-19579519 | 98.15 | Specific in nascent calli |
| chr3:28405269 | 93 | LTR/Gypsy | chr9:23408310-23408533 | 100.00 | Specific in nascent calli |
| chr4:7898956 | 76 | LTR/Gypsy | chrUn:1636780-1637780 | 98.68 | Specific in nascent calli |
| chr6:14426309 | 267 | LTR/Gypsy | chrUn:3730401-3731971 | 98.83 | Shared by nascent and old calli |
| chr2:21771334 | 117 | LTR/Gypsy | chrUn:4181728-4181950 | 97.44 | Specific in nascent calli |
| chr7:14789398 | 137 | LTR/Gypsy | chrUn:4181728-4181950 | 98.54 | Specific in nascent calli |
| chr3:27029653 | 241 | LTR/Gypsy | chrUn:4625964-4626186 | 82.73 | Specific in nascent calli |
| chr6:20476055 | 235 | LTR/Gypsy | chrUn:13475896-13476119 | 98.21 | Specific in nascent calli |
| chr1:484175 | 990 | LTR/Gypsy | chrUn:13606376-13607361 | 98.88 | Specific in nascent calli |
| chr1:15150828 | 997 | LTR/Gypsy | chrUn:13606376-13607361 | 98.78 | Specific in nascent calli |
| chr3:25680282 | 993 | LTR/Gypsy | chrUn:13606376-13607361 | 98.28 | Specific in nascent calli |
| chr4:21154403 | 992 | LTR/Gypsy | chrUn:13606376-13607361 | 98.07 | Specific in nascent calli |
| chr5:8959801 | 994 | LTR/Gypsy | chrUn:13606376-13607361 | 98.38 | Specific in nascent calli |
| chr6:13665071 | 965 | LTR/Gypsy | chrUn:13606376-13607361 | 98.54 | Specific in nascent calli |
| chr9:6311953 | 996 | LTR/Gypsy | chrUn:13606376-13607361 | 98.99 | Specific in nascent calli |
| chr5:18129837 | 56 | LTR/Gypsy | chrUn:31294884-31295662 | 98.07 | Specific in nascent calli |
| chr4:23885024 | 106 | LTR/Gypsy | chrUn:40035080-40035298 | 95.00 | Specific in nascent calli |
| chr3:15594584 | 67 | LTR/Gypsy | chrUn:45395071-45395988 | 92.31 | Shared by nascent and old calli |
| chr1:14619440 | 222 | LTR/Gypsy | chrUn:55649165-55649386 | 99.55 | Specific in nascent calli |
| chr3:37471520 | 235 | LTR/Gypsy | chrUn:55649165-55649386 | 99.10 | Specific in nascent calli |
| chr6:13332388 | 230 | LTR/Gypsy | chrUn:55649165-55649386 | 99.55 | Specific in nascent calli |
| chr7:601513 | 57 | LTR/Gypsy | chrUn:55649165-55649386 | 98.25 | Specific in nascent calli |
| chr7:7583949 | 232 | LTR/Gypsy | chrUn:55649165-55649386 | 99.55 | Specific in nascent calli |
| chr7:30037407 | 234 | LTR/Gypsy | chrUn:55649165-55649386 | 99.10 | Specific in nascent calli |
| chr3:9056658 | 68 | LTR/Gypsy | chrUn:56253879-56255006 | 100.00 | Specific in nascent calli |
| chr8:10876361 | 54 | LTR/Gypsy | chrUn:57601500-57610755 | 100.00 | Specific in nascent calli |
| chr3:9140868 | 52 | LTR/Gypsy | chrUn:58721776-58723588 | 100.00 | Specific in nascent calli |
| chr6:19178536 | 798 | LTR/Gypsy | chrUn:59135082-59139325 | 96.08 | Specific in nascent calli |
| chr1:15806033 | 82 | LTR/Gypsy | chrUn:59366707-59366927 | 86.96 | Specific in nascent calli |
| chr3:6166411 | 54 | LTR/Gypsy | chrUn:62701492-62702361 | 100.00 | Specific in nascent calli |
| chr9:3217163 | 113 | LTR/Gypsy | chrUn:63434163-63436303 | 100.00 | Specific in nascent calli |
| chr1:10929630 | 151 | LTR/Gypsy | chrUn:64104081-64113119 | 100.00 | Specific in nascent calli |
| chr8:12694789 | 52 | LTR/Gypsy | chrUn:65991005-65992952 | 100.00 | Specific in nascent calli |
| chr8:7114586 | 66 | LTR/Gypsy | chrUn:66317987-66323627 | 98.48 | Specific in nascent calli |
| chr2:19556677 | 77 | LTR/Gypsy | chrUn:67235665-67247996 | 100.00 | Specific in nascent calli |
| chr5:14986405 | 58 | LTR/Gypsy | chrUn:67382602-67392239 | 100.00 | Specific in nascent calli |

| **Table S6. The list of genes with TE insertions in nascent calli into the gene body or upstream 2-kb region.** | | | | | | |
| --- | --- | --- | --- | --- | --- | --- |
| **Number** | **Gene ID** | **TE integration site** | **Gene element with TE insertion** | **Genomic coordinate of donating TE** | **Type of inserted TE** | **Gene functional annotation** |
| 1 | Cs1g_pb000290 | chr1:4669354 | Promoter | chr7:32419517-32420348 | DNA/hAT-Ac | S-adenosyl-l-methionine decarboxylase leader peptide |
| 2 | Cs1g_pb000500 | chr1:4526676 | Intron | chr3:22109696-22110511 | LTR/Copia | Pentatricopeptide repeat-containing protein At4g19220, mitochondrial |
| 3 | Cs1g_pb000610 | chr1:4417584 | Promoter | chr2:3259240-3259574 | DNA/hat | Probable serine/threonine-protein kinase WNK6 |
| 4 | Cs1g_pb000720 | chr1:4260277 | Intron | chr2:31454573-31454912 | DNA/hat | Regulator of nonsense transcripts 1 homolog |
|  |  | chr1:4261480 | Intron | chr8:11070940-11074525 | DNA/hAT-Ac |  |
| 5 | Cs1g_pb003700 | chr1:684084 | Promoter | chrUn:58751226-58752100 | DNA/hAT-Ac | GAG-POL-RELATED RETROTRANSPOSON;PREDICTED PROTEIN (FRAGMENT) |
| 6 | Cs1g_pb004740 | chr1:5678166 | Promoter | chr5:23584857-23588361 | DNA/hAT-Ac | Uncharacterized protein |
| 7 | Cs1g_pb004750 | chr1:5678166 | 3'UTR | chr5:23584857-23588361 | DNA/hAT-Ac | tRNA uridine 5-carboxymethylaminomethyl modification enzyme MnmG |
| 8 | Cs1g_pb005280 | chr1:6530866 | Intron | chr1:17851264-17851680 | DNA/PIF-Harbinger | CBS domain-containing protein CBSCBSPB3;INOSINE-5-MONOPHOSPHATE DEHYDROGENASE RELATED |
| 9 | Cs1g_pb006980 | chr1:10577862 | Intron | chr3:32153298-32154285 | LTR/Gypsy | Beta-amyrin synthase |
|  |  | chr1:10624190 | Intron | chr3:25408230-25408862 | LTR/Gypsy |  |
| 10 | Cs1g_pb008390 | chr1:8216710 | 5'UTR | chr5:36791354-36792062 | LINE/L2 | 50S ribosomal protein L17 |
| 11 | Cs1g_pb008460 | chr1:8166946 | 3'UTR | chr8:11070940-11074525 | DNA/hAT-Ac | Uridylate kinase |
| 12 | Cs1g_pb008750 | chr1:13066899 | Promoter | chr2:8028759-8029489 | LTR/Copia | 60S ribosomal protein L7-4 |
| 13 | Cs1g_pb008760 | chr1:13113200 | 5'UTR | chrUn:65257601-65257912 | DNA/hat | Cytochrome P450 82C4 |
| 14 | Cs1g_pb010590 | chr1:15806033 | 5'UTR | chrUn:59366707-59366927 | LTR/Gypsy | Defective in cullin neddylation protein |
| 15 | Cs1g_pb015120 | chr1:21011707 | 5'UTR | chr9:8834429-8835144 | LINE/L2 | MMS19 nucleotide excision repair protein homolog |
| 16 | Cs1g_pb017020 | chr1:22609467 | 5'UTR | chr8:3933315-3934300 | LTR/Gypsy | Uncharacterized protein |
| 17 | Cs1g_pb018890 | chr1:24855945 | Promoter | chr1:11475268-11475894 | LTR/Gypsy | Putative pentatricopeptide repeat-containing protein At5g65820 |
| 18 | Cs1g_pb020540 | chr1:23537730 | Promoter | chr4:7519944-7520163 | LTR/Gypsy | Extradiol ring-cleavage dioxygenase |
| 19 | Cs1g_pb020680 | chr1:23449971 | Promoter | chrUn:57677243-57677582 | DNA/hat | Protein DYAD |
| 20 | Cs1g_pb020690 | chr1:23449971 | Promoter | chrUn:57677243-57677582 | DNA/hat | Auxin-induced protein X15 |
| 21 | Cs1g_pb022370 | chr1:26870928 | Promoter | chr8:3933315-3934300 | LTR/Gypsy | RING finger protein 5 |
| 22 | Cs1g_pb022470 | chr1:26940864 | Promoter | chr1:26940590-26940914 | LTR/Copia | Anthocyanidin 3-O-glucosyltransferase 5 |
| 23 | Cs1g_pb022770 | chr1:27216078 | 3'UTR | chr7:12876855-12877078 | LTR/Gypsy | Cytochrome P450 90B1 |
| 24 | Cs1g_pb023790 | chr1:27941149 | Promoter | chr5:23584857-23588361 | DNA/hAT-Ac | Kinesin-like protein NACK1 |
| 25 | Cs1g_pb024840 | chr1:28857723 | Promoter | chr8:2126956-2127275 | DNA/hat | Uncharacterized protein |
| 26 | Cs1g_pb025030 | chr1:28985171 | Promoter | chr5:23584857-23588361 | DNA/hAT-Ac | GDSL esterase/lipase At4g16230 |
| 27 | Cs1g_pb025040 | chr1:28985171 | Promoter | chr5:23584857-23588361 | DNA/hAT-Ac | Protein S-acyltransferase 10 |
| 28 | Cs2g_pb000560 | chr2:371715 | Promoter | chr5:14809407-14809738 | DNA/hat | CASP-like protein 2B2 |
| 29 | Cs2g_pb001340 | chr2:7044987 | Promoter | chr6:613256-613797 | DNA/MULE-MuDR | Aspartic proteinase nepenthesin-1 |
| 30 | Cs2g_pb001770 | chr2:6640571 | Promoter | chr5:2480771-2480987 | LTR/Copia | Vacuolar protein sorting-associated protein 54, chloroplastic |
| 31 | Cs2g_pb001830 | chr2:6594988 | Promoter | chrUn:5232478-5232730 | DNA/hat | Protein LIGHT-DEPENDENT SHORT HYPOCOTYLS 6 |
|  |  | chr2:6595408 | Promoter | chr8:427180-427597 | LTR/Copia |  |
| 32 | Cs2g_pb002180 | chr2:6293012 | Promoter | chr7:10250532-10251237 | DNA/PIF-Harbinger | Fatty acid 2-hydroxylase 1 |
| 33 | Cs2g_pb002240 | chr2:6264359 | Promoter | chrUn:55617636-55618629 | DNA/hAT-Ac | Tubulin beta-3 chain |
| 34 | Cs2g_pb002310 | chr2:6212883 | Promoter | chr2:26845269-26846126 | DNA/hAT-Ac | Probable LRR receptor-like serine/threonine-protein kinase At4g20940 |
| 35 | Cs2g_pb002330 | chr2:6212883 | Promoter | chr2:26845269-26846126 | DNA/hAT-Ac | GDP-L-galactose phosphorylase 1 |
| 36 | Cs2g_pb003150 | chr2:5558774 | 3'UTR | chr2:3259240-3259574 | DNA/hat | Piriformospora indica-insensitive protein 2 |
| 37 | Cs2g_pb003160 | chr2:5558774 | Promoter | chr2:3259240-3259574 | DNA/hat | Protein TRANSPARENT TESTA 12 |
| 38 | Cs2g_pb003250 | chr2:5499262 | Intron | chr9:18007128-18007349 | LTR/Gypsy | Cytochrome P450 716B1 |
| 39 | Cs2g_pb004180 | chr2:4879334 | Promoter | chr4:7022365-7022588 | LTR/Gypsy | Lysine-specific demethylase JMJ25 |
| 40 | Cs2g_pb005520 | chr2:3888195 | Promoter | chr8:5517496-5518100 | LTR/Gypsy | Pentatricopeptide repeat-containing protein At4g13650 |
| 41 | Cs2g_pb005530 | chr2:3884857 | Intron | chr8:586780-586997 | LTR/Gypsy | Protein DEHYDRATION-INDUCED 19 |
| 42 | Cs2g_pb005640 | chr2:3812501 | Promoter | chr5:25573943-25574166 | LTR/Gypsy | Serine/threonine-protein kinase-like protein ACR4 |
| 43 | Cs2g_pb005920 | chr2:3631663 | Promoter | chr3:2481773-2482761 | LTR/Gypsy | Pathogenesis-related genes transcriptional activator PTI5 |
| 44 | Cs2g_pb006570 | chr2:3166595 | Promoter | chr7:22183482-22184497 | LTR | Cellulose synthase A catalytic subunit 8 [UDP-forming] |
|  |  | chr2:3171525 | Intron | chr9:13222254-13222478 | LTR/Gypsy |  |
| 45 | Cs2g_pb006580 | chr2:3166595 | 3'UTR | chr7:22183482-22184497 | LTR | WD repeat-containing protein 53 |
| 46 | Cs2g_pb006790 | chr2:3011736 | 3'UTR | chr5:23584857-23588361 | DNA/hAT-Ac | BES1/BZR1 homolog protein 4 |
| 47 | Cs2g_pb009360 | chr2:11736195 | Promoter | chr5:19347692-19348108 | LTR/Copia | Uncharacterized protein |
| 48 | Cs2g_pb009610 | chr2:11288305 | 3'UTR | chr8:13243666-13244654 | LTR/Gypsy | Sodium/hydrogen exchanger 7 |
| 49 | Cs2g_pb010960 | chr2:9960042 | Promoter | chr3:1635512-1635735 | LTR/Gypsy | CLAVATA3/ESR (CLE)-related protein 16 |
| 50 | Cs2g_pb011670 | chr2:9354528 | 5'UTR | chr8:12403144-12403505 | LTR/Copia | Probable fructose-bisphosphate aldolase 3, chloroplastic |
| 51 | Cs2g_pb012440 | chr2:8787628 | Promoter | chr4:7519944-7520163 | LTR/Gypsy | Endonuclease 2 |
| 52 | Cs2g_pb012530 | chr2:8696985 | 3'UTR | chrUn:51634863-51635176 | DNA/hat | Uncharacterized protein |
| 53 | Cs2g_pb015460 | chr2:13649187 | Intron | chr2:13648695-13649485 | LTR/Copia | Uncharacterized protein isoform 1 |
| 54 | Cs2g_pb017020 | chr2:16900317 | Promoter | chr2:17287972-17288646 | LINE/L2 | Autophagy-related protein 8d |
| 55 | Cs2g_pb019150 | chr2:19775627 | Promoter | chrUn:60793494-60793826 | DNA/hat | LRR receptor-like serine/threonine-protein kinase GSO2 |
| 56 | Cs2g_pb019410 | chr2:19152632 | Promoter | chr4:19152800-19153612 | LTR/Copia | UDP-glycosyltransferase 74E2 |
| 57 | Cs2g_pb020190 | chr2:21501552 | Promoter | chr2:28377388-28378374 | LTR/Gypsy | Probable receptor-like protein kinase At5g47070 |
| 58 | Cs2g_pb020210 | chr2:21525727 | Promoter | chr5:38265607-38265799 | DNA | Phosphatidylinositol 4-phosphate 5-kinase 8 |
| 59 | Cs2g_pb020300 | chr2:21576191 | Promoter | chr3:23680705-23680909 | LTR/Copia | Trihelix transcription factor ASIL2 |
| 60 | Cs2g_pb020320 | chr2:21584603 | 3'UTR | chr1:11220318-11221096 | LTR/Copia | Pentatricopeptide repeat-containing protein At1g02150 |
| 61 | Cs2g_pb021130 | chr2:22521018 | Promoter | chr2:22519700-22521227 | LTR/Copia | DNA-directed RNA polymerase III subunit RPC1 |
| 62 | Cs2g_pb022070 | chr2:23902253 | Promoter | chr9:9223495-9223893 | DNA/hAT-Ac | Cytochrome P450 94B3 |
| 63 | Cs2g_pb023010 | chr2:25258008 | Promoter | chr6:16501742-16501965 | LTR/Gypsy | Secoisolariciresinol dehydrogenase (Fragment) |
| 64 | Cs2g_pb023080 | chr2:25329595 | 3'UTR | chr1:23493616-23494704 | DNA/hAT-Ac | Light-regulated protein |
| 65 | Cs2g_pb023720 | chr2:25968626 | Promoter | chr8:7665878-7666099 | LTR/Gypsy | G-type lectin S-receptor-like serine/threonine-protein kinase SD2-5 |
| 66 | Cs2g_pb024350 | chr2:26636806 | Promoter | chrUn:12802906-12807243 | LTR/Copia | Hexose carrier protein HEX6 |
| 67 | Cs2g_pb024380 | chr2:26658963 | Promoter | chr2:3113926-3114264 | DNA/hat | Hexose carrier protein HEX6 |
| 68 | Cs2g_pb024470 | chr2:26774250 | Promoter | chr3:30428668-30429423 | LINE/L2 | Uncharacterized protein |
| 69 | Cs2g_pb024600 | chr2:26906653 | Promoter | chr2:9305349-9306046 | LTR/Copia | Uncharacterized protein |
| 70 | Cs2g_pb024650 | chr2:27764224 | CDS | chr3:24940433-24940798 | LTR/Copia | LRR receptor-like serine/threonine-protein kinase GSO1 |
| 71 | Cs2g_pb027100 | chr2:29442508 | Promoter | chr3:572348-572972 | LTR/Gypsy | Protein prenyltransferase alpha subunit repeat-containing protein 1 |
| 72 | Cs2g_pb029340 | chr2:32443340 | Promoter | chr2:6844615-6844981 | LTR/Copia | Uncharacterized protein |
| 73 | Cs2g_pb029350 | chr2:32443340 | Intron | chr2:6844615-6844981 | LTR/Copia | Dihydropyrimidinase |
| 74 | Cs3g_pb001030 | chr3:1571816 | Promoter | chr7:13897504-13897912 | LTR/Copia | Mitotic spindle checkpoint protein MAD1 |
| 75 | Cs3g_pb004180 | chr3:8227733 | Promoter | chr5:25573943-25574166 | LTR/Gypsy | Somatic embryogenesis receptor kinase 2 |
| 76 | Cs3g_pb007710 | chr3:16053615 | Promoter | chr1:28630136-28630470 | DNA/hat | Cation/H(+) antiporter 1 |
| 77 | Cs3g_pb007860 | chr3:16256153 | Promoter | chr2:31454573-31454912 | DNA/hat | RNA polymerase II C-terminal domain phosphatase-like 4 |
| 78 | Cs3g_pb009480 | chr3:19430933 | CDS | chr7:7962773-7968151 | LTR/Gypsy | Disease resistance protein At4g27190 |
| 79 | Cs3g_pb010530 | chr3:20892680 | Promoter | chrUn:51471995-51472285 | DNA/hAT-Ac | Plastid lipid-associated protein 3, chloroplastic |
| 80 | Cs3g_pb010750 | chr3:21246665 | Promoter | chr5:23584857-23588361 | DNA/hAT-Ac | Uncharacterized protein |
| 81 | Cs3g_pb011820 | chr3:23948813 | Promoter | chr5:27316134-27316758 | LTR/Gypsy | CRC domain-containing protein TSO1 |
| 82 | Cs3g_pb011880 | chr3:23900788 | Promoter | chr8:7665878-7666099 | LTR/Gypsy | Translation initiation factor IF-2 |
| 83 | Cs3g_pb012810 | chr3:37534794 | Promoter | chr6:9209692-9210225 | LTR/Gypsy | Alkaline phosphatase D |
| 84 | Cs3g_pb013610 | chr3:36982924 | Promoter | chr5:29151754-29151976 | LTR/Gypsy | Small nuclear ribonucleoprotein SmD3b |
| 85 | Cs3g_pb013620 | chr3:36982924 | Promoter | chr5:29151754-29151976 | LTR/Gypsy | 1,4-alpha-glucan-branching enzyme 1, chloroplastic/amyloplastic (Fragment) |
| 86 | Cs3g_pb013770 | chr3:36913454 | Promoter | chr1:20939612-20940290 | DNA/MULE-MuDR | Uncharacterized protein |
| 87 | Cs3g_pb014750 | chr3:36268486 | Promoter | chr3:36508274-36508625 | DNA/hat | Snakin-2 |
| 88 | Cs3g_pb014870 | chr3:36187670 | Intron | chrUn:6353054-6353246 | DNA | Uncharacterized protein |
| 89 | Cs3g_pb016490 | chr3:35044856 | 3'UTR | chr5:24446391-24447083 | LTR/Copia | Exocyst complex component SEC3A |
| 90 | Cs3g_pb016500 | chr3:35044856 | 3'UTR | chr5:24446391-24447083 | LTR/Copia | Uncharacterized protein |
| 91 | Cs3g_pb016540 | chr3:35021806 | Promoter | chr3:17810150-17810568 | LTR/Copia | Allene oxide synthase, chloroplastic |
| 92 | Cs3g_pb016660 | chr3:34939277 | Promoter | chr6:9689944-9690854 | LTR/Copia | Lysine histidine transporter-like 8 |
| 93 | Cs3g_pb016730 | chr3:34893408 | Promoter | chr3:34893316-34893872 | DNA/hAT-Ac | Probable disease resistance protein At4g33300 |
| 94 | Cs3g_pb016970 | chr3:34724112 | Intron | chr3:34724336-34729145 | LTR/Copia | Patatin-like protein 2 |
| 95 | Cs3g_pb017050 | chr3:34689220 | Promoter | chr5:23584857-23588361 | DNA/hAT-Ac | Diphthamide biosynthesis protein 4 |
| 96 | Cs3g_pb018670 | chr3:33669928 | Promoter | chr5:8064263-8064483 | LTR/Gypsy | Zinc-finger homeodomain protein 2 |
| 97 | Cs3g_pb019300 | chr3:33202205 | Intron | chr3:24064163-24064855 | LTR/Copia | Abscisic stress-ripening protein 2 |
| 98 | Cs3g_pb019370 | chr3:33168801 | Promoter | chr9:19592808-19593009 | LTR/Copia | Sterol 3-beta-glucosyltransferase UGT80B1 |
| 99 | Cs3g_pb020640 | chr3:32291060 | Promoter | chr8:427180-427597 | LTR/Copia | NAD(P)H-quinone oxidoreductase subunit M, chloroplastic |
| 100 | Cs3g_pb021790 | chr3:31481502 | Promoter | chr5:4575620-4576241 | LTR/Gypsy | Phosphatidylinositol 4-phosphate 5-kinase 1 |
| 101 | Cs3g_pb022020 | chr3:31306186 | Promoter | chr5:38049175-38049376 | LTR/Copia | Uncharacterized protein |
| 102 | Cs3g_pb022030 | chr3:31306186 | Promoter | chr5:38049175-38049376 | LTR/Copia | Serine hydroxymethyltransferase 6 |
| 103 | Cs3g_pb022130 | chr3:31231322 | Promoter | chr4:7022365-7022588 | LTR/Gypsy | Probable glycerol-3-phosphate acyltransferase 3 |
| 104 | Cs3g_pb022510 | chr3:30930520 | Promoter | chr1:28736381-28737104 | LINE/L2 | Uncharacterized protein |
| 105 | Cs3g_pb022740 | chr3:30773623 | Promoter | chr4:19510264-19511240 | DNA/hAT-Ac | Uncharacterized protein |
| 106 | Cs3g_pb022750 | chr3:30773623 | 5'UTR | chr4:19510264-19511240 | DNA/hAT-Ac | Melanoma-associated antigen G1 |
| 107 | Cs3g_pb023040 | chr3:30466655 | Promoter | chr9:14061748-14062086 | DNA/hat | Uncharacterized protein |
| 108 | Cs3g_pb023580 | chr3:29992042 | Intron | chr6:5594291-5594513 | LTR/Gypsy | Putative protein NRT1/ PTR FAMILY 2.14 |
| 109 | Cs3g_pb024010 | chr3:29604172 | Promoter | chr3:33707429-33707749 | DNA/hat | Phosphoglycerate mutase-like protein 1 |
| 110 | Cs3g_pb025150 | chr3:28691303 | Promoter | chr4:12315438-12316399 | DNA/hAT-Ac | Acyl-[acyl-carrier-protein] desaturase 5, chloroplastic |
| 111 | Cs3g_pb025310 | chr3:28622367 | Promoter | chrUn:12669230-12669422 | DNA | Probable Xaa-Pro aminopeptidase 3 |
| 112 | Cs3g_pb025630 | chr3:28405269 | Intron | chr9:23408310-23408533 | LTR/Gypsy | Uncharacterized protein |
|  |  | chr3:28408117 | Promoter | chrUn:55617636-55618629 | DNA/hAT-Ac |  |
| 113 | Cs3g_pb027080 | chr3:27373639 | Promoter | chr9:19578980-19579519 | LTR/Gypsy | Zinc finger protein VAR3, chloroplastic |
| 114 | Cs3g_pb028910 | chr3:25272313 | Intron | chr8:4112990-4113571 | DNA/hAT-Ac | 3-oxo-Delta(4,5)-steroid 5-beta-reductase |
| 115 | Cs3g_pb029790 | chr3:24413122 | Promoter | chr9:19089941-19090002 | DNA/PIF-Harbinger | Cannabidiolic acid synthase-like 1 |
| 116 | Cs4g_pb000720 | chr4:940101 | Promoter | chr1:11156442-11157335 | LTR/Copia | Uncharacterized protein |
| 117 | Cs4g_pb000770 | chr4:972820 | Promoter | chr9:7987743-7988104 | LTR/Copia | Early nodulin-like protein 3 |
| 118 | Cs4g_pb000780 | chr4:972820 | Promoter | chr9:7987743-7988104 | LTR/Copia | Aspartic proteinase nepenthesin-1 |
| 119 | Cs4g_pb001160 | chr4:1213197 | Promoter | chrUn:51458372-51458713 | DNA/hat | Nuclear pore complex protein NUP43 |
| 120 | Cs4g_pb001170 | chr4:1213197 | Promoter | chrUn:51458372-51458713 | DNA/hat | Uncharacterized protein |
| 121 | Cs4g_pb001180 | chr4:1215879 | Promoter | chr5:28087626-28087939 | DNA/hat | Protein TSS |
| 122 | Cs4g_pb001380 | chr4:1396260 | Intron | chr3:25783558-25783759 | LTR/Copia | 5'-nucleotidase domain-containing protein DDB_G0275467 |
| 123 | Cs4g_pb001850 | chr4:1724043 | Intron | chr3:18915636-18915955 | DNA/hat | Glutamate receptor 2.8 |
| 124 | Cs4g_pb001860 | chr4:1724043 | Intron | chr3:18915636-18915955 | DNA/hat | Glutamate receptor 2.8 |
| 125 | Cs4g_pb001870 | chr4:1738390 | 5'UTR | chr5:29919457-29920181 | LINE/L2 | Small nuclear ribonucleoprotein E |
| 126 | Cs4g_pb003860 | chr4:3156864 | Promoter | chr3:31242167-31242533 | LTR/Copia | YTH domain-containing family protein 2 |
| 127 | Cs4g_pb003870 | chr4:3156864 | Promoter | chr3:31242167-31242533 | LTR/Copia | 30S ribosomal protein S17 |
| 128 | Cs4g_pb005160 | chr4:3999521 | Promoter | chr5:28087626-28087939 | DNA/hat | Protein TRANSPARENT TESTA 16 |
| 129 | Cs4g_pb005170 | chr4:3999521 | Promoter | chr5:28087626-28087939 | DNA/hat | Myosin-15 |
| 130 | Cs4g_pb005520 | chr4:4286004 | 3'UTR | chr2:2433641-2434525 | LTR/Copia | Alpha-aminoadipic semialdehyde synthase |
| 131 | Cs4g_pb006110 | chr4:4744855 | Promoter | chr8:6056307-6058047 | LTR/Copia | Uncharacterized protein |
| 132 | Cs4g_pb006250 | chr4:4833124 | Promoter | chr3:24940433-24940798 | LTR/Copia | RING-box protein 1a |
| 133 | Cs4g_pb007760 | chr4:5894137 | 3'UTR | chrUn:57677243-57677582 | DNA/hat | PROKAR_LIPOPROTEIN |
| 134 | Cs4g_pb007940 | chr4:6042850 | Promoter | chr6:4723748-4724466 | LINE/L2 | Cytochrome P450 71D11 (Fragment) |
| 135 | Cs4g_pb007980 | chr4:6091936 | CDS | chr7:12876855-12877078 | LTR/Gypsy | Cytochrome P450 71D11 (Fragment) |
| 136 | Cs4g_pb007990 | chr4:6091936 | Promoter | chr7:12876855-12877078 | LTR/Gypsy | Cytochrome P450 71D10 |
| 137 | Cs4g_pb008320 | chr4:6361494 | Promoter | chr5:12376211-12376435 | LTR/Gypsy | Pirin-like protein At1g50590 |
| 138 | Cs4g_pb008970 | chr4:6808556 | Promoter | chr2:5037384-5038103 | LINE/L2 | Plant invertase/pectin methylesterase inhibitor |
| 139 | Cs4g_pb009090 | chr4:6910725 | Promoter | chr5:36791354-36792062 | LINE/L2 | NAC domain-containing protein 7 |
| 140 | Cs4g_pb009820 | chr4:7932431 | Promoter | chr5:23584857-23588361 | DNA/hAT-Ac | Uncharacterized protein |
| 141 | Cs4g_pb009830 | chr4:7932431 | 5'UTR | chr5:23584857-23588361 | DNA/hAT-Ac | E3 ubiquitin-protein ligase RING1 |
| 142 | Cs4g_pb009900 | chr4:8093907 | Promoter | chr5:38012466-38012688 | LTR/Gypsy | Probable RNA-dependent RNA polymerase 3 |
| 143 | Cs4g_pb010020 | chr4:8218243 | Promoter | chr4:4409587-4410323 | LTR/Gypsy | DNA mismatch repair protein MSH6 |
| 144 | Cs4g_pb010070 | chr4:8245238 | Promoter | chr3:12690818-12691814 | DNA/hAT-Ac | Bromodomain and WD repeat-containing protein 1 |
| 145 | Cs4g_pb010380 | chr4:8459348 | Intron | chr1:25695620-25695957 | LTR/Copia | Nodal modulator 2 |
| 146 | Cs4g_pb010420 | chr4:8489452 | Promoter | chr9:20559197-20559398 | LTR/Copia | Probable pectinesterase 55 |
| 147 | Cs4g_pb010450 | chr4:8507641 | Promoter | chr8:15353806-15354070 | DNA/hat | Probable ubiquitin-conjugating enzyme E2 23 |
| 148 | Cs4g_pb010670 | chr4:8672725 | 3'UTR | chr8:20566533-20566753 | LTR/Gypsy | Plant invertase/pectin methylesterase inhibi |
| 149 | Cs4g_pb010680 | chr4:8672725 | Promoter | chr8:20566533-20566753 | LTR/Gypsy | Putative pentatricopeptide repeat-containing protein At1g02420 |
| 150 | Cs4g_pb011200 | chr4:9023094 | Promoter | chr5:29919457-29920181 | LINE/L2 | Charged multivesicular body protein 7 |
| 151 | Cs4g_pb011220 | chr4:9043721 | Promoter | chr5:8064263-8064483 | LTR/Gypsy | Transcription factor MYB86 |
| 152 | Cs4g_pb011990 | chr4:9648429 | 3'UTR | chr3:2481773-2482761 | LTR/Gypsy | Homeobox-leucine zipper protein ATHB-7 |
| 153 | Cs4g_pb012740 | chr4:10523148 | Intron | chr5:25486359-25486829 | LTR/Gypsy | HAUS augmin-like complex subunit 4 |
| 154 | Cs4g_pb013830 | chr4:11384756 | Promoter | chr3:572348-572972 | LTR/Gypsy | Ankyrin repeat-containing protein At3g12360 |
|  |  | chr4:11389567 | CDS | chr5:25031792-25036345 | LTR/Copia |  |
| 155 | Cs4g_pb014090 | chr4:11598644 | Promoter | chr5:11202740-11203039 | DNA/hat | Electron transfer flavoprotein subunit alpha, mitochondrial |
| 156 | Cs4g_pb014100 | chr4:11598644 | 5'UTR | chr5:11202740-11203039 | DNA/hat | Protein disulfide-isomerase 5-3 |
| 157 | Cs4g_pb015580 | chr4:13764103 | Promoter | chr5:3270307-3270496 | DNA | Probable LRR receptor-like serine/threonine-protein kinase At3g47570 |
| 158 | Cs4g_pb015970 | chr4:13352232 | Promoter | chr9:7987743-7988104 | LTR/Copia | Peptidase_M50B |
| 159 | Cs4g_pb016300 | chr4:12921829 | Promoter | chr3:1635512-1635735 | LTR/Gypsy | Glu S.griseus protease inhibitor |
| 160 | Cs4g_pb016310 | chr4:12916937 | CDS | chr4:12916244-12917233 | Simple_repeat | Endo-1,4-beta-xylanase B |
| 161 | Cs4g_pb016360 | chr4:12874232 | Promoter | chr3:19515965-19516593 | LTR/Gypsy | F-box protein At5g07670 |
| 162 | Cs4g_pb016420 | chr4:15950280 | Promoter | chr3:17729934-17730157 | LTR/Gypsy | Zinc finger CCCH domain-containing protein 64 |
| 163 | Cs4g_pb016660 | chr4:16365984 | Promoter | chr8:17484707-17484902 | LTR/Copia | Fanconi Anaemia group E protein, C-terminal |
| 164 | Cs4g_pb017060 | chr4:17049096 | 3'UTR | chr5:24446391-24447083 | LTR/Copia | Caffeoyl-CoA O-methyltransferase |
| 165 | Cs4g_pb017090 | chr4:17136163 | Intron | chr5:23584857-23588361 | DNA/hAT-Ac | Uncharacterized protein |
| 166 | Cs4g_pb018430 | chr4:19417052 | Intron | chr5:23584857-23588361 | DNA/hAT-Ac | Uncharacterized protein |
| 167 | Cs4g_pb018740 | chr4:19960864 | Promoter | chrUn:12471944-12472254 | DNA/hat | Auxin-responsive protein IAA27 |
| 168 | Cs4g_pb019830 | chr4:21573637 | Promoter | chrUn:58751226-58752100 | DNA/hAT-Ac | Cytochrome P450 734A1 |
| 169 | Cs4g_pb020150 | chr4:21262200 | Promoter | chr5:29570614-29571483 | LTR/Copia | Tudor domain-containing protein 3 |
| 170 | Cs4g_pb020220 | chr4:21183340 | Promoter | chr8:2126956-2127275 | DNA/hat | DnaJ homolog subfamily C GRV2 |
| 171 | Cs4g_pb020230 | chr4:21183340 | Promoter | chr8:2126956-2127275 | DNA/hat | Uncharacterized protein |
| 172 | Cs4g_pb020270 | chr4:21154403 | 3'UTR | chrUn:13606376-13607361 | LTR/Gypsy | Syntaxin-32 |
| 173 | Cs4g_pb020770 | chr4:27190555 | Promoter | chr7:10250532-10251237 | DNA/PIF-Harbinger | Auxin-responsive protein SAUR72 |
| 174 | Cs4g_pb021500 | chr4:26635732 | Promoter | chr1:25558503-25558816 | DNA/hat | Protein ENHANCED DISEASE RESISTANCE 2-like |
| 175 | Cs4g_pb021640 | chr4:26516160 | 3'UTR | chr7:14289642-14290187 | LINE/L1 | Uncharacterized protein |
| 176 | Cs4g_pb021650 | chr4:26516160 | Promoter | chr7:14289642-14290187 | LINE/L1 | Zinc finger CCCH domain-containing protein 18 |
| 177 | Cs4g_pb022200 | chr4:26081653 | Promoter | chr5:19347692-19348108 | LTR/Copia | Homogentisate geranylgeranyltransferase, chloroplastic |
| 178 | Cs4g_pb022280 | chr4:25975777 | Promoter | chrUn:10975834-10976035 | LTR/Copia | Uncharacterized protein |
| 179 | Cs4g_pb022310 | chr4:25945288 | 5'UTR | chr5:11043559-11044183 | LTR/Gypsy | Homeobox protein knotted-1-like 3 |
| 180 | Cs4g_pb022990 | chr4:25472912 | 3'UTR | chr4:5337663-5338217 | LINE/L1 | LysM domain receptor-like kinase 3 |
| 181 | Cs4g_pb023060 | chr4:25418929 | Promoter | chr4:25433884-25434232 | LTR/Copia | Adenine nucleotide transporter BT1, chloroplastic/mitochondrial |
| 182 | Cs4g_pb023710 | chr4:24863314 | Promoter | chr9:14061748-14062086 | DNA/hat | Serine/threonine protein phosphatase 2A 57 kDa regulatory subunit B' iota isoform |
| 183 | Cs4g_pb024340 | chr4:24361050 | Promoter | chr5:23584857-23588361 | DNA/hAT-Ac | Uncharacterized protein |
| 184 | Cs4g_pb024420 | chr4:24304034 | 5'UTR | chr5:12191333-12192319 | LTR/Gypsy | Aquaporin TIP4-1 |
| 185 | Cs4g_pb024530 | chr4:24163653 | Promoter | chr9:19347923-19348124 | LTR/Copia | Carotenoid cleavage dioxygenase 8 homolog B, chloroplastic |
| 186 | Cs4g_pb024830 | chr4:23885024 | Intron | chrUn:40035080-40035298 | LTR/Gypsy | Histidine-containing phosphotransfer protein 4 |
| 187 | Cs4g_pb025000 | chr4:23740640 | Promoter | chr5:33424284-33424998 | LTR/Copia | RNA polymerase sigma factor sigC |
| 188 | Cs4g_pb025120 | chr4:23633349 | Intron | chr9:19347923-19348124 | LTR/Copia | Phosphorylated adapter RNA export protein, RNA-binding domain |
| 189 | Cs4g_pb025600 | chr4:23181730 | Promoter | chr5:23584857-23588361 | DNA/hAT-Ac | Uncharacterized protein |
| 190 | Cs4g_pb025750 | chr4:23052527 | 3'UTR | chr9:19347923-19348124 | LTR/Copia | Blue copper protein |
| 191 | Cs4g_pb025900 | chr4:22903582 | Promoter | chr8:427180-427597 | LTR/Copia | EIN3-binding F-box protein 1 |
| 192 | Cs4g_pb026070 | chr4:22757681 | Promoter | chr8:2126956-2127275 | DNA/hat | 2-alkenal reductase (NADP(+)-dependent) |
| 193 | Cs5g_pb000090 | chr5:61918 | Promoter | chr2:4091426-4091627 | LTR/Copia | Tubulin-folding cofactor D |
| 194 | Cs5g_pb000610 | chr5:382223 | 5'UTR | chrUn:63397736-63410092 | rRNA | Probable carboxylesterase 8 |
| 195 | Cs5g_pb000850 | chr5:546931 | 3'UTR | chr5:37934017-37934977 | DNA/hAT-Ac | E3 ubiquitin-protein ligase ATL59 |
| 196 | Cs5g_pb001960 | chr5:1227771 | Promoter | chr5:23584857-23588361 | DNA/hAT-Ac | Protein REVEILLE 3 |
| 197 | Cs5g_pb002390 | chr5:1495528 | Promoter | chr5:23584857-23588361 | DNA/hAT-Ac | Ethylene-responsive transcription factor CRF5 |
| 198 | Cs5g_pb003090 | chr5:11577219 | Intron | chr1:20360574-20361197 | LTR/Gypsy | Actin-related protein 4 |
| 199 | Cs5g_pb003940 | chr5:10664391 | Promoter | chr2:9305349-9306046 | LTR/Copia | Isoaspartyl peptidase/L-asparaginase (Fragment) |
| 200 | Cs5g_pb004470 | chr5:10219103 | Promoter | chr1:25558503-25558816 | DNA/hat | Histone deacetylase 5 |
| 201 | Cs5g_pb004480 | chr5:10219103 | Intron | chr1:25558503-25558816 | DNA/hat | Uncharacterized protein |
| 202 | Cs5g_pb004780 | chr5:9917086 | Promoter | chr4:7022365-7022588 | LTR/Gypsy | Zinc finger protein 346 |
| 203 | Cs5g_pb005430 | chr5:9384711 | 3'UTR | chr2:1315340-1315563 | LTR/Gypsy | Geranylgeranyl diphosphate reductase, chloroplastic |
| 204 | Cs5g_pb005440 | chr5:9384711 | Promoter | chr2:1315340-1315563 | LTR/Gypsy | Malate dehydrogenase, chloroplastic |
| 205 | Cs5g_pb005460 | chr5:9372496 | 5'UTR | chr3:12119816-12120757 | DNA/hAT-Ac | Protein DCL, chloroplastic |
| 206 | Cs5g_pb006150 | chr5:8794601 | Promoter | chr3:5114099-5115084 | LTR/Gypsy | Uncharacterized protein |
| 207 | Cs5g_pb006880 | chr5:8258815 | Promoter | chr4:7022365-7022588 | LTR/Gypsy | MYB-LIKE DNA-BINDING PROTEIN MYB;MYB DNA BINDING / TRANSCRIPTION FACTOR |
| 208 | Cs5g_pb007310 | chr5:7880528 | Promoter | chr5:23584857-23588361 | DNA/hAT-Ac | L-lactate dehydrogenase A |
| 209 | Cs5g_pb007960 | chr5:7375164 | Promoter | chr2:3259240-3259574 | DNA/hat | Nudix hydrolase 8 |
| 210 | Cs5g_pb008070 | chr5:7263367 | Intron | chr4:10656535-10657679 | LINE/L1 | Ubiquitin carboxyl-terminal hydrolase isozyme L3 |
| 211 | Cs5g_pb009260 | chr5:6303994 | CDS | chr1:29026823-29032404 | LINE/L1 | Beta-glucosidase 24 |
| 212 | Cs5g_pb009380 | chr5:6228711 | Promoter | chr5:23584857-23588361 | DNA/hAT-Ac | Transcription factor GTE10 |
| 213 | Cs5g_pb009760 | chr5:5935100 | Promoter | chr8:7709624-7710250 | LTR/Gypsy | F-box/WD repeat-containing protein 11 |
| 214 | Cs5g_pb009840 | chr5:5871671 | Intron | chr1:28630136-28630470 | DNA/hat | Proline transporter 1 |
| 215 | Cs5g_pb010020 | chr5:5757594 | Promoter | chr9:11234853-11235727 | DNA/hAT-Ac | Uncharacterized protein |
| 216 | Cs5g_pb010030 | chr5:5757594 | Promoter | chr9:11234853-11235727 | DNA/hAT-Ac | 50S ribosomal protein L19, chloroplastic |
| 217 | Cs5g_pb010340 | chr5:5565433 | 5'UTR | chr5:37121890-37122113 | LTR/Gypsy | Oleosin 5 |
| 218 | Cs5g_pb010430 | chr5:5473736 | Promoter | chrUn:6353054-6353246 | DNA | 14-3-3 protein 10 |
| 219 | Cs5g_pb010440 | chr5:5473736 | Promoter | chrUn:6353054-6353246 | DNA | Probable LRR receptor-like serine/threonine-protein kinase At4g26540 |
| 220 | Cs5g_pb010590 | chr5:5360718 | Promoter | chr2:31454573-31454912 | DNA/hat | Diphthine methyltransferase |
| 221 | Cs5g_pb011050 | chr5:4983199 | Promoter | chr7:12215654-12216661 | DNA/hAT-Ac | NHL REPEAT-CONTAINING PROTEIN |
| 222 | Cs5g_pb011140 | chr5:4927620 | CDS | chrUn:66495119-66495479 | LTR/Copia | TRANSCRIPTION ELONGATION FACTOR B POLYPEPTIDE 3;ELONGIN-A-RELATED |
| 223 | Cs5g_pb011180 | chr5:4890491 | Promoter | chr5:14809407-14809738 | DNA/hat | LIM domain-containing protein WLIM1 |
| 224 | Cs5g_pb011190 | chr5:4890491 | Promoter | chr5:14809407-14809738 | DNA/hat | Uncharacterized protein At5g50100, mitochondrial |
| 225 | Cs5g_pb011810 | chr5:4510952 | 5'UTR | chr5:23584857-23588361 | DNA/hAT-Ac | HIPL1 protein; soluble quinoprotein glucose dehydrogenase |
| 226 | Cs5g_pb012110 | chr5:4276564 | Promoter | chr1:1884814-1885000 | LTR/Copia | Nuclear ribonuclease Z |
| 227 | Cs5g_pb012270 | chr5:4162235 | Promoter | chr1:25695620-25695957 | LTR/Copia | Probable glutathione peroxidase 8 |
| 228 | Cs5g_pb012720 | chr5:3886540 | Promoter | chr2:4091426-4091627 | LTR/Copia | Uncharacterized protein |
| 229 | Cs5g_pb013440 | chr5:3343525 | Promoter | chr4:20864036-20864797 | LTR/Gypsy | Potassium transporter 3 |
| 230 | Cs5g_pb013520 | chr5:3278101 | Promoter | chr5:23584857-23588361 | DNA/hAT-Ac | Cation/H(+) antiporter 18 |
| 231 | Cs5g_pb014800 | chr5:2479884 | Promoter | chr1:25558503-25558816 | DNA/hat | Cytochrome P450 78A3 |
| 232 | Cs5g_pb014980 | chr5:2280919 | Promoter | chr3:7429252-7429453 | LTR/Copia | Glucan endo-1,3-beta-glucosidase 11 |
| 233 | Cs5g_pb015770 | chr5:12334358 | CDS | chr5:12332782-12334333 | LTR/Copia | Epidermis-specific secreted glycoprotein EP1 |
| 234 | Cs5g_pb015990 | chr5:12603105 | Promoter | chr1:11475268-11475894 | LTR/Gypsy | 50S ribosomal protein L9 |
| 235 | Cs5g_pb016050 | chr5:12661700 | Promoter | chr4:7519944-7520163 | LTR/Gypsy | Probable LRR receptor-like serine/threonine-protein kinase At3g47570 |
| 236 | Cs5g_pb018450 | chr5:18129837 | Intron | chrUn:31294884-31295662 | LTR/Gypsy | Cytochrome P450 CYP72A219 |
| 237 | Cs5g_pb019970 | chr5:19369221 | 5'UTR | chr9:2919498-2919721 | LTR/Gypsy | StAR-related lipid transfer protein 7, mitochondrial |
| 238 | Cs5g_pb021400 | chr5:23562037 | Promoter | chr2:26223432-26223849 | LTR/Copia | Mitochodrial transcription termination factor-related |
| 239 | Cs5g_pb022550 | chr5:25606895 | Intron | chr2:23853823-23855223 | LINE/L1 | Uncharacterized protein |
| 240 | Cs5g_pb022950 | chr5:26192517 | Promoter | chr3:25620886-25621580 | LTR/Copia | Uncharacterized protein |
| 241 | Cs5g_pb023290 | chr5:26567110 | Promoter | chr8:427180-427597 | LTR/Copia | SERINE/THREONINE-PROTEIN KINASE;CALCIUM-BINDING EF HAND FAMILY PROTEIN |
| 242 | Cs5g_pb023390 | chr5:26759303 | Intron | chrUn:6475682-6476396 | LTR/Copia | RNA exonuclease 4 |
| 243 | Cs5g_pb024020 | chr5:27745369 | Promoter | chr4:15713071-15713272 | LTR/Copia | Uncharacterized protein |
| 244 | Cs5g_pb024080 | chr5:27629913 | Promoter | chr3:24226909-24227130 | LTR/Gypsy | Uncharacterized protein |
| 245 | Cs5g_pb026320 | chr5:30491702 | Promoter | chr3:1635512-1635735 | LTR/Gypsy | Probable phosphoinositide phosphatase SAC9 |
| 246 | Cs5g_pb026480 | chr5:30191290 | Promoter | chr9:23735550-23735751 | LTR/Copia | Beta-hexosaminidase 2 |
| 247 | Cs5g_pb027040 | chr5:29200076 | CDS | chr6:10277515-10280251 | LTR/Copia | Probable LRR receptor-like serine/threonine-protein kinase At4g08850 |
| 248 | Cs5g_pb028530 | chr5:34201220 | Promoter | chr2:4110056-4110417 | LTR/Copia | Clathrin interactor EPSIN 2 |
| 249 | Cs5g_pb030610 | chr5:36571425 | Promoter | chr1:25558503-25558816 | DNA/hat | 5'-adenylylsulfate reductase-like 5 |
| 250 | Cs5g_pb030620 | chr5:36571425 | Promoter | chr1:25558503-25558816 | DNA/hat | Eukaryotic translation initiation factor NCBP |
| 251 | Cs5g_pb031330 | chr5:37151857 | Promoter | chr7:19879771-19880396 | LTR/Gypsy | Uncharacterized protein |
| 252 | Cs5g_pb032720 | chr5:38191578 | Intron | chrUn:55617636-55618629 | DNA/hAT-Ac | Probable leucine-rich repeat receptor-like protein kinase At1g35710 |
| 253 | Cs5g_pb033700 | chr5:38909817 | Promoter | chr6:15570347-15570769 | LINE/L1 | 40S ribosomal protein S15 |
| 254 | Cs6g_pb000200 | chr6:2040679 | Promoter | chr3:25442134-25442764 | LTR/Gypsy | Uncharacterized PKHD-type hydroxylase At1g22950 |
| 255 | Cs6g_pb001150 | chr6:2842008 | Promoter | chr4:26860182-26860450 | DNA/hat | Mitogen-activated protein kinase-binding protein 1 |
| 256 | Cs6g_pb001640 | chr6:3753315 | Promoter | chr6:3753219-3753373 | DNA/hAT-Ac | Ribosome production factor 2 homolog |
| 257 | Cs6g_pb001650 | chr6:3753315 | Promoter | chr6:3753219-3753373 | DNA/hAT-Ac | Putative membrane-bound O-acyltransferase C24H6.01c |
| 258 | Cs6g_pb001680 | chr6:3780113 | Promoter | chr4:12449481-12449684 | LTR/Copia | Protein NRT1/ PTR FAMILY 4.3 |
| 259 | Cs6g_pb001730 | chr6:3871477 | Promoter | chr5:28485655-28486340 | LTR/Gypsy | Transcription factor PIF3 |
| 260 | Cs6g_pb003810 | chr6:7465971 | Promoter | chr2:19754661-19755285 | LTR/Gypsy | Uncharacterized protein |
| 261 | Cs6g_pb004470 | chr6:8464207 | Promoter | chr6:4723748-4724466 | LINE/L2 | Protein IQ-DOMAIN 1 |
| 262 | Cs6g_pb004690 | chr6:8869636 | 5'UTR | chr3:32153298-32154285 | LTR/Gypsy | 60S ribosomal protein L4 |
| 263 | Cs6g_pb005460 | chr6:9845800 | CDS | chr3:25620886-25621580 | LTR/Copia | Uncharacterized protein |
| 264 | Cs6g_pb007070 | chr6:12091556 | 3'UTR | chr9:9262240-9262461 | LTR/Gypsy | Putative receptor protein kinase ZmPK1 |
| 265 | Cs6g_pb007080 | chr6:12091556 | Promoter | chr9:9262240-9262461 | LTR/Gypsy | Protein RETICULATA |
| 266 | Cs6g_pb007500 | chr6:12421550 | Intron | chr1:11156442-11157335 | LTR/Copia | Transcription termination/antitermination protein NusG |
| 267 | Cs6g_pb007830 | chr6:19913336 | Intron | chr4:7022365-7022588 | LTR/Gypsy | Uncharacterized protein |
| 268 | Cs6g_pb008860 | chr6:19178536 | Intron | chrUn:59135082-59139325 | LTR/Gypsy | E3 ubiquitin-protein ligase SINA-like 10 |
| 269 | Cs6g_pb009380 | chr6:18821249 | Promoter | chrUn:58189872-58195717 | LINE/L1 | Probable inactive receptor kinase At1g48480 |
| 270 | Cs6g_pb010380 | chr6:18165273 | Promoter | chr3:24749743-24750610 | LTR/Copia | Putative F-box protein At5g50220 |
| 271 | Cs6g_pb010470 | chr6:18125250 | Promoter | chr5:21658030-21658231 | LTR/Copia | E3 ubiquitin-protein ligase RING1-like |
| 272 | Cs6g_pb011430 | chr6:17408308 | Intron | chr3:26455927-26456128 | LTR/Copia | 1,4-alpha-glucan-branching enzyme 1, chloroplastic/amyloplastic |
| 273 | Cs6g_pb011620 | chr6:17218980 | Promoter | chr1:14635556-14635901 | DNA/hat | Probable F-box protein At1g60180 |
|  |  | chr6:17219950 | Promoter | chr4:7022365-7022588 | LTR/Gypsy |  |
| 274 | Cs6g_pb012680 | chr6:16511326 | Promoter | chr5:23584857-23588361 | DNA/hAT-Ac | IAA-amino acid hydrolase ILR1-like 7 |
| 275 | Cs6g_pb013880 | chr6:15573670 | Promoter | chr9:16763348-16763566 | LTR/Gypsy | Protein ROOT PRIMORDIUM DEFECTIVE 1 |
| 276 | Cs6g_pb013890 | chr6:15573670 | Promoter | chr9:16763348-16763566 | LTR/Gypsy | Uncharacterized protein |
| 277 | Cs6g_pb016860 | chr6:13332388 | Promoter | chrUn:55649165-55649386 | LTR/Gypsy | CASP-like protein 4B1 |
| 278 | Cs6g_pb016920 | chr6:13280894 | Promoter | chr1:21853240-21858210 | LTR/Copia | Cation/H(+) antiporter 15 |
| 279 | Cs6g_pb018140 | chr6:20401447 | Promoter | chr1:11475268-11475894 | LTR/Gypsy | UDP-arabinopyranose mutase 1 |
| 280 | Cs6g_pb018170 | chr6:20430763 | Intron | chrUn:5775158-5775476 | LTR/Copia | Developmental protein SEPALLATA 2 |
| 281 | Cs7g_pb001220 | chr7:2596106 | Promoter | chr2:32483531-32483868 | DNA/hat | Uncharacterized protein |
| 282 | Cs7g_pb001870 | chr7:2186709 | Promoter | chr8:12271168-12271387 | LTR/Gypsy | Alpha-glucan water dikinase, chloroplastic |
| 283 | Cs7g_pb002250 | chr7:1948154 | Promoter | chr3:23579634-23580032 | LINE/L1 | Proline-rich receptor-like protein kinase PERK13 |
| 284 | Cs7g_pb002260 | chr7:1948154 | Promoter | chr3:23579634-23580032 | LINE/L1 | Probable inactive poly [ADP-ribose] polymerase SRO2 |
| 285 | Cs7g_pb002970 | chr7:1420123 | Promoter | chr5:24146968-24147187 | LTR/Gypsy | Two-component response regulator ARR3 |
| 286 | Cs7g_pb004160 | chr7:601513 | Promoter | chrUn:55649165-55649386 | LTR/Gypsy | Cyclic dof factor 2 |
| 287 | Cs7g_pb005350 | chr7:3916739 | Promoter | chr5:23584857-23588361 | DNA/hAT-Ac | Copper methylamine oxidase |
| 288 | Cs7g_pb007240 | chr7:5223462 | 5'UTR | chrUn:12471944-12472254 | DNA/hat | Probable DNA primase large subunit |
| 289 | Cs7g_pb007360 | chr7:5303427 | Promoter | chr1:25558503-25558816 | DNA/hat | Glucose-6-phosphate 1-dehydrogenase, chloroplastic |
| 290 | Cs7g_pb008390 | chr7:6044522 | Promoter | chr4:7022365-7022588 | LTR/Gypsy | Non-specific lipid-transfer protein A |
| 291 | Cs7g_pb008630 | chr7:6261119 | 3'UTR | chr3:20206379-20206686 | DNA/hat | Protein STRICTOSIDINE SYNTHASE-LIKE 10 |
| 292 | Cs7g_pb008840 | chr7:6478138 | Promoter | chr5:23584857-23588361 | DNA/hAT-Ac | Phosphoenolpyruvate carboxylase 4 |
| 293 | Cs7g_pb009410 | chr7:6937864 | Intron | chr6:613256-613797 | DNA/MULE-MuDR | Protein NRT1/ PTR FAMILY 3.1 |
| 294 | Cs7g_pb009420 | chr7:6963041 | Promoter | chr2:19182090-19182935 | LTR/Copia | Ubiquitin receptor RAD23c |
| 295 | Cs7g_pb010220 | chr7:7583949 | Promoter | chrUn:55649165-55649386 | LTR/Gypsy | Sacsin |
| 296 | Cs7g_pb010230 | chr7:7583949 | Promoter | chrUn:55649165-55649386 | LTR/Gypsy | Endoglucanase 5 |
| 297 | Cs7g_pb010690 | chr7:8272084 | Promoter | chr3:36508274-36508625 | DNA/hat | Mitochondrial substrate carrier family protein W |
| 298 | Cs7g_pb011480 | chr7:8944829 | Promoter | chr4:7022365-7022588 | LTR/Gypsy | Cytochrome P450 89A2 |
| 299 | Cs7g_pb011870 | chr7:9381825 | Promoter | chr3:22109696-22110511 | LTR/Copia | Translation initiation factor IF-2 |
| 300 | Cs7g_pb011880 | chr7:9381825 | Promoter | chr3:22109696-22110511 | LTR/Copia | Uncharacterized protein |
| 301 | Cs7g_pb012020 | chr7:9506975 | 3'UTR | chr3:2481773-2482761 | LTR/Gypsy | CRAL-TRIO domain-containing protein C23B6.04c |
| 302 | Cs7g_pb012030 | chr7:9506975 | Promoter | chr3:2481773-2482761 | LTR/Gypsy | Uncharacterized protein |
| 303 | Cs7g_pb013560 | chr7:11276689 | Promoter | chr4:7022365-7022588 | LTR/Gypsy | Uncharacterized protein |
| 304 | Cs7g_pb013570 | chr7:11276689 | Promoter | chr4:7022365-7022588 | LTR/Gypsy | GDSL esterase/lipase At4g10955 |
| 305 | Cs7g_pb013700 | chr7:10972758 | Intron | chr9:19577651-19578574 | DNA/hAT-Ac | Callose synthase 7 |
| 306 | Cs7g_pb013910 | chr7:11982324 | Promoter | chr5:12566696-12567151 | LTR/Copia | TBCC domain-containing protein 1 |
| 307 | Cs7g_pb014290 | chr7:12299306 | Promoter | chrUn:41732936-41733902 | DNA/hAT-Ac | Probable beta-1,4-xylosyltransferase IRX10L |
| 308 | Cs7g_pb014600 | chr7:12641543 | Intron | chr9:24508786-24508987 | LTR/Copia | ADP-ribosylation factor GTPase-activating protein AGD1 |
| 309 | Cs7g_pb015290 | chr7:14789398 | Promoter | chrUn:4181728-4181950 | LTR/Gypsy | mRNA cap guanine-N7 methyltransferase 2 |
| 310 | Cs7g_pb015300 | chr7:14789398 | Promoter | chrUn:4181728-4181950 | LTR/Gypsy | Aspartic proteinase nepenthesin-2 |
| 311 | Cs7g_pb015850 | chr7:13897516 | Promoter | chr9:14875954-14876577 | LTR/Gypsy | Ethylene-responsive transcription factor ERF114 |
| 312 | Cs7g_pb016220 | chr7:13271214 | Promoter | chr1:24345137-24345323 | LTR/Copia | Ribose-phosphate pyrophosphokinase 1, chloroplastic |
| 313 | Cs7g_pb016750 | chr7:15767267 | Intron | chrUn:57378204-57385431 | LTR/Caulimovirus | NAD-dependent malic enzyme 62 kDa isoform, mitochondrial |
| 314 | Cs7g_pb017670 | chr7:18396566 | Promoter | chr5:27316134-27316758 | LTR/Gypsy | Exocyst complex component EXO70A1 |
| 315 | Cs7g_pb017890 | chr7:17761300 | Promoter | chr5:34252471-34252807 | DNA/hat | Vesicle-associated membrane protein 727 |
| 316 | Cs7g_pb019640 | chr7:26333952 | Promoter | chr5:23584857-23588361 | DNA/hAT-Ac | Zinc finger protein ZAT11 |
| 317 | Cs7g_pb019910 | chr7:26163065 | Promoter | chr7:26162589-26163211 | Simple_repeat | Retrovirus-related Pol polyprotein from transposon TNT 1-94 |
| 318 | Cs7g_pb020790 | chr7:25482645 | Promoter | chr4:7022365-7022588 | LTR/Gypsy | CTP synthase |
| 319 | Cs7g_pb021110 | chr7:25202783 | Promoter | chr5:23584857-23588361 | DNA/hAT-Ac | Patatin-like protein 6 |
| 320 | Cs7g_pb022340 | chr7:27379064 | Promoter | chr2:32319598-32319917 | DNA/hat | Uncharacterized protein |
| 321 | Cs7g_pb022380 | chr7:27436011 | Promoter | chr5:20027964-20028362 | LINE/L1 | Mediator of RNA polymerase II transcription subunit 13 |
| 322 | Cs7g_pb022390 | chr7:27436011 | Promoter | chr5:20027964-20028362 | LINE/L1 | Elongation factor P |
| 323 | Cs7g_pb023660 | chr7:28352314 | Promoter | chrUn:55370953-55371153 | LTR/Copia | Putative E3 ubiquitin-protein ligase LIN-1 |
| 324 | Cs7g_pb024250 | chr7:28845581 | Promoter | chr5:13687385-13687608 | LTR/Gypsy | DNA topoisomerase 6 subunit B |
| 325 | Cs7g_pb025010 | chr7:29414453 | Promoter | chr5:25573943-25574166 | LTR/Gypsy | Probable chalcone--flavonone isomerase 3 |
| 326 | Cs7g_pb025100 | chr7:29487746 | Promoter | chr3:27541617-27543925 | LTR/Gypsy | Metallothionein-like protein type 2 |
| 327 | Cs7g_pb025630 | chr7:29895150 | Promoter | chr7:22007797-22008551 | DNA/MULE-MuDR | PRA1 family protein B4 |
| 328 | Cs7g_pb025740 | chr7:30037407 | Intron | chrUn:55649165-55649386 | LTR/Gypsy | SNF2 domain-containing protein CLASSY 2 |
| 329 | Cs7g_pb025760 | chr7:30037407 | Promoter | chrUn:55649165-55649386 | LTR/Gypsy | Protein trichome birefringence-like 33 |
| 330 | Cs7g_pb025770 | chr7:30037407 | Promoter | chrUn:55649165-55649386 | LTR/Gypsy | Uncharacterized protein |
| 331 | Cs7g_pb026120 | chr7:30276177 | Intron | chr1:28736381-28737104 | LINE/L2 | GDP-Man:Man(3)GlcNAc(2)-PP-Dol alpha-1,2-mannosyltransferase |
|  |  | chr7:30277031 | Promoter | chr9:7987743-7988104 | LTR/Copia |  |
| 332 | Cs8g_pb001020 | chr8:593833 | Intron | chr8:593666-593974 | DNA/hat | Lysine histidine transporter-like 8 |
| 333 | Cs8g_pb001260 | chr8:4251530 | Promoter | chr5:23584857-23588361 | DNA/hAT-Ac | Peptidyl-prolyl cis-trans isomerase |
| 334 | Cs8g_pb001280 | chr8:4251530 | 3'UTR | chr5:23584857-23588361 | DNA/hAT-Ac | Nucleoporin NUP188 homolog |
| 335 | Cs8g_pb001410 | chr8:4103062 | Promoter | chr2:26223432-26223849 | LTR/Copia | Pentatricopeptide repeat-containing protein At2g15980 |
| 336 | Cs8g_pb002260 | chr8:3447182 | CDS | chr5:14968710-14969583 | DNA/hAT-Ac | Probable carboxylesterase 2 |
| 337 | Cs8g_pb002270 | chr8:3447182 | Promoter | chr5:14968710-14969583 | DNA/hAT-Ac | Probable carboxylesterase 2 |
| 338 | Cs8g_pb003020 | chr8:2846474 | Intron | chr3:30492871-30493743 | LTR/Copia | O-acyltransferase WSD1 |
| 339 | Cs8g_pb003740 | chr8:2344310 | Promoter | chr4:25893470-25894118 | LTR/Copia | Pumilio homolog 12 |
| 340 | Cs8g_pb006070 | chr8:675160 | Promoter | chr2:16368317-16369042 | LINE/L2 | Expansin-like A3 |
| 341 | Cs8g_pb006760 | chr8:7507197 | Promoter | chr8:2126956-2127275 | DNA/hat | Auxin-induced protein 15A |
| 342 | Cs8g_pb006910 | chr8:7114586 | Intron | chrUn:66317987-66323627 | LTR/Gypsy | Disease resistance protein RPS5 |
| 343 | Cs8g_pb007670 | chr8:6217198 | Promoter | chr3:29467697-29468005 | DNA/hat | Uncharacterized protein |
| 344 | Cs8g_pb008890 | chr8:5041034 | Promoter | chr4:9626661-9626838 | DNA/hat | Organ-specific protein S2 |
|  |  | chr8:5042450 | 5'UTR | chr5:24446391-24447083 | LTR/Copia |  |
| 345 | Cs8g_pb008900 | chr8:5041034 | Promoter | chr4:9626661-9626838 | DNA/hat | BURP domain-containing protein 17 |
| 346 | Cs8g_pb009270 | chr8:4622903 | Promoter | chrUn:45825133-45827539 | LINE/L1 | Dolichyl-diphosphooligosaccharide--protein glycosyltransferase 48 kDa subunit |
| 347 | Cs8g_pb010280 | chr8:10279666 | Promoter | chr7:19998577-19998952 | LTR/Copia | Xyloglucan galactosyltransferase KATAMARI1 |
| 348 | Cs8g_pb010520 | chr8:10799589 | 5'UTR | chr5:23584857-23588361 | DNA/hAT-Ac | Uncharacterized protein |
| 349 | Cs8g_pb011520 | chr8:14851427 | 5'UTR | chr3:345421-346408 | LTR/Gypsy | B3 domain-containing transcription factor VRN1 |
| 350 | Cs8g_pb011570 | chr8:14810688 | Promoter | chr7:4595743-4599948 | LINE/L1 | Putative phosphatidylglycerol/phosphatidylinositol transfer protein DDB_G0282179 |
| 351 | Cs8g_pb011750 | chr8:14607603 | Promoter | chrUn:10889246-10889436 | DNA | Probable carotenoid cleavage dioxygenase 4, chloroplastic |
| 352 | Cs8g_pb011760 | chr8:14575363 | Intron | chrUn:5456314-5456575 | DNA/hat | Phragmoplast orienting kinesin-1 |
| 353 | Cs8g_pb012060 | chr8:13915161 | Promoter | chrUn:66495119-66495479 | LTR/Copia | Wound-induced protein WIN2 |
| 354 | Cs8g_pb013530 | chr8:16629439 | Promoter | chr5:36791354-36792062 | LINE/L2 | Aluminum-activated malate transporter 7 |
| 355 | Cs8g_pb014230 | chr8:15258013 | Promoter | chr5:38049175-38049376 | LTR/Copia | Uncharacterized protein |
| 356 | Cs8g_pb014240 | chr8:15258013 | Promoter | chr5:38049175-38049376 | LTR/Copia | Uncharacterized protein |
| 357 | Cs8g_pb014350 | chr8:19173320 | 3'UTR | chr2:10190525-10191139 | LTR/Copia | Putative SWI/SNF-related matrix-associated actin-dependent regulator of chromatin subfamily A member 3-like 2 |
| 358 | Cs8g_pb016610 | chr8:23023182 | Promoter | chr2:26516157-26516830 | LTR/Gypsy | Sodium-dependent phosphate transport protein 1, chloroplastic |
| 359 | Cs8g_pb016890 | chr8:22838978 | Promoter | chr3:11790616-11791285 | Simple_repeat | Neutral ceramidase |
| 360 | Cs8g_pb016900 | chr8:22838978 | Intron | chr3:11790616-11791285 | Simple_repeat | Uncharacterized protein |
| 361 | Cs8g_pb016910 | chr8:22838978 | Promoter | chr3:11790616-11791285 | Simple_repeat | TRAF-like;NULL;Zinc finger, RING-type;Zinc finger, RING-type, conserved site;Zinc finger, RING/FYVE/PHD-type |
| 362 | Cs8g_pb017450 | chr8:22602770 | Promoter | chr8:3933315-3934300 | LTR/Gypsy | Senescence-associated protein 13 |
| 363 | Cs8g_pb018900 | chr8:21682529 | Promoter | chrUn:29926545-29927248 | LINE/L2 | Ankyrin repeat-containing protein At5g02620 |
| 364 | Cs8g_pb019490 | chr8:21204035 | Promoter | chr2:31454573-31454912 | DNA/hat | Tetraspanin-3 |
| 365 | Cs8g_pb020200 | chr8:20742693 | Promoter | chr4:7022365-7022588 | LTR/Gypsy | Ankyrin repeat-containing protein At3g12360 |
| 366 | Cs8g_pb021140 | chr8:19943175 | Promoter | chr1:19003846-19004564 | DNA/PIF-Harbinger | Transcription factor FER-LIKE IRON DEFICIENCY-INDUCED TRANSCRIPTION FACTOR |
| 367 | Cs9g_pb000010 | chr9:1872 | Promoter | chr3:33264167-33264831 | DNA/hat | Uncharacterized protein |
| 368 | Cs9g_pb000820 | chr9:558385 | Promoter | chr5:21354727-21354950 | LTR/Gypsy | F-box |
| 369 | Cs9g_pb001630 | chr9:1072875 | Promoter | chr3:36508274-36508625 | DNA/hat | 3-oxo-Delta(4,5)-steroid 5-beta-reductase |
| 370 | Cs9g_pb001640 | chr9:1072875 | Promoter | chr3:36508274-36508625 | DNA/hat | Putative pentatricopeptide repeat-containing protein At3g16890, mitochondrial |
| 371 | Cs9g_pb001940 | chr9:1256643 | 5'UTR | chr1:25558503-25558816 | DNA/hat | Major allergen Pru ar 1 |
| 372 | Cs9g_pb002250 | chr9:1414605 | CDS | chr4:18670391-18671065 | LTR/Gypsy | Uncharacterized protein |
| 373 | Cs9g_pb002450 | chr9:1560255 | Promoter | chr9:18007128-18007349 | LTR/Gypsy | MATE efflux family protein 5 |
| 374 | Cs9g_pb002660 | chr9:1693874 | Promoter | chr5:17136054-17136414 | LTR/Copia | Translationally-controlled tumor protein homolog |
| 375 | Cs9g_pb003770 | chr9:2437465 | Promoter | chr2:3789754-3789940 | LTR/Copia | Putative E3 ubiquitin-protein ligase RF298 |
| 376 | Cs9g_pb003780 | chr9:2437465 | Promoter | chr2:3789754-3789940 | LTR/Copia | Uncharacterized protein |
| 377 | Cs9g_pb004070 | chr9:4672991 | Intron | chrUn:55903171-55903867 | LTR/Copia | 3-ketoacyl-CoA synthase 11 |
| 378 | Cs9g_pb004080 | chr9:4672991 | Promoter | chrUn:55903171-55903867 | LTR/Copia | Armadillo repeat-containing protein LFR |
| 379 | Cs9g_pb004130 | chr9:4591485 | Promoter | chr6:16501742-16501965 | LTR/Gypsy | Pyruvate kinase isozyme A, chloroplastic |
| 380 | Cs9g_pb004140 | chr9:4591485 | Promoter | chr6:16501742-16501965 | LTR/Gypsy | ADP-ribosylation factor-like protein 5 |
| 381 | Cs9g_pb004520 | chr9:4272419 | Promoter | chr8:9164058-9170547 | LTR/Gypsy | Uncharacterized protein |
| 382 | Cs9g_pb004590 | chr9:4208951 | Promoter | chr6:6552574-6552919 | LTR/Copia | Homocysteine S-methyltransferase 3 |
| 383 | Cs9g_pb004660 | chr9:4160874 | Promoter | chr2:7995292-7995510 | LTR/Gypsy | Uncharacterized protein |
| 384 | Cs9g_pb004670 | chr9:4160874 | Promoter | chr2:7995292-7995510 | LTR/Gypsy | Uncharacterized protein |
| 385 | Cs9g_pb004680 | chr9:4160874 | Promoter | chr2:7995292-7995510 | LTR/Gypsy | Vicilin GC72-A |
| 386 | Cs9g_pb004890 | chr9:4077325 | Promoter | chr2:17730617-17730820 | LTR/Copia | Folate-biopterin transporter 1, chloroplastic |
| 387 | Cs9g_pb004990 | chr9:4026381 | Promoter | chr5:25573943-25574166 | LTR/Gypsy | Pre-rRNA-processing protein TSR2 homolog |
| 388 | Cs9g_pb005000 | chr9:4026381 | Promoter | chr5:25573943-25574166 | LTR/Gypsy | Pentatricopeptide repeat-containing protein At4g14850 |
| 389 | Cs9g_pb005300 | chr9:3825924 | Promoter | chr5:23584857-23588361 | DNA/hAT-Ac | Primary amine oxidase |
| 390 | Cs9g_pb005310 | chr9:3810554 | Intron | chr8:16141344-16142185 | DNA/hAT-Ac | G-type lectin S-receptor-like serine/threonine-protein kinase RLK1 |
| 391 | Cs9g_pb005520 | chr9:3623940 | 3'UTR | chr6:9689944-9690854 | LTR/Copia | Probable membrane-associated kinase regulator 1 |
| 392 | Cs9g_pb005610 | chr9:3560601 | Promoter | chr2:3259240-3259574 | DNA/hat | 2-hydroxyisoflavanone dehydratase |
| 393 | Cs9g_pb006060 | chr9:3217163 | Intron | chrUn:63434163-63436303 | LTR/Gypsy | Probable serine incorporator |
| 394 | Cs9g_pb006080 | chr9:3217163 | Promoter | chrUn:63434163-63436303 | LTR/Gypsy | 60S RIBOSOMAL PROTEIN L34;STRUCTURAL CONSTITUENT OF RIBOSOME |
| 395 | Cs9g_pb007310 | chr9:5740253 | Intron | chr5:23584857-23588361 | DNA/hAT-Ac | Cellulose synthase-like protein G2 |
| 396 | Cs9g_pb007470 | chr9:5991981 | Promoter | chr9:11928341-11929033 | LTR/Copia | Zinc finger CCCH domain-containing protein 13 |
| 397 | Cs9g_pb007700 | chr9:6311953 | Promoter | chrUn:13606376-13607361 | LTR/Gypsy | Beta-amylase |
| 398 | Cs9g_pb009410 | chr9:9091897 | Intron | chr7:22354490-22357969 | LTR/Gypsy | Tuberculostearic acid methyltransferase UfaA1 |
| 399 | Cs9g_pb009910 | chr9:9764344 | Promoter | chrUn:14052933-14053134 | LTR/Copia | COP1-INTERACTING PROTEIN-RELATED |
| 400 | Cs9g_pb011210 | chr9:11364561 | Promoter | chr2:14602318-14603191 | DNA/hAT-Ac | RING-H2 finger protein ATL7 |
| 401 | Cs9g_pb012190 | chr9:12637921 | Promoter | chrUn:12176988-12177169 | DNA/hAT-Ac | Probable polygalacturonase At1g80170 |
| 402 | Cs9g_pb012400 | chr9:12864785 | Intron | chr9:12811690-12811980 | LTR/Copia | Probable disease resistance protein At5g63020 |
| 403 | Cs9g_pb013030 | chr9:13398589 | Intron | chr5:14809407-14809738 | DNA/hat | Pleiotropic drug resistance protein 1 |
|  |  | chr9:13400018 | Intron | chr2:22809695-22810113 | LTR/Copia |  |
| 404 | Cs9g_pb014070 | chr9:15335121 | Promoter | chr8:427180-427597 | LTR/Copia | NADPH-dependent thioredoxin reductase 3 |
| 405 | Cs9g_pb014190 | chr9:17044853 | Promoter | chr5:23584857-23588361 | DNA/hAT-Ac | Potassium channel SKOR |
| 406 | Cs9g_pb014270 | chr9:16855698 | Promoter | chr2:18785604-18786094 | DNA/MuLE-MuDR | Cysteine proteinases |
| 407 | Cs9g_pb014830 | chr9:15998673 | CDS | chr3:1635512-1635735 | LTR/Gypsy | Glucan endo-1,3-beta-glucosidase, basic isoform |
| 408 | Cs9g_pb015460 | chr9:20244108 | Promoter | chr4:7022365-7022588 | LTR/Gypsy | Mavicyanin |
| 409 | Cs9g_pb015720 | chr9:19954392 | Intron | chr4:26860182-26860450 | DNA/hat | LINE-1 retrotransposable element ORF2 protein |
| 410 | Cs9g_pb016220 | chr9:19463156 | Promoter | chr8:11070940-11074525 | DNA/hAT-Ac | Uncharacterized protein |
| 411 | Cs9g_pb017570 | chr9:20816207 | 5'UTR | chr9:7987743-7988104 | LTR/Copia | Probable serine/threonine protein kinase IRE |
| 412 | Cs9g_pb018280 | chr9:21867383 | Promoter | chr8:7996816-7997039 | LTR/Gypsy | Receptor-like serine/threonine-protein kinase At4g25390 |
| 413 | Cs9g_pb019200 | chr9:22606473 | Promoter | chr6:5594291-5594513 | LTR/Gypsy | Uncharacterized protein |
| 414 | Cs9g_pb019280 | chr9:22662492 | Promoter | chr1:25558503-25558816 | DNA/hat | Protein O-glucosyltransferase 1 |
| 415 | Cs9g_pb020010 | chr9:23654438 | Promoter | chr1:16775575-16776664 | LINE/L1 | Uncharacterized protein |
| 416 | Cs9g_pb020020 | chr9:23654438 | 5'UTR | chr1:16775575-16776664 | LINE/L1 | Phosphoglycerate kinase 2, chloroplastic |
| 417 | Cs9g_pb020220 | chr9:23805968 | Promoter | chr9:19076698-19077691 | DNA/hAT-Ac | Pectinesterase inhibitor |
| 418 | Cs9g_pb020530 | chr9:24064199 | Promoter | chr2:4110056-4110417 | LTR/Copia | Spermine synthase |

| **Table S7 Statistics of DNA methylation level in the materials used in this study.** | | | | | | | | | | | | | | | | | |
| --- | --- | --- | --- | --- | --- | --- | --- | --- | --- | --- | --- | --- | --- | --- | --- | --- | --- |
| **Material** | **Transposable Elements** | | | | | |  | **Protein coding Genes** | | | | | |  | **mCG (%)** | **CHG (%)** | **CHH (%)** |
|  | **TE region** | | | **Upstream 2kb** | | |  | **Gene Body** | | | **Upstream 2kb** | | |  |  |  |  |
|  | **mCG (%)** | **mCHG (%)** | **mCHH (%)** | **mCG (%)** | **mCHG (%)** | **mCHH (%)** |  | **mCG (%)** | **mCHG (%)** | **mCHH (%)** | **mCG (%)** | **mCHG (%)** | **mCHH (%)** |  |  |  |  |
| EX | 73.07 | 60.47 | 8.34 | 67.29 | 55.40 | 7.22 |  | 57.61 | 9.43 | 1.78 | 51.28 | 34.78 | 6.43 |  | 71.35 | 54.14 | 8.52 |
| NI | 77.60 | 67.73 | 10.67 | 52.08 | 37.69 | 5.66 |  | 42.18 | 11.08 | 1.86 | 38.63 | 24.80 | 4.79 |  | 53.21 | 28.51 | 4.60 |
| Di1 | 77.09 | 67.12 | 20.75 | 50.77 | 36.63 | 11.22 |  | 41.10 | 9.93 | 2.88 | 37.36 | 23.85 | 11.14 |  | 52.20 | 28.25 | 8.31 |
| Di2 | 79.54 | 67.21 | 22.67 | 54.10 | 37.91 | 12.61 |  | 42.55 | 9.95 | 3.18 | 40.17 | 24.45 | 12.05 |  | 55.28 | 29.33 | 9.64 |
| Tet | 80.35 | 65.48 | 14.90 | 56.40 | 38.54 | 8.42 |  | 45.65 | 11.02 | 2.61 | 42.36 | 24.87 | 6.92 |  | 57.74 | 30.28 | 7.27 |
| TetP | 62.62 | 46.87 | 15.35 | 47.82 | 34.69 | 11.25 |  | 44.07 | 5.05 | 2.16 | 30.74 | 19.44 | 10.49 |  | 46.22 | 26.17 | 8.72 |
| Hex | 81.46 | 64.69 | 11.09 | 59.52 | 40.92 | 7.10 |  | 43.92 | 12.70 | 2.51 | 43.98 | 26.45 | 5.23 |  | 61.47 | 34.83 | 7.28 |

| **Table S8. Hub genes that specifically expressed in the newly-induced callus.** | | | | | | | | | | | | | |
| --- | --- | --- | --- | --- | --- | --- | --- | --- | --- | --- | --- | --- | --- |
| **Gene ID** | **Normalized expression level (FPKM)** | | | | | | | | | | | | **Gene functional annotation** |
|  | **Leaf** | **Seed** | **Young fruit** | **Mature fruit** | **Early-stage ovule** | **Late-stage ovule** | **Newly-induced callus**  **(NI)** | **Diploid callus**  **(Di1)** | **Diploid callus**  **(Di2)** | **DH callus** | **Tetraploid callus**  **(Tet)** | **Hexaploid callus**  **(Hex)** |  |
| Cs1g_pb003640 | 0.00 | 0.00 | 0.00 | 0.00 | 0.00 | 0.04 | 6.32 | 0.04 | 0.00 | 0.25 | 0.70 | 0.49 |  |
| Cs1g_pb004710 | 0.00 | 0.00 | 0.00 | 0.00 | 0.00 | 0.00 | 5.85 | 0.00 | 0.00 | 0.00 | 0.00 | 0.00 |  |
| Cs1g_pb010390 | 0.11 | 0.43 | 0.00 | 0.15 | 0.11 | 0.00 | 4.42 | 0.07 | 0.13 | 0.15 | 0.08 | 0.00 | Beta-1,3-galactosyl-O-glycosyl-glycoprotein beta-1,6-N-acetylglucosaminyltransferase 7 |
| Cs1g_pb010710 | 0.00 | 0.00 | 0.00 | 0.00 | 0.00 | 0.00 | 5.19 | 0.04 | 0.02 | 0.78 | 0.53 | 0.11 |  |
| Cs1g_pb013660 | 0.00 | 0.00 | 0.00 | 0.00 | 0.00 | 0.00 | 12.97 | 0.00 | 0.79 | 0.00 | 1.05 | 0.00 |  |
| Cs1g_pb015410 | 0.00 | 0.00 | 0.00 | 0.00 | 0.00 | 0.00 | 1.02 | 0.00 | 0.00 | 0.00 | 0.00 | 0.00 |  |
| Cs1g_pb016730 | 0.00 | 0.00 | 0.00 | 0.00 | 0.00 | 0.00 | 4.09 | 0.00 | 0.34 | 0.00 | 0.00 | 0.00 |  |
| Cs1g_pb018710 | 6.22 | 1.19 | 3.12 | 0.04 | 3.66 | 3.23 | 94.41 | 6.18 | 8.11 | 20.80 | 6.57 | 9.52 | Calcium-transporting ATPase 2, plasma membrane-type; This magnesium-dependent enzyme catalyzes the hydrolysis of ATP coupled with the translocation of calcium from the cytosol into the endoplasmic reticulum. |
| Cs1g_pb018820 | 0.00 | 0.08 | 0.00 | 0.00 | 1.03 | 0.61 | 10.66 | 0.00 | 0.05 | 0.21 | 0.03 | 0.00 | Dehydrogenase/reductase SDR family member 7B |
| Cs1g_pb019490 | 0.00 | 0.00 | 0.00 | 0.00 | 0.00 | 0.00 | 3.11 | 0.07 | 0.04 | 0.51 | 0.15 | 0.54 | L-type lectin-domain containing receptor kinase IX.1; Promotes hydrogen peroxide H2O2 production and cell death. |
| Cs1g_pb021200 | 1.65 | 0.13 | 0.09 | 0.74 | 0.23 | 0.16 | 13.02 | 0.31 | 0.18 | 0.10 | 0.08 | 0.18 | Peroxidase 21; Removal of H2O2, oxidation of toxic reductants, biosynthesis and degradation of lignin, suberization, auxin catabolism, response to environmental stresses such as wounding, pathogen attack and oxidative stress. |
| Cs1g_pb022310 | 0.50 | 0.12 | 0.17 | 0.58 | 0.24 | 0.42 | 4.96 | 0.17 | 0.14 | 0.25 | 0.21 | 0.11 | Protein LYK5; May recognize microbe-derived N-acetylglucosamine (NAG)-containing ligands. |
| Cs1g_pb022900 | 2.07 | 0.00 | 0.00 | 0.00 | 0.00 | 0.00 | 63.05 | 0.00 | 0.00 | 6.47 | 4.24 | 3.32 | Protein RADIALIS-like 1; Assigned as a member of the MYB-related gene family, I-box-binding-like subfamily. |
| Cs1g_pb023510 | 0.00 | 0.08 | 0.16 | 0.00 | 0.36 | 0.12 | 8.58 | 1.17 | 1.43 | 0.36 | 0.12 | 0.09 | Purple acid phosphatase 10; Purple acid phosphatase 10 |
| Cs2g_pb001010 | 0.00 | 0.00 | 0.00 | 0.00 | 0.00 | 0.21 | 5.96 | 0.05 | 0.47 | 0.36 | 0.65 | 0.47 | Cell wall / vacuolar inhibitor of fructosidase 2; Inhibits fructosidases from both cell wall (cell wall invertase CWI) and vacuoles (vacuolar invertase VI). |
| Cs2g_pb003360 | 0.00 | 0.00 | 0.00 | 0.00 | 0.00 | 0.00 | 3.09 | 0.00 | 0.00 | 0.52 | 0.00 | 0.16 | Vacuolar iron transporter homolog 4; may be involved in the regulation of iron distribution throughout the plant. |
| Cs2g_pb004130 | 0.00 | 0.00 | 0.00 | 0.00 | 0.04 | 0.00 | 1.40 | 0.00 | 0.00 | 0.05 | 0.00 | 0.00 | Myb-related protein Myb4;Transcription repressor involved in regulation of protection against UV. |
| Cs2g_pb006280 | 0.00 | 1.34 | 0.00 | 0.00 | 0.00 | 0.00 | 38.27 | 0.00 | 3.81 | 0.00 | 0.00 | 0.00 | Probable inactive dual specificity protein phosphatase-like At4g18593 |
| Cs2g_pb006920 | 0.00 | 8.40 | 0.00 | 0.00 | 0.00 | 0.00 | 174.14 | 0.00 | 0.00 | 0.00 | 0.00 | 0.00 |  |
| Cs2g_pb008500 | 0.00 | 1.41 | 0.00 | 0.42 | 1.13 | 2.14 | 19.50 | 24.96 | 25.21 | 0.00 | 0.85 | 5.52 | STM |
| Cs2g_pb008950 | 0.17 | 0.00 | 0.00 | 0.00 | 0.00 | 0.00 | 2.43 | 0.00 | 0.00 | 0.00 | 0.00 | 0.00 |  |
| Cs2g_pb009750 | 0.00 | 0.00 | 0.00 | 0.14 | 0.00 | 0.00 | 1.07 | 0.06 | 0.09 | 0.16 | 0.00 | 0.00 | Mavicyanin; electron transfer activity |
| Cs2g_pb014290 | 0.00 | 0.00 | 0.00 | 0.00 | 0.00 | 0.00 | 7.39 | 0.00 | 0.00 | 0.00 | 0.00 | 0.00 |  |
| Cs2g_pb015260 | 0.00 | 0.00 | 0.00 | 0.00 | 0.00 | 0.00 | 149.72 | 0.00 | 0.00 | 0.00 | 0.00 | 0.00 |  |
| Cs2g_pb015620 | 0.00 | 0.00 | 0.47 | 0.00 | 0.00 | 0.00 | 10.50 | 0.00 | 0.00 | 0.00 | 0.00 | 0.00 |  |
| Cs2g_pb016670 | 0.27 | 0.12 | 0.00 | 0.00 | 0.03 | 0.09 | 77.90 | 1.39 | 0.67 | 0.93 | 0.87 | 0.55 | U4/U6 small nuclear ribonucleoprotein Prp31;especially under cold stress. May play a role in stress response. Involved in transcriptional gene silencing of endogenous transposable elements, independently of the RNA-directed DNA methylation (RdDM) pathway. |
| Cs2g_pb017460 | 0.00 | 0.00 | 0.00 | 0.03 | 0.02 | 0.02 | 13.35 | 0.12 | 0.08 | 0.17 | 0.07 | 0.07 | Pyrophosphate--fructose 6-phosphate 1-phosphotransferase subunit beta 1; photosynthesis; response to cadmium ion; response to cytokinin |
| Cs2g_pb018240 | 0.13 | 0.00 | 0.17 | 0.00 | 0.00 | 0.07 | 1.46 | 0.00 | 0.00 | 0.07 | 0.00 | 0.04 | Cytochrome b561 and DOMON domain-containing protein At5g47530; May act as a catecholamine-responsive trans-membrane electron transporter. |
| Cs2g_pb019500 | 0.00 | 0.00 | 0.00 | 0.00 | 0.00 | 0.00 | 4.53 | 0.00 | 0.00 | 0.51 | 0.00 | 0.00 | Non-specific lipid-transfer protein 13; Plant non-specific lipid-transfer proteins transfer phospholipids as well as galactolipids across membranes. May play a role in wax or cutin deposition in the cell walls of expanding epidermal cells and certain secretory tissues |
| Cs2g_pb020250 | 0.00 | 0.00 | 0.00 | 0.00 | 0.00 | 0.00 | 21.82 | 0.00 | 0.00 | 0.00 | 0.00 | 0.00 | Rac-like GTP-binding protein ARAC9; small GTPase mediated signal transduction |
| Cs2g_pb021040 | 0.40 | 0.67 | 0.13 | 0.44 | 1.85 | 0.79 | 15.43 | 0.25 | 0.79 | 2.64 | 0.06 | 0.70 | Early nodulin-like protein 1; electron transfer activity |
| Cs2g_pb022310 | 0.74 | 0.70 | 6.44 | 0.00 | 1.28 | 0.23 | 44.11 | 0.00 | 0.16 | 0.00 | 0.00 | 0.79 |  |
| Cs2g_pb026610 | 0.06 | 0.05 | 0.05 | 0.40 | 0.00 | 0.16 | 32.83 | 0.28 | 0.12 | 5.63 | 2.55 | 0.57 |  |
| Cs3g_pb000140 | 0.12 | 0.12 | 0.00 | 0.06 | 0.25 | 0.40 | 2.80 | 0.22 | 0.44 | 0.20 | 0.28 | 0.41 | Endoglucanase 25; Required for cellulose microfibrils formation. Involved in cell wall assembly during cell elongation and cell plate maturation in cytokinesis. Required for secondary cell wall formation in the developing xylem. May cycle through different intracellular compartments, including plasma membrane. |
| Cs3g_pb000800 | 0.00 | 0.00 | 0.00 | 0.00 | 0.00 | 0.00 | 42.23 | 0.00 | 0.00 | 0.00 | 0.00 | 0.00 | 22.0 kDa heat shock protein; response to heat |
| Cs3g_pb002810 | 0.00 | 0.04 | 0.04 | 0.00 | 0.32 | 0.08 | 2.52 | 0.06 | 0.01 | 0.11 | 0.04 | 0.05 | Alpha-galactosidase; Hydrolysis of terminal, non-reducing alpha-D-galactose residues in alpha-D-galactosides, including galactose oligosaccharides, galactomannans and galactolipids. |
| Cs3g_pb005470 | 0.00 | 0.34 | 0.00 | 0.00 | 0.00 | 0.00 | 2.72 | 0.00 | 0.00 | 0.00 | 0.00 | 0.00 |  |
| Cs3g_pb006220 | 0.00 | 0.00 | 0.00 | 0.00 | 0.00 | 0.00 | 1.56 | 0.00 | 0.00 | 0.00 | 0.00 | 0.00 |  |
| Cs3g_pb007860 | 0.00 | 0.00 | 0.14 | 0.00 | 0.00 | 0.00 | 7.98 | 0.44 | 0.31 | 1.09 | 0.07 | 0.06 | RNA polymerase II C-terminal domain phosphatase-like 4 |
| Cs3g_pb010040 | 0.00 | 0.00 | 0.00 | 0.00 | 0.00 | 0.00 | 1.10 | 0.04 | 0.00 | 0.00 | 0.00 | 0.00 | NAC domain-containing protein 102; May be involved in regulation of seed germination under flooding |
| Cs3g_pb010560 | 0.00 | 0.00 | 0.00 | 0.00 | 0.12 | 0.00 | 17.57 | 0.00 | 0.00 | 0.31 | 0.00 | 0.00 |  |
| Cs3g_pb011040 | 0.00 | 0.00 | 0.00 | 0.00 | 0.00 | 0.00 | 3.57 | 0.00 | 0.00 | 0.00 | 0.00 | 0.00 | Histone deacetylase 6; histone deacetylation; vegetative to reproductive phase transition of meristem; response to abscisic acid |
| Cs3g_pb014800 | 0.26 | 0.00 | 1.07 | 0.00 | 0.61 | 0.25 | 59.87 | 0.05 | 0.12 | 0.88 | 3.58 | 10.65 | WEB family protein At1g75720; chloroplast avoidance movement |
| Cs3g_pb017770 | 0.00 | 0.39 | 1.78 | 8.97 | 0.00 | 1.59 | 86.14 | 0.36 | 0.29 | 6.24 | 0.00 | 3.56 |  |
| Cs3g_pb019290 | 0.00 | 1.00 | 0.32 | 0.74 | 0.00 | 16.26 | 2240.03 | 11.49 | 69.52 | 134.13 | 77.51 | 186.91 | Egg cell-secreted protein 1.1 |
| Cs3g_pb021810 | 0.00 | 0.14 | 0.00 | 0.00 | 0.00 | 0.11 | 2.41 | 0.35 | 0.25 | 0.20 | 0.00 | 0.03 | Protein terminal ear1 homolog; Probable RNA-binding protein. Involved in the regular timing (plastochron) of lateral organs formation. May regulate the rate of leaf initiation and the duration of vegetative phase. Seems to be redundant to the function of PLASTOCHRON1, but to act in an independent pathway. |
| Cs3g_pb025190 | 0.00 | 0.00 | 0.00 | 0.00 | 0.00 | 0.00 | 1.40 | 0.11 | 0.09 | 0.00 | 0.00 | 0.00 |  |
| Cs3g_pb028270 | 0.07 | 0.04 | 0.07 | 0.00 | 0.00 | 0.00 | 1.60 | 0.00 | 0.00 | 0.00 | 0.00 | 0.00 |  |
| Cs4g_pb002120 | 0.00 | 0.00 | 4.41 | 0.00 | 0.00 | 0.00 | 198.12 | 5.71 | 5.94 | 23.29 | 17.18 | 1.78 | Probable xyloglucan endotransglucosylase/hydrolase protein 23; induced by auxin and brassinolide. Up-regulated by abscisic acid (ABA). |
| Cs4g_pb004220 | 0.28 | 0.00 | 0.00 | 0.00 | 0.00 | 0.00 | 4.99 | 0.00 | 0.00 | 0.00 | 0.00 | 0.00 |  |
| Cs4g_pb007060 | 0.00 | 0.00 | 0.00 | 0.00 | 0.00 | 0.00 | 7.28 | 0.00 | 0.00 | 0.00 | 0.00 | 0.00 |  |
| Cs4g_pb008710 | 0.00 | 1.52 | 0.00 | 0.00 | 0.00 | 0.00 | 399.69 | 0.00 | 2.64 | 0.00 | 0.00 | 0.00 |  |
| Cs4g_pb009990 | 48.53 | 78.00 | 214.79 | 120.22 | 125.70 | 40.00 | 10459.10 | 1092.05 | 1130.27 | 702.17 | 911.33 | 1239.63 | 60S ribosomal protein L29-1 |
| Cs4g_pb010350 | 0.00 | 0.00 | 0.00 | 0.00 | 0.00 | 0.00 | 55.39 | 0.00 | 2.69 | 0.00 | 0.00 | 0.00 |  |
| Cs4g_pb013500 | 0.00 | 0.00 | 0.00 | 0.10 | 0.00 | 0.00 | 4.32 | 0.09 | 0.04 | 0.64 | 0.15 | 0.00 |  |
| Cs4g_pb017200 | 1646.43 | 1154.22 | 927.20 | 940.76 | 2170.30 | 964.27 | 27637.40 | 2180.66 | 2288.42 | 2935.95 | 3355.98 | 2934.58 | 40S ribosomal protein S30 |
| Cs4g_pb020500 | 0.00 | 6.89 | 0.91 | 6.15 | 0.00 | 3.39 | 126.09 | 0.00 | 0.00 | 0.00 | 0.00 | 0.00 | NADP-dependent alkenal double bond reductase P2; May play a distinct role in plant antioxidant defense and is possibly involved in NAD(P)/NAD(P)h homeostasis. |
| Cs4g_pb020990 | 0.38 | 0.22 | 0.04 | 0.02 | 0.01 | 0.04 | 5.82 | 0.11 | 0.17 | 0.26 | 0.01 | 0.01 | Glycerophosphodiester phosphodiesterase protein kinase domain-containing GDPDL2; Atypical receptor-like kinase involved in disease resistance |
| Cs4g_pb021280 | 0.00 | 0.00 | 0.00 | 0.00 | 0.00 | 0.00 | 2.56 | 0.12 | 0.19 | 0.00 | 0.00 | 0.22 | EG45-like domain containing protein; Plays a systemic role in water and solute homeostasis. |
| Cs4g_pb022170 | 0.00 | 0.00 | 0.00 | 0.44 | 0.00 | 0.00 | 37.41 | 2.84 | 3.11 | 1.04 | 0.32 | 0.04 | Monogalactosyldiacylglycerol synthase 2, chloroplastic; mediate galactolipid syntheses, Auxin activates expression during Pi starvation, whereas cytokinin represses it. |
| Cs4g_pb022500 | 0.82 | 0.39 | 0.09 | 0.45 | 0.31 | 0.14 | 46.52 | 0.10 | 0.21 | 0.55 | 0.39 | 0.03 |  |
| Cs4g_pb022990 | 0.08 | 0.34 | 0.03 | 0.04 | 0.10 | 0.00 | 3.83 | 0.09 | 0.00 | 0.14 | 0.04 | 0.06 | LysM domain receptor-like kinase 3; Putative Lysin motif (LysM) receptor kinase that may recognize microbe-derived N-acetylglucosamine (NAG)-containing ligands. |
| Cs4g_pb024540 | 19.43 | 29.27 | 8.74 | 9.01 | 18.04 | 4.75 | 213.65 | 10.89 | 7.88 | 30.56 | 9.20 | 10.20 | Probable transcriptional regulatory protein At2g25830 |
| Cs5g_pb001330 | 0.00 | 0.13 | 0.00 | 0.00 | 0.00 | 0.10 | 3.23 | 0.00 | 0.08 | 0.11 | 0.00 | 0.00 | Putative ripening-related protein 2 |
| Cs5g_pb001800 | 0.00 | 0.00 | 0.10 | 0.00 | 0.00 | 0.00 | 5.31 | 0.40 | 0.00 | 0.09 | 0.00 | 0.00 |  |
| Cs5g_pb002180 | 0.00 | 0.00 | 0.00 | 0.00 | 0.00 | 0.00 | 10.97 | 0.00 | 0.00 | 0.00 | 0.00 | 0.00 |  |
| Cs5g_pb004450 | 0.00 | 0.00 | 0.00 | 0.00 | 0.04 | 0.00 | 2.84 | 0.03 | 0.00 | 0.25 | 0.44 | 0.06 | Disease resistance-like protein CSA1;TIR-NB-LRR receptor-like protein that functions in photomorphogenic development. May function downstream of phytochrome B (phyB) signaling. |
| Cs5g_pb005030 | 0.00 | 0.00 | 0.00 | 0.00 | 0.00 | 0.00 | 7.93 | 0.00 | 0.00 | 0.00 | 0.00 | 0.00 |  |
| Cs5g_pb005110 | 0.02 | 0.00 | 0.00 | 0.08 | 0.00 | 0.00 | 3.91 | 0.04 | 0.07 | 0.12 | 0.05 | 0.05 | Probable receptor-like protein kinase At5g61350 |
| Cs5g_pb010580 | 0.00 | 0.44 | 0.05 | 0.00 | 0.13 | 0.16 | 4.35 | 0.02 | 0.16 | 0.07 | 0.38 | 0.30 | Homeobox protein knotted-1-like 6; Plays a role in meristem function. Contributes to the shoot apical meristem (SAM) maintenance and organ separation by controlling boundary establishment in embryo in a CUC1, CUC2 and STM-dependent manner. Involved in maintaining cells in an undifferentiated, meristematic state. Probably binds to the DNA sequence 5'-TGAC-3'. |
| Cs5g_pb011010 | 0.30 | 0.05 | 0.10 | 0.00 | 0.16 | 0.00 | 6.06 | 0.27 | 0.14 | 0.04 | 0.00 | 1.10 | Probable cyclic nucleotide-gated ion channel 16 |
| Cs5g_pb012260 | 46.51 | 43.27 | 49.88 | 36.49 | 31.18 | 30.38 | 263.30 | 47.96 | 55.51 | 43.49 | 59.90 | 78.26 | Pectinesterase 3; Acts in the modification of cell walls via demethylesterification of cell wall pectin. |
| Cs5g_pb012520 | 0.07 | 0.07 | 0.05 | 0.02 | 0.10 | 0.03 | 2.09 | 0.01 | 0.03 | 0.01 | 0.25 | 0.03 | Probable copper-transporting ATPase HMA5; Involved in copper import into the cell. May play a role in copper detoxification in roots. |
| Cs5g_pb012600 | 0.00 | 0.00 | 0.00 | 0.00 | 0.00 | 0.00 | 90.41 | 6.16 | 3.23 | 5.51 | 1.68 | 6.83 |  |
| Cs5g_pb013350 | 0.00 | 0.00 | 0.00 | 0.00 | 0.00 | 0.00 | 1.32 | 0.05 | 0.10 | 0.23 | 0.00 | 0.00 | Late embryogenesis abundant protein At1g64065 |
| Cs5g_pb013360 | 1.34 | 0.69 | 0.27 | 0.74 | 0.00 | 0.00 | 16.56 | 0.99 | 0.42 | 2.86 | 0.04 | 0.89 | Late embryogenesis abundant protein At1g64065 |
| Cs5g_pb014050 | 0.81 | 0.00 | 0.00 | 0.00 | 0.00 | 0.00 | 21.20 | 0.10 | 0.00 | 0.06 | 0.10 | 0.10 | RING-H2 finger protein ATL22; involved in the pathway protein ubiquitination |
| Cs5g_pb014130 | 0.00 | 0.00 | 0.00 | 0.00 | 0.00 | 0.00 | 1.13 | 0.00 | 0.00 | 0.00 | 0.00 | 0.00 |  |
| Cs5g_pb015010 | 0.27 | 0.47 | 0.43 | 0.25 | 0.31 | 0.00 | 39.59 | 3.46 | 2.98 | 1.02 | 4.68 | 5.97 | Pyruvate dehydrogenase E1 component subunit alpha-3, chloroplastic; The pyruvate dehydrogenase complex catalyzes the overall conversion of pyruvate to acetyl-CoA and CO2 |
| Cs5g_pb015100 | 0.00 | 0.00 | 0.00 | 0.00 | 0.00 | 0.00 | 45.47 | 0.00 | 0.00 | 0.00 | 0.00 | 0.00 | Alpha,alpha-trehalose-phosphate synthase [UDP-forming] 6;Required for normal embryo development, vegetative growth and transition to flowering. Regulates embryo growth, cell wall deposition, starch and sucrose degradation, but not cell differentiation. Involved in the regulation of glucose sensing and signaling genes during plant development. |
| Cs5g_pb020210 | 0.00 | 0.00 | 0.00 | 0.00 | 0.28 | 0.00 | 2.25 | 0.00 | 0.00 | 0.00 | 0.00 | 0.00 |  |
| Cs5g_pb020700 | 0.00 | 0.00 | 0.00 | 0.00 | 0.00 | 0.00 | 4.24 | 0.00 | 0.00 | 0.00 | 0.00 | 0.00 |  |
| Cs5g_pb025220 | 0.00 | 0.18 | 0.00 | 0.00 | 0.00 | 0.17 | 2.32 | 0.30 | 0.00 | 0.00 | 0.09 | 0.00 |  |
| Cs5g_pb031560 | 0.00 | 0.37 | 2.47 | 0.00 | 0.62 | 0.00 | 25.65 | 1.27 | 2.12 | 0.74 | 0.00 | 0.00 | Stigma-specific STIG1-like protein 3 |
| Cs5g_pb032470 | 0.00 | 0.00 | 0.00 | 0.00 | 0.00 | 0.00 | 2.01 | 0.00 | 0.00 | 0.00 | 0.00 | 0.00 |  |
| Cs5g_pb033600 | 0.00 | 0.00 | 0.00 | 0.00 | 0.00 | 0.00 | 6.03 | 0.00 | 0.00 | 0.00 | 0.00 | 0.00 |  |
| Cs6g_pb001230 | 0.00 | 0.04 | 0.00 | 0.00 | 0.00 | 0.12 | 1.33 | 0.00 | 0.07 | 0.00 | 0.03 | 0.10 | Protein NRT1/ PTR FAMILY 8.1; Peptide transporter. |
| Cs6g_pb001310 | 0.00 | 0.13 | 0.03 | 0.00 | 0.07 | 0.03 | 8.04 | 0.02 | 0.04 | 0.06 | 0.06 | 0.00 | Protein NRT1/ PTR FAMILY 5.5; transmembrane transporter activity |
| Cs6g_pb004130 | 0.00 | 0.21 | 0.00 | 0.00 | 0.00 | 0.00 | 9.40 | 0.00 | 0.00 | 0.00 | 0.00 | 0.00 |  |
| Cs6g_pb008880 | 0.00 | 1.14 | 1.36 | 0.17 | 0.54 | 0.37 | 28.55 | 0.00 | 0.00 | 0.14 | 0.18 | 0.19 | E3 ubiquitin-protein ligase SINA-like 10; mediates ubiquitination and subsequent proteasomal degradation of target proteins. |
| Cs6g_pb010520 | 0.00 | 0.00 | 0.00 | 0.00 | 0.03 | 0.00 | 2.37 | 0.42 | 0.01 | 0.04 | 0.00 | 0.13 | NAC transcription factor NAM-1 |
| Cs6g_pb012960 | 0.00 | 0.10 | 0.00 | 0.00 | 0.34 | 0.14 | 3.91 | 0.17 | 0.09 | 0.69 | 0.00 | 0.06 |  |
| Cs7g_pb001680 | 0.07 | 0.00 | 0.00 | 0.00 | 0.07 | 0.10 | 2.44 | 0.04 | 0.11 | 0.14 | 0.02 | 0.00 | Tobamovirus multiplication protein 2A |
| Cs7g_pb002860 | 0.00 | 0.00 | 0.00 | 0.00 | 0.00 | 0.00 | 4.69 | 0.00 | 0.00 | 0.00 | 0.00 | 0.00 |  |
| Cs7g_pb004070 | 2.31 | 1.86 | 1.36 | 1.56 | 6.18 | 4.68 | 133.73 | 2.18 | 6.37 | 7.18 | 2.27 | 15.71 | Extracellular ribonuclease LE; Probably involved in plant phosphate-starvation rescue system. |
| Cs7g_pb005130 | 0.18 | 0.00 | 0.03 | 0.00 | 0.03 | 0.10 | 2.19 | 0.02 | 0.01 | 0.03 | 0.33 | 0.09 | Beta-hexosaminidase 2 |
| Cs7g_pb007890 | 0.00 | 0.00 | 0.00 | 0.00 | 0.00 | 0.00 | 1.18 | 0.00 | 0.00 | 0.00 | 0.00 | 0.00 |  |
| Cs7g_pb012100 | 1.34 | 0.00 | 0.59 | 0.80 | 0.58 | 0.00 | 141.23 | 0.00 | 0.00 | 0.00 | 0.00 | 1.06 |  |
| Cs7g_pb014920 | 0.00 | 0.00 | 0.00 | 0.00 | 0.00 | 0.00 | 1.36 | 0.00 | 0.00 | 0.00 | 0.00 | 0.00 |  |
| Cs7g_pb017350 | 0.00 | 0.00 | 0.00 | 0.00 | 0.00 | 0.00 | 4.69 | 0.00 | 0.00 | 0.00 | 0.00 | 0.00 |  |
| Cs7g_pb019370 | 0.00 | 0.18 | 0.00 | 0.00 | 0.00 | 0.07 | 3.15 | 0.50 | 0.15 | 0.30 | 0.00 | 0.00 | TATA box-binding protein-like protein 2 |
| Cs7g_pb020050 | 0.00 | 1.73 | 0.00 | 0.13 | 0.00 | 0.21 | 13.20 | 0.11 | 0.05 | 0.72 | 0.42 | 0.06 | BTB/POZ domain-containing protein At3g56230; May act as a substrate-specific adapter of an E3 ubiquitin-protein ligase complex (CUL3-RBX1-BTB) which mediates the ubiquitination and subsequent proteasomal degradation of target proteins. |
| Cs7g_pb021950 | 0.00 | 0.00 | 0.00 | 0.00 | 0.00 | 0.00 | 3.72 | 0.00 | 0.00 | 0.00 | 0.00 | 0.00 |  |
| Cs7g_pb022250 | 0.00 | 0.00 | 0.20 | 0.08 | 0.16 | 0.17 | 3.03 | 0.03 | 0.00 | 0.00 | 0.11 | 0.06 | Expansin-A6; Causes loosening and extension of plant cell walls by disrupting non-covalent bonding between cellulose microfibrils and matrix glucans. |
| Cs7g_pb022900 | 0.00 | 0.00 | 0.00 | 0.00 | 0.00 | 0.00 | 9.64 | 0.00 | 0.00 | 0.00 | 0.00 | 0.00 |  |
| Cs7g_pb023380 | 0.00 | 0.00 | 0.00 | 0.00 | 0.00 | 0.00 | 2.71 | 0.00 | 0.00 | 0.02 | 0.00 | 0.00 | UPF0481 protein At3g47200 |
| Cs7g_pb023580 | 0.00 | 0.00 | 0.11 | 0.00 | 0.00 | 0.05 | 1.07 | 0.05 | 0.00 | 0.19 | 0.10 | 0.03 | Peroxidase 15 |
| Cs7g_pb023850 | 0.00 | 0.00 | 0.00 | 0.07 | 0.05 | 0.05 | 4.70 | 0.17 | 0.20 | 0.97 | 0.00 | 0.06 | Probable terpene synthase 6 |
| Cs7g_pb024420 | 1.44 | 0.65 | 0.00 | 3.85 | 7.57 | 1.30 | 75.53 | 4.17 | 3.10 | 3.56 | 0.00 | 0.00 | Transcription elongation factor 1 homolog |
| Cs7g_pb025430 | 0.78 | 9.46 | 0.97 | 0.99 | 0.00 | 0.58 | 79.54 | 0.90 | 3.00 | 0.19 | 0.09 | 0.77 | BTB/POZ domain-containing protein At3g56230; May act as a substrate-specific adapter of an E3 ubiquitin-protein ligase complex (CUL3-RBX1-BTB) which mediates the ubiquitination and subsequent proteasomal degradation of target proteins. |
| Cs7g_pb026610 | 0.30 | 0.07 | 0.10 | 0.00 | 0.00 | 0.00 | 7.34 | 0.02 | 0.06 | 0.00 | 0.00 | 0.00 |  |
| Cs8g_pb005040 | 0.00 | 0.05 | 0.00 | 0.00 | 0.00 | 0.00 | 1.53 | 0.04 | 0.00 | 0.12 | 0.00 | 0.03 |  |
| Cs8g_pb006630 | 0.00 | 0.00 | 0.00 | 0.00 | 0.00 | 0.00 | 1.41 | 0.00 | 0.19 | 0.00 | 0.00 | 0.00 |  |
| Cs8g_pb008280 | 0.00 | 0.00 | 0.00 | 0.00 | 0.00 | 0.00 | 1.12 | 0.00 | 0.00 | 0.00 | 0.00 | 0.00 | Defensin-like protein 19 |
| Cs8g_pb011070 | 0.83 | 0.16 | 0.00 | 0.00 | 0.00 | 0.00 | 18.06 | 0.00 | 0.00 | 0.00 | 0.00 | 0.00 | Peroxiredoxin-2B; cell redox homeostasis |
| Cs8g_pb012280 | 0.00 | 0.00 | 0.00 | 0.00 | 0.00 | 0.09 | 1.02 | 0.00 | 0.00 | 0.00 | 0.04 | 0.00 |  |
| Cs8g_pb015730 | 0.00 | 0.29 | 0.18 | 0.00 | 1.27 | 0.65 | 14.94 | 0.02 | 0.33 | 0.02 | 0.57 | 0.15 | Cation/H(+) antiporter 25; May operate as a cation/H+ antiporter. |
| Cs9g_pb000040 | 0.00 | 0.00 | 1.76 | 0.00 | 0.81 | 0.00 | 53.77 | 0.00 | 0.00 | 0.00 | 0.00 | 0.00 |  |
| Cs9g_pb001280 | 2.28 | 4.10 | 0.00 | 3.91 | 1.68 | 0.08 | 94.84 | 5.28 | 2.81 | 19.56 | 0.00 | 0.07 | Dirigent protein 11; Dirigent proteins impart stereoselectivity on the phenoxy radical-coupling reaction, yielding optically active lignans from two molecules of coniferyl alcohol in the biosynthesis of lignans, flavonolignans, and alkaloids and thus plays a central role in plant secondary metabolism. |
| Cs9g_pb004750 | 0.00 | 0.00 | 0.00 | 0.00 | 0.00 | 0.00 | 3.02 | 0.00 | 0.00 | 0.21 | 0.14 | 0.00 |  |
| Cs9g_pb006500 | 0.00 | 0.14 | 0.00 | 0.05 | 0.00 | 0.00 | 3.56 | 0.09 | 0.12 | 0.01 | 0.18 | 0.04 | Mannan endo-1,4-beta-mannosidase 2; mannan metabolic process-cell wall |
| Cs9g_pb006760 | 0.00 | 0.00 | 0.00 | 0.00 | 0.00 | 0.00 | 4.59 | 0.05 | 0.00 | 0.21 | 0.19 | 0.07 | Ankyrin repeat-containing protein At3g12360;Involved in salt stress tolerance. May act through abscisic acid (ABA) signaling pathways and promote reactive oxygen species (ROS) production. |
| Cs9g_pb006780 | 0.02 | 0.00 | 0.00 | 0.00 | 0.00 | 0.00 | 3.86 | 0.00 | 0.00 | 0.02 | 0.00 | 0.00 | Ankyrin repeat-containing protein At3g12360 |
| Cs9g_pb006820 | 0.02 | 0.00 | 0.00 | 0.00 | 0.00 | 0.00 | 1.06 | 0.00 | 0.00 | 0.07 | 0.00 | 0.00 | Ankyrin repeat-containing protein At5g02620 |
| Cs9g_pb006910 | 0.00 | 0.00 | 0.00 | 0.00 | 0.00 | 0.00 | 2.93 | 0.00 | 0.00 | 0.00 | 0.00 | 0.00 | Ankyrin repeat-containing protein At3g12360 |
| Cs9g_pb006930 | 0.02 | 0.00 | 0.06 | 0.00 | 0.00 | 0.00 | 4.80 | 0.00 | 0.00 | 0.06 | 0.00 | 0.01 | Ankyrin repeat-containing protein At3g12360 |
| Cs9g_pb007240 | 1.26 | 0.00 | 0.00 | 0.00 | 0.00 | 0.00 | 21.32 | 0.41 | 1.58 | 1.99 | 0.00 | 0.00 |  |
| Cs9g_pb013730 | 0.00 | 0.00 | 0.00 | 0.00 | 0.00 | 0.00 | 80.74 | 0.00 | 0.00 | 0.00 | 0.00 | 0.00 |  |
| Cs9g_pb013900 | 0.00 | 0.00 | 0.06 | 0.00 | 0.05 | 0.00 | 1.60 | 0.00 | 0.02 | 0.00 | 0.00 | 0.00 | Zeatin O-glucosyltransferase; regulation of vegetative meristem growth-morphogenesis, cytokinin metabolic process |
| Cs9g_pb013910 | 0.03 | 0.00 | 0.00 | 0.00 | 0.00 | 0.03 | 12.19 | 0.00 | 0.02 | 0.00 | 0.00 | 0.00 |  |
| Cs9g_pb015430 | 0.00 | 0.00 | 0.00 | 0.00 | 0.00 | 0.11 | 1.31 | 0.00 | 0.00 | 0.00 | 0.18 | 0.00 |  |
| Cs9g_pb016600 | 0.00 | 0.00 | 0.00 | 0.00 | 0.00 | 0.00 | 32.36 | 0.00 | 0.00 | 0.00 | 0.00 | 0.00 |  |
| Cs9g_pb017010 | 0.00 | 0.00 | 0.00 | 0.00 | 0.00 | 0.13 | 8.46 | 0.06 | 0.00 | 1.26 | 0.00 | 0.01 | BI1-like protein |
| Cs9g_pb018290 | 0.84 | 0.41 | 0.00 | 0.00 | 0.00 | 0.13 | 10.55 | 0.12 | 0.00 | 0.69 | 0.00 | 0.19 | Transcription factor bHLH36 |
| Cs9g_pb020800 | 0.09 | 0.21 | 0.36 | 0.02 | 1.40 | 0.61 | 18.05 | 1.00 | 0.76 | 3.92 | 2.03 | 1.66 | Cyclic nucleotide-gated ion channel 1; Can be activated by cyclic AMP which leads to an opening of the cation channel. May be responsible for cAMP-induced calcium entry in cells and thus should be involved in the calcium signal transduction. Could transport K+, Na+ and Pb2+. |
| Cs9g_pb020950 | 0.00 | 0.00 | 0.00 | 0.00 | 0.00 | 0.00 | 1.68 | 0.00 | 0.00 | 0.00 | 0.00 | 0.00 | Inorganic phosphate transporter 1-11 |
| CsUn_pb001290 | 0.00 | 0.00 | 0.00 | 0.00 | 0.00 | 0.00 | 1.73 | 0.00 | 0.10 | 0.00 | 0.00 | 0.00 |  |
| CsUn_pb002600 | 0.00 | 0.00 | 0.00 | 0.00 | 0.00 | 0.00 | 1.09 | 0.00 | 0.00 | 0.00 | 0.00 | 0.00 |  |
| CsUn_pb004290 | 0.00 | 0.00 | 0.00 | 0.00 | 0.00 | 0.00 | 38.27 | 0.00 | 0.00 | 0.00 | 0.00 | 0.00 |  |
| CsUn_pb005400 | 0.00 | 0.00 | 0.00 | 0.00 | 0.00 | 0.00 | 1.15 | 0.00 | 0.14 | 0.00 | 0.05 | 0.00 |  |
| CsUn_pb012700 | 0.00 | 0.00 | 0.00 | 0.00 | 0.00 | 0.00 | 3.05 | 0.00 | 0.00 | 0.00 | 0.00 | 0.00 |  |
| CsUn_pb031870 | 0.36 | 0.00 | 0.00 | 0.00 | 0.24 | 0.00 | 4.38 | 0.66 | 0.00 | 0.00 | 0.00 | 0.27 | Auxin-induced protein 15A; auxin-activated signaling pathway |
| CsUn_pb032200 | 0.00 | 0.00 | 0.00 | 0.00 | 0.00 | 0.00 | 2.51 | 0.00 | 0.00 | 0.00 | 0.00 | 0.00 |  |
| CsUn_pb033390 | 0.00 | 0.86 | 0.47 | 0.00 | 0.00 | 0.00 | 15.28 | 0.34 | 0.57 | 0.73 | 0.00 | 1.20 |  |
| CsUn_pb033740 | 0.70 | 0.38 | 0.94 | 0.13 | 1.39 | 0.11 | 17.06 | 1.15 | 0.91 | 2.37 | 0.39 | 0.29 |  |
| CsUn_pb034130 | 47.13 | 63.55 | 66.14 | 67.10 | 57.88 | 67.09 | 423.20 | 49.29 | 48.93 | 48.05 | 70.35 | 100.70 | Guanine nucleotide-binding protein subunit gamma 1; Guanine nucleotide-binding proteins (G proteins) are involved as a modulator or transducer in various transmembrane signaling systems. |
| CsUn_pb052810 | 1.47 | 0.00 | 0.22 | 0.00 | 0.07 | 0.19 | 16.70 | 0.21 | 0.21 | 1.19 | 0.00 | 0.24 |  |

| **Table S9. Primers used in this study for validations of TE insertions.** | | |
| --- | --- | --- |
| **Primer name** | **Primer sequence (5'→3')** | |
| TE_in_Cs2g_pb006570 | Forward: CAGAGATTTGATGGCATTGATAAGA | Reverse: GGTAAGGTGGGCATTGTAGGAGGTC |
| TE_in_Cs2g_pb010960 | Forward: GGCCGGTTACTCACTATATGATCGA | Reverse: TTACTTGGAGAAGGTGTTGTGGGAC |
| TE_in_Cs2g_pb020210 | Forward: AAATAGAAGGCTTTTACTTTCAGAG | Reverse: ATGAGTTCAACGAGAACACTAGAGA |
| TE_in_Cs4g_pb022990 | Forward: TTTCTTCACAGTGAGTCAAGTTGG | Reverse: TAGCCTTTTGTTCTTTTCTTATTT |
| TE_into_chr2:4879334 | Forward: TAACAACCTTCACACACGACTCCT | Reverse: TCTTTCTTTTTGGTCCTTAACCAA |
| TE_into_chr8:3585305 | Forward: TATAAATAAATTTTTGAGCTAGTC | Reverse: TCTTTCTTCTTTGCATTATCATCG |
| TE_into_chr1:23537730 | Forward: ATCATAATCCTTTAAAAAGCCATG | Reverse: CCGATAAAGCTCTTCTTAAGTCAT |
